# Poly-histidine grafting leading to fishbone-like architectures†

**DOI:** 10.1039/c8ra00315g

**Published:** 2018-02-26

**Authors:** Vincenzo Razzano, Marco Paolino, Annalisa Reale, Germano Giuliani, Alessandro Donati, Gianluca Giorgi, Roberto Artusi, Gianfranco Caselli, Michela Visintin, Francesco Makovec, Salvatore Battiato, Filippo Samperi, Francesca Villafiorita-Monteleone, Chiara Botta, Andrea Cappelli

**Affiliations:** Dipartimento di Biotecnologie, Chimica e Farmacia, European Research Centre for Drug Discovery and Development, Università di Siena Via A. Moro 2 53100 Siena Italy andrea.cappelli@unisi.it vincy_box@hotmail.it paomar@oneonline.it reale5@student.unisi.it germix67@hotmail.com alessandro.donati@unisi.it gianluca.giorgi@unisi.it +39 577 234320; Rottapharm Biotech S.p.A. Via Valosa di Sopra 9 20900 Monza Italy Roberto.Artusi@rottapharmbiotech.com Gianfranco.Caselli@rottapharmbiotech.com Michela.Visintin@rottapharmbiotech.com Francesco.Makovec@rottapharmbiotech.com; Istituto per i Polimeri, Compositi e Biomateriali (IPCB), U.O.S. di Catania, CNR Via Gaifami 18 95126 Catania Italy salvatore.battiato@cnr.it fsamperi@unict.it; Istituto per lo Studio delle Macromolecole (CNR) Via A. Corti 12 20133 Milano Italy villafiorita@ismac.cnr.it chiara.botta@ismac.cnr.it

## Abstract

A small series of Morita–Baylis–Hillman adduct (MBHA) derivatives was synthesized and made to react with imidazole, *N*-acetylhistidine, and *N*-acetylhexahistidine as models of poly-histidine derivatives. Intriguingly, the reaction of MBHA derivatives 1a and b with imidazole in acetonitrile–phosphate buffered saline (PBS) gave the imidazolium salt biadducts 3a and b as the main reaction products. These results were confirmed by experiments performed with *N*-acetylhistidine and 1b and suggested the possible occurrence of these structures in the products of poly-histidine labeling with MBHA derivatives 1a and b. These compounds were then transformed into the corresponding water-soluble derivatives 1c–e by introducing oligo(ethylene glycol) chains and their reactivity was evaluated in preliminary experiments with imidazole and then with *N*-acetylhexahistidine in PBS. The structure of polymeric materials Ac-His-6-MBHA-1d and Ac-His-6-MBHA-1e obtained using ten-fold excesses of compounds 1d and e was investigated using mass spectrometry, NMR spectroscopy, and photophysical studies, which suggested the presence of biadduct residues in both polymeric materials. These results provide the basis for the preparation of fishbone-like polymer brushes, the characterization of their properties, and the exploration of their potential applications in different fields of science such as *in vivo* fluorogenic labeling, fluorescence microscopy, protein PEGylation, up to the production of smart materials and biosensors.

## Abstract 2

A small series of Morita–Baylis–Hillman derivatives was synthesized and made to react with *N*-acetylhexahistidine to give polymeric materials characterized by the presence of biadduct residues.

## Graphical abstract

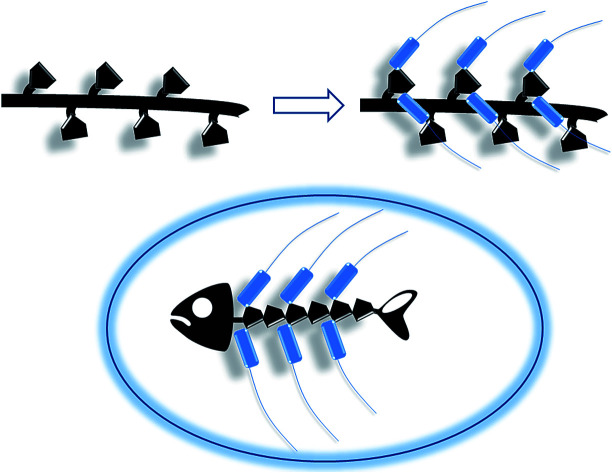

## Introduction

Histidine is an amino acid of crucial importance in many life processes. The imidazole residue confers peculiar characteristics to this amino acid and its derivatives.^1^ In fact, thanks to the imidazole p*K*_a_ (about 6.0), histidine plays an important role as a buffer within the cellular environment (usually neutral) due to annular nitrogen protonation.^2,3^ Moreover, histidine is capable of complexing metal ions and is thus involved in many catalytic sites.^4^ Not uncommon, histidine is used in the synthesis of aminoacidic polymers. Poly-histidine (poly-His) showing different molecular weight was synthesized by controlled ring opening polymerization of l-histidine-*N*-carboxyanhydride.^2,5^ These polymers have been studied as such or used as functional side chain of other polymers (such as poly-lysine) or as fragments in block copolymers with polyethylene glycol (PEG) and acrylic polymers.^6–8^ Most histidine polymers have in common a high biocompatibility, a good fusogenic activity, a buffering capacity, and a peculiar proton-sponge effect. By virtue of these properties, they are often used as non-toxic gene delivery agents and investigated as component of drug delivery systems for intracellular cytotoxic drugs.^6,7,9^ In fact, the presence of imidazole residues ensures, at physiological pH, the presence of positive charges on the surface of the polymers that promotes adhesion to cell membranes and internalization by endocytosis of complexes polymer/pDNA (poliplex) or polymer/drug. In addition, the proton-sponge effect inactivates lysosomal enzymes and the fusogenic activity allows the endosome escape.^10,11^

However, the systemic administration of the poly-histidine derivatives (and of cationic polymers in general) may show some complications. In fact, the cationic surface charge of this type of vectors promotes many nonspecific interactions with cellular components of blood, of the endothelium of blood vessels and plasma protein, dramatically reducing the plasma half-life and biodistribution. To overcome this problem, it has been developed the “shielding strategy”, which consists in protecting the residual surface charge with a steric barrier.^12^ This strategy is feasible by means of the conjugation to poly-histidines of neutral hydrophilic polymers such as the polyethylene glycol (PEG), which confers an increase in the solubility, a reduced interaction with serum proteins (*e.g.* albumin), improved biocompatibility, and a prolonged blood circulation.^13^

Furthermore, the insertion of poly-histidine tags in recombinant proteins represents a standard methodology in isolation and purification of recombinant proteins. Furthermore, their presence in the protein sequence provides an interesting opportunity for the selective covalent functionalization of these engineered proteins.^14–18^

Although alkylation of histidine residues in poly-histidine derivatives could be a difficult task owing to the low reactivity of imidazole rings in conventional alkylation reactions, a successful example of *N*-acetylhexahistidine (Ac-His-6) alkylation with Morita–Baylis–Hillman adduct (MBHA) derivatives was accomplished by using transition metal cations interacting with a nitrilotriacetate ligand linked to the leaving group of MBHA derivative.^19^

**Fig. 1:**
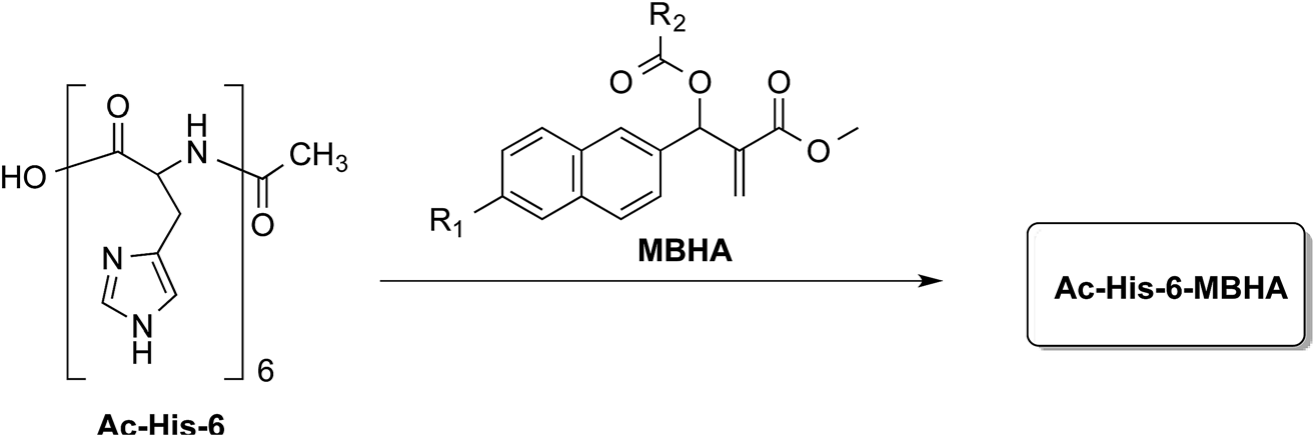
Grafting *N*-acetylhexahistidine (Ac-His-6) with Morita–Baylis–Hillman adduct (MBHA) derivatives.

In order to explore the synthesis, the structure, and the properties of graft copolymers of poly-histidine with fluorescent molecules, a small series of MBHA derivatives^20^ were designed and synthesized to be evaluated in the reaction with Ac-His-6 as models of poly-histidine species (Fig. 1).

Thus in the work described herein, a small series of MBHA derivatives were designed, synthesized, and made to react with imidazole in order to evaluate their reactivity and the photophysical properties of the corresponding imidazole derivatives. The most promising compounds were then evaluated in their reaction with Ac-His-6 and the structural and photophysical features of the corresponding graft copolymers were analyzed in details.

## Results and discussion

### Synthesis

**Scheme 1:**
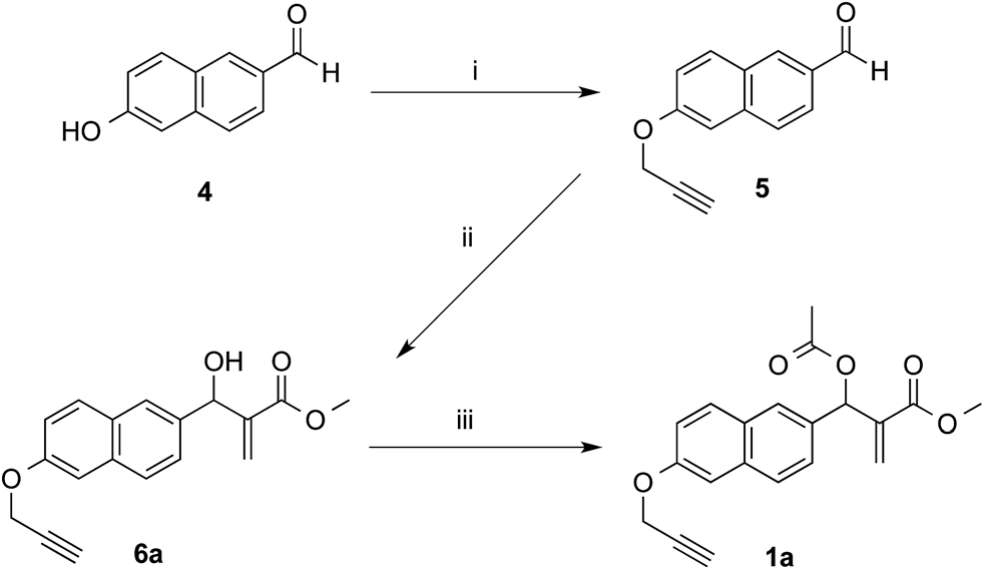
Synthesis of MBHA derivative 1a. Reagents: (i) propargyl bromide, K_2_CO_3_, acetonitrile; (ii) methyl acrylate, DABCO, CH_3_OH, THF; (iii) CH_3_COCl, TEA, CH_2_Cl_2_.

**Scheme 2:**
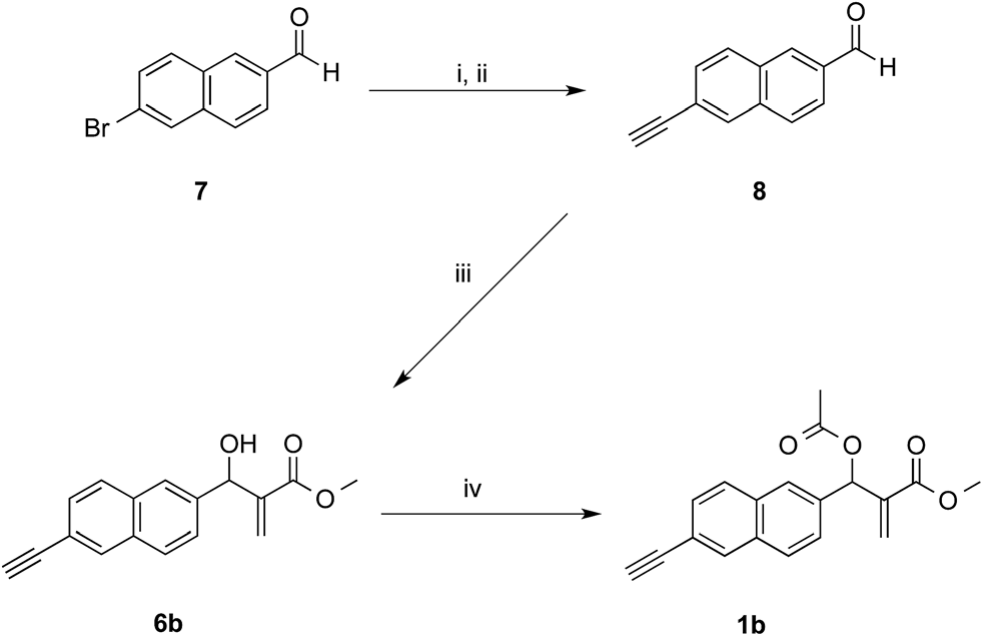
Synthesis of MBHA derivative 1b. Reagents: (i) ethynyltrimethylsilane, Pd(PPh_3_)_2_Cl_2_, CuI, TEA, THF; (ii) K_2_CO_3_, MeOH; (iii) DABCO, methyl acrylate, MeOH, THF; (iv) CH_3_COCl, TEA, CH_2_Cl_2_.

The synthetic works started with the preparation of MBHA derivatives 1a,b from 6-hydroxy-2-naphthaldehyde (4) or 6-bromo-2-naphthaldehyde (7), respectively (Schemes 1 and 2).

**Scheme 3:**
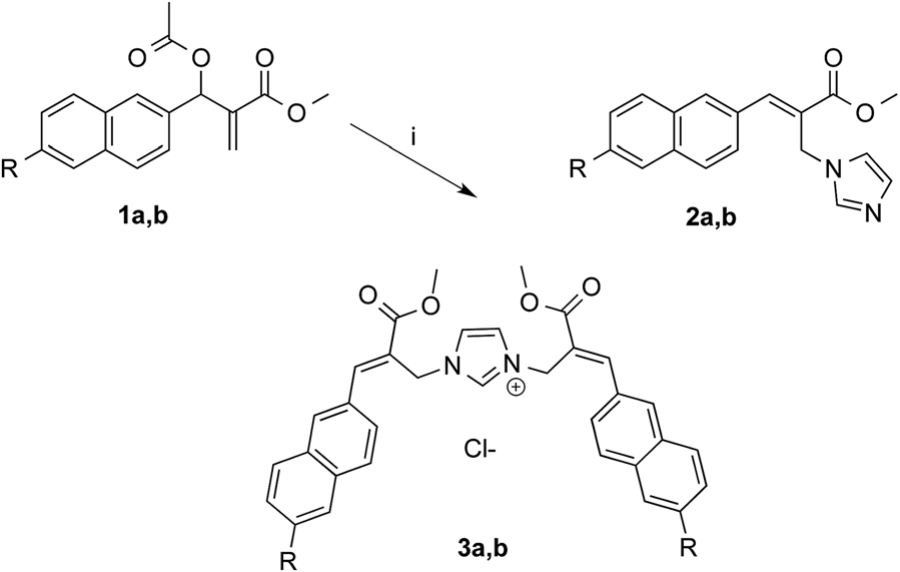
Reaction of MBHA derivatives 1a,b with imidazole. Reagents: (i) imidazole, CH_3_CN, PBS. Substituents: R = –OCH_2_CCH (1a, 2a, 3a); R = –CCH (1b, 2b, 3b).

As expected, the reaction of naphthalene MBHA derivative 1a with 1.5 equivalents of imidazole in THF–water (5 : 1) under reflux afforded imidazole derivative 2a in 78% yield. Similar yields (*i.e.* 80%) were obtained when 1a was reacted with 6 equivalents of imidazole in acetonitrile–phosphate buffered saline (PBS) as the reaction medium at room temperature for 24 h. On the other hand, when 1.5 equivalents of imidazole were used in the same reaction medium, along the expected imidazole derivative 2a, imidazolium salt 3a was isolated as the main reaction product (Scheme 3).

**Fig. 2:**
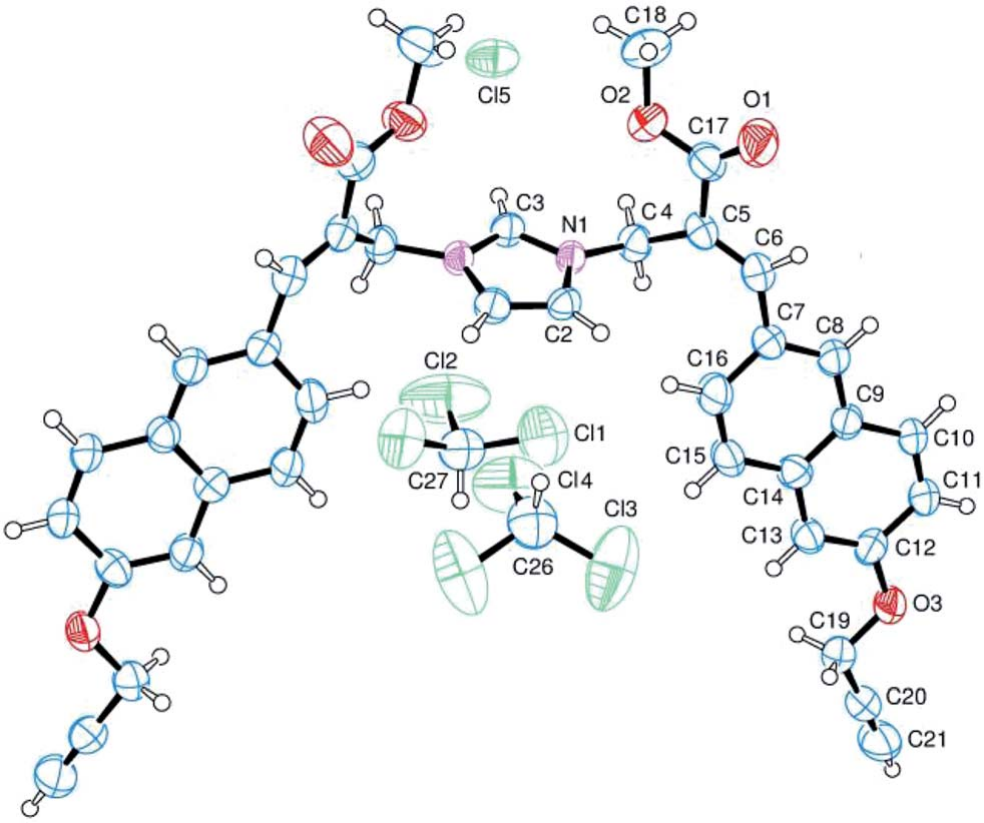
Structure of imidazolium salt 3a obtained by crystallography. The ellipsoids enclose 50% probability.

The analysis of the crystallographic structure of imidazolium salt 3a (Fig. 2) suggested that aromatic specific (*i.e.* π-stacking) interactions play an important role in stabilizing the solid state of this imidazole biadduct.

These results were confirmed with MBHA derivative 1b. In fact, imidazole derivative 2b was obtained in 92% yield by using 6 equivalents of imidazole in acetonitrile–PBS at room temperature for 24 h, whereas imidazolium salt 3b was isolated in 62% yield when 1.5 equivalents of imidazole were used in the same reaction conditions.

**Scheme 4:**
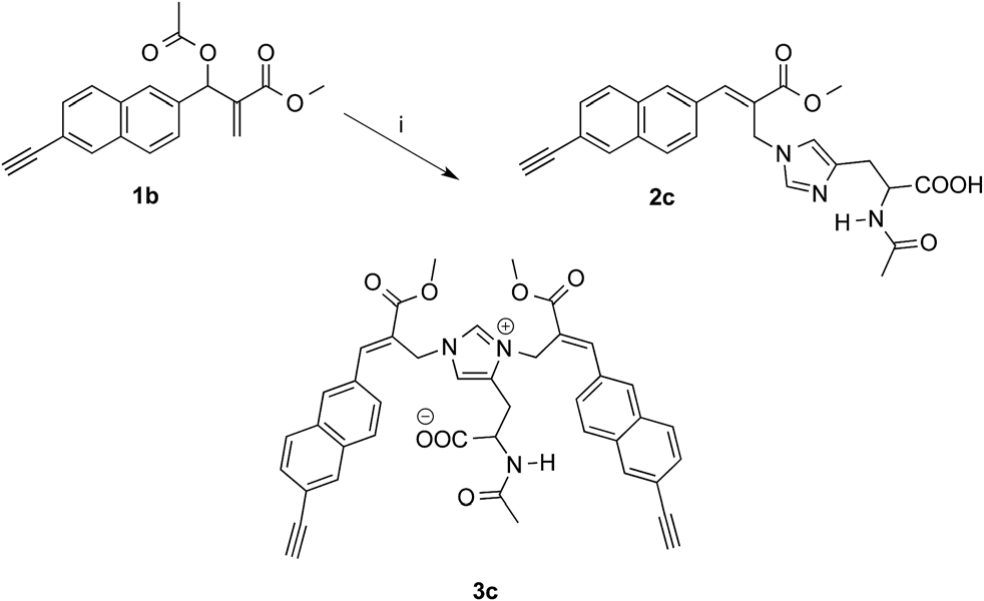
Reaction of MBHA derivative 1b with *N*-acetylhistidine. Reagents: (i) *N*-acetylhistidine, CH_3_CN, PBS.

On the other hand, the reaction of 1b with 1.5 equivalents of *N*-acetylhistidine^21^ in acetonitrile–PBS at room temperature was rather slow and required heating to be carried out in a reasonable time. Thus, 1b reacted with 1.5 equivalents of *N*-acetylhistidine in acetonitrile–PBS at the refluxing temperature to give biadduct 3c as the main product (*i.e.* yields around 80%) along with small amounts (*i.e.* yields around 5%) of 2c (Scheme 4).

In the aim of evaluating the reactivity of MBHA derivatives 1a,b in the absence of organic solvents, the structures of these compounds were manipulated by introducing solubilizing groups in the form of oligo(ethylene glycol) (OEG) chains capable of solubilizing the corresponding water-soluble derivatives in PBS.

**Scheme 5:**
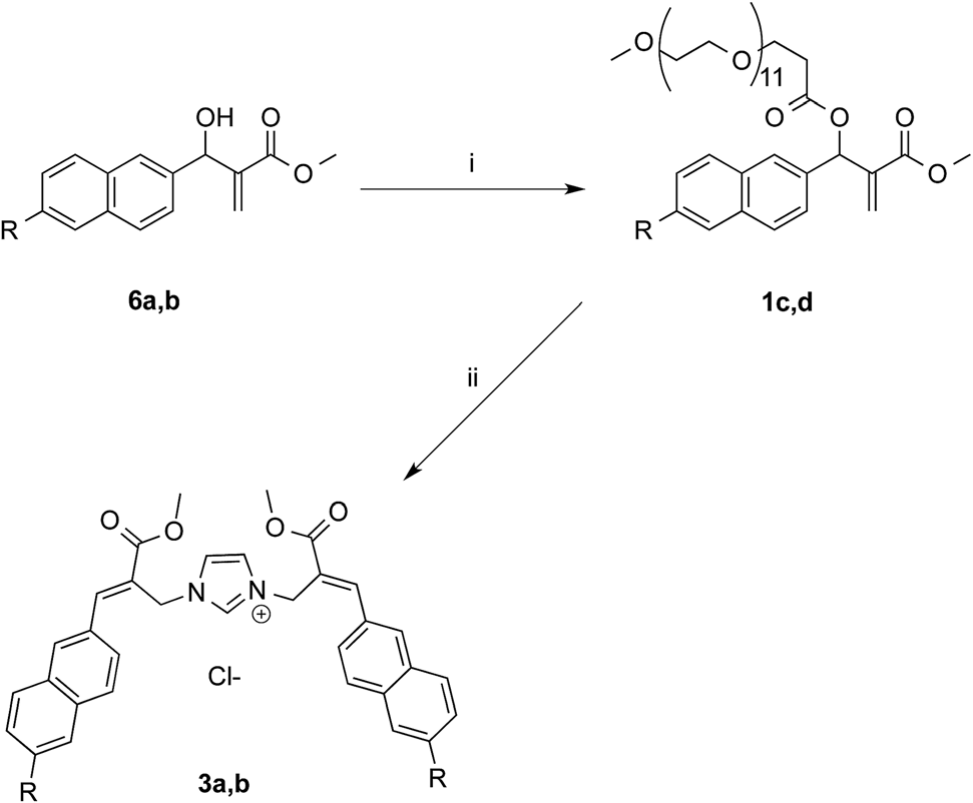
Synthesis of water-soluble MBHA derivatives 1c,d and their reactivity towards imidazole. Reagents: (i) CH_3_O(CH_2_CH_2_O)_11_CH_2_CH_2_COCl, TEA, CH_2_Cl_2_; (ii) imidazole, PBS. Substituents: R = OCH_2_CCH (1c, 3a, 6a); CCH (1d, 3b, 6b).

The first approach to the water-soluble derivatives of compounds 1a,b was based on the use of a solubilizing leaving group containing the undeca(ethylene glycol) side chain as in compounds 1c,d (Scheme 5). These latter MBHA derivatives were synthesized by acylation of 6a,b with commercially available *O*-(2-carboxyethyl)-*O*′-methyl-undeca(ethylene glycol) *via* the corresponding acyl chloride.

The reactivity of PEGylated derivatives 1c,d was firstly tested with imidazole in PBS at room temperature. The experiments performed with 1.5 equivalents of imidazole showed the almost exclusive formation of imidazolium salts 3a,b and a slightly higher reactivity of acetylene derivative 1d with respect to propargyloxy derivative 1c.

**Scheme 6:**
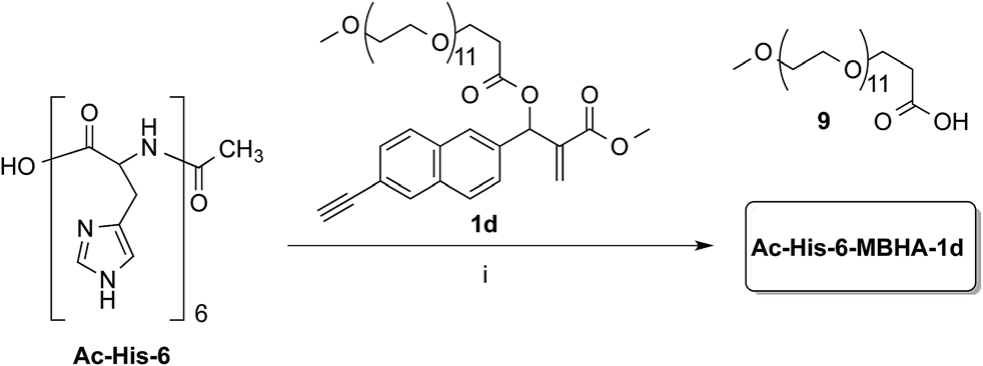
Coupling of water-soluble MBHA derivative 1d with Ac-His-6. Reagents: (i) PBS, D_2_O.

Owing to the apparently higher reactivity of water soluble 1d, this compound was selected to be used in the preliminary coupling experiments with Ac-His-6 (Scheme 6).

The experiments were performed in vials containing a PBS medium prepared with deuterium oxide and solid PBS (from Aldrich) in order to follow the reaction trend by NMR spectroscopy by integrating the signals attributed to the methylene group in position 2 of the *O*-(2-carboxyethyl)-*O*′-methyl-undeca(ethylene glycol) 9, which was produced by the concerted addition–elimination process. A ten-fold excess of compound 1d was used in the aim of saturating all the possible reactive sites in Ac-His-6 molecules. Initially, the reaction mixtures showed the appearance of clear solutions from which a white precipitate separated in the time course suggesting that an insoluble material was formed. NMR analysis of the reaction mixture at regular time intervals showed that after stirring at room temperature for 24 h Ac-His-6 was almost completely converted, whereas the conversion of 1d was only partial (around 30% of the initial amount). After 48 h at the same temperature, Ac-His-6 was completely converted and the conversion of 1d reached *ca.* 50%. After one week of stirring at room temperature the conversion of 1d reached 70% that became *ca.* 85% after three weeks. The polymeric material Ac-His-6-MBHA-1d was extracted with chloroform, purified by flash chromatography, and characterized by mass spectrometry (MS) and NMR spectroscopy techniques.

**Fig. 3:**
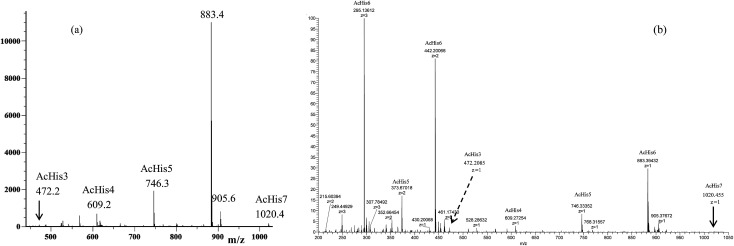
(a) MALDI-TOF mass spectrum (positive-ion mode) and (b) ESI mass spectrum (positive-ion mode) of the starting Ac-His-6 sample.

The MS studies began with the characterization of the starting Ac-His-6^22^ sample by MALDI-TOF and ESI-TOF in positive-ion mode, and the corresponding spectra are displayed in Fig. 3.

The MALDI-TOF mass spectrum shown in Fig. 3a was recorded using α-cyano-4-hydroxycinnamic acid as a matrix, and very similar mass spectra were also obtained by using 2-(4-hydroxyphenylazo)benzoic acid or 2,5-dihydroxybenzoic acid matrices. The mass spectra confirmed that the starting Ac-His-6 sample was constituted of a high percentage (*i.e.* 80%) of *N*-acetylhexahistidine (*m*/*z* 883.4). However, both mass spectra revealed also the presence of *N*-acetylheptahistidine (Ac-His-7, *m*/*z* 1020.4), *N*-acetylpentahistidine (Ac-His-5, *m*/*z* 746.3), *N*-acetyltetrahistidine (Ac-His-4, *m*/*z* 609.2), and *N*-acetyltrihistidine (Ac-His-3, *m*/*z* 472.2) molecules. On the base of the relative intensity of the corresponding peaks present in the MALDI mass spectrum, we calculated the molar composition of the starting Ac-His-6 sample (*i.e.*Ac-His-7: 1.5%; Ac-His-6: 80%; Ac-His-5: 13%; Ac-His-4: 4.5%; Ac-His-7: 1%). The ESI mass spectrum (Fig. 3b) confirmed the composition of the starting Ac-His-6 sample.

**Fig. 4:**
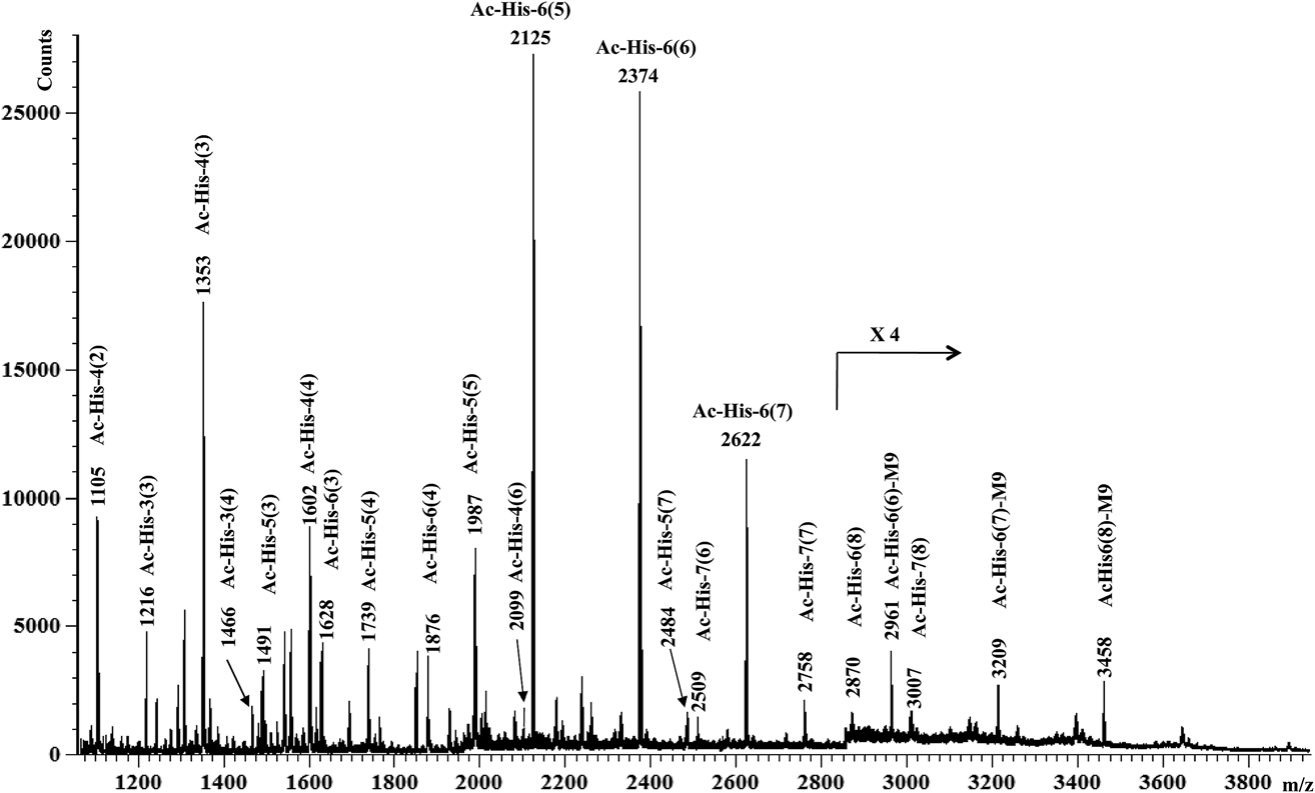
MALDI-TOF mass spectrum (positive-ion mode) of Ac-His-6-MBHA-1d obtained by reaction of Ac-His-6 with a ten-fold excess of 1d. In each assignment, the value in parentheses is the number of naphthalene substituents for each Ac-His molecule. M9 denotes the peaks assigned to the highly substituted species bearing compound 9 as a counterion of the imidazolium salt moieties.

MALDI-TOF-MS measurements were then performed on Ac-His-6-MBHA-1d material and the results (Fig. 4) were compared with those obtained with the starting Ac-His-6 sample.

This comparison suggested the existence of a considerable variability in the grafting degree (*i.e.* the number of the imidazole residues of each Ac-His-6 molecule functionalized by means of the concerted addition–elimination processes with naphthalene substituents). In particular, the peaks at *m*/*z* 2125, 2374, 2622, 2870 were assigned to Ac-His-6 molecules bearing 5, 6, 7, and 8 naphthalene substituents, respectively. Furthermore, the peaks at *m*/*z* 2622 and 2870 indicated, unequivocally, that at two naphthalene substituents were linked to the same imidazole ring belonging to one His unit of Ac-His-6 molecule, producing imidazolium salt (biadduct) moieties. The formation of biadduct residues was also confirmed by the mass peaks at *m*/*z* 2099 and 2484 corresponding to the Ac-His-4 (6) and Ac-His-5 (7), respectively. The mass peaks at *m*/*z* 2961, 3209, and 3461 were attributed to the interaction of imidazolium moieties with the anion of compound 9 (Scheme 6), which acted as a counterion and was referred as M9 in the labels reported in Fig. 4. The presence of compound 9 was also confirmed by electrospray ionization mass spectrometry (ESI-MS) analysis in negative mode, since the mass spectrum of Ac-His-6-MBHA-1d sample (Fig. ESI-1, see ESI†) showed an intense peak at 587.3 Da corresponding to molecular mass of the anion.

Interestingly, the most represented macromolecular species appeared to show a grafting degree in the range of 5–7.

**Fig. 5:**
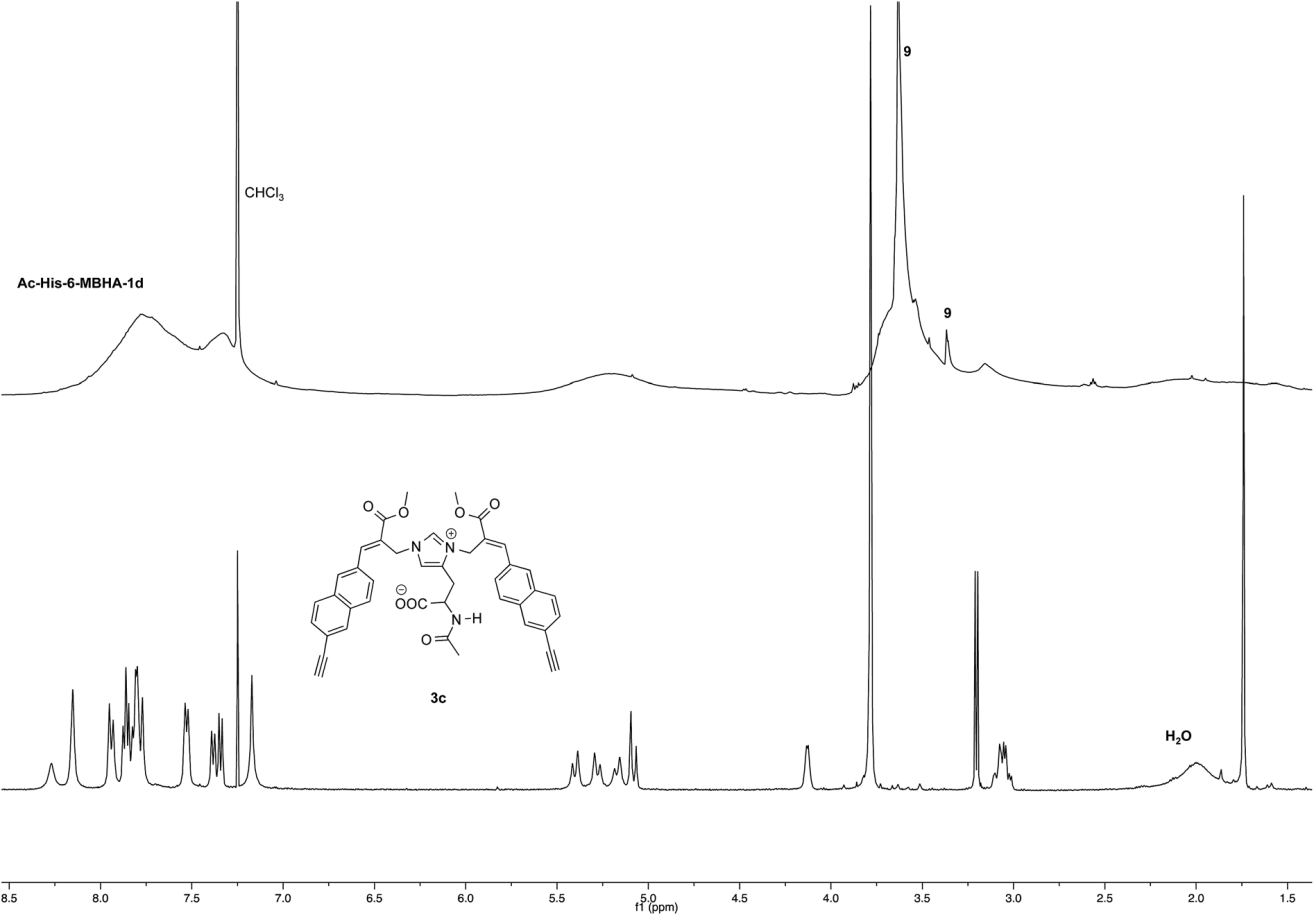
^1^H NMR spectrum (500 MHz, CDCl_3_) of Ac-His-6-MBHA-1d obtained by reaction of Ac-His-6 with a ten-fold excess of 1d compared with that of biadduct 3c. The peaks marked by 9 were attributed to compound 9, which was produced in the concerted addition–elimination process and remained as a counterion of the imidazolium salt moieties.

NMR spectroscopic studies confirmed the polymeric nature of Ac-His-6-MBHA-1d. In fact, the ^1^H NMR spectrum (Fig. 5) showed the presence of very broad signals, which were analyzed in comparison with the spectrum of biadduct 3c, which was taken into consideration as a model of the monomeric unity potentially represented into Ac-His-6-MBHA-1d structure.

**Fig. 6:**
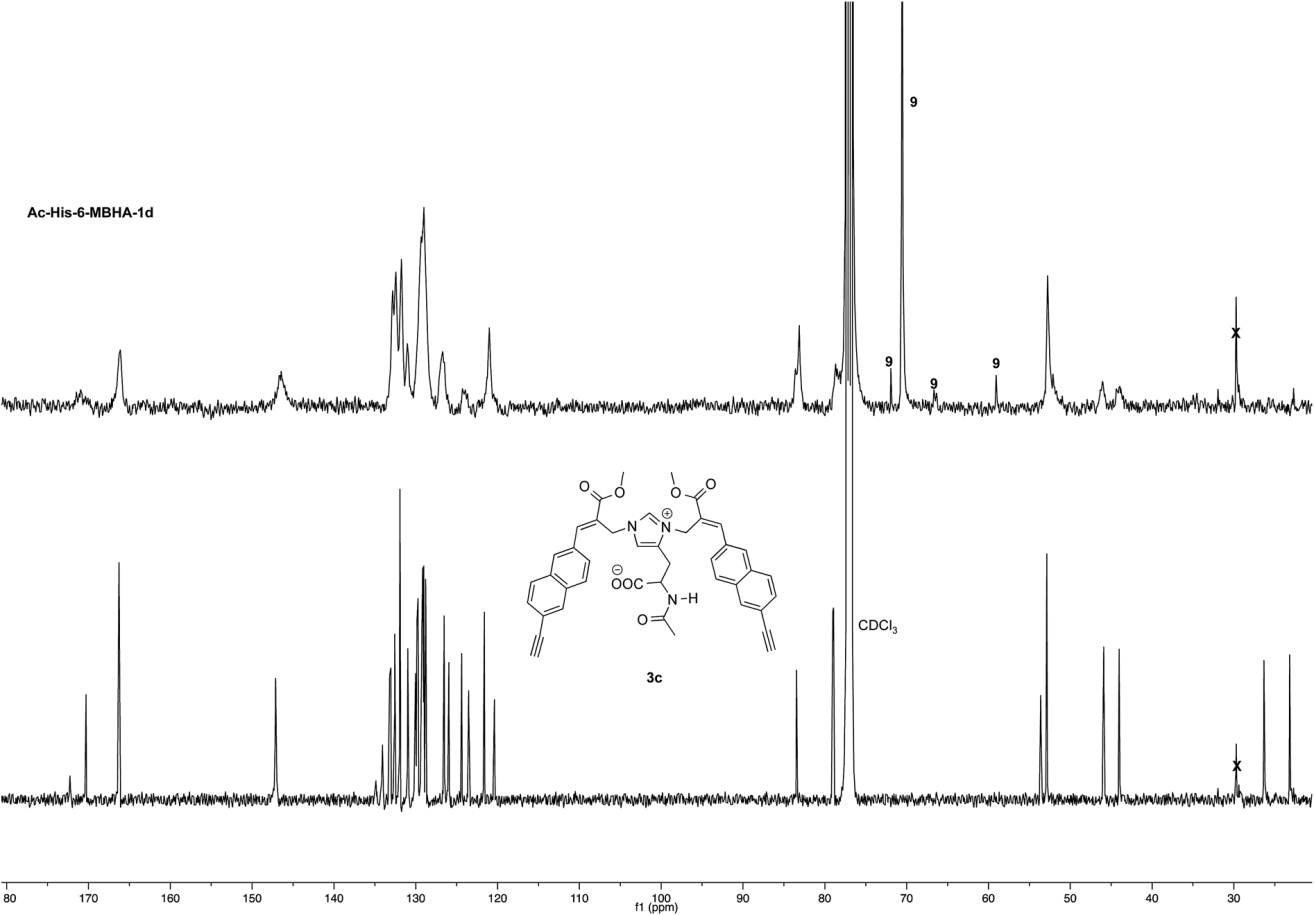
^13^C NMR spectrum (125, CDCl_3_) of Ac-His-6-MBHA-1d obtained by reaction of Ac-His-6 with a ten-fold excess of 1d compared with that of biadduct 3c. The peaks marked by 9 were attributed to compound 9, which was produced in the concerted addition–elimination process and remained as a counterion of the imidazolium salt moieties.

Very interestingly, the comparison of the ^1^H NMR spectrum of the polymeric material with that of biadduct 3c confirmed the presence of a considerable amount of biadduct residues. In fact, a close correspondence appeared to exist between the sharp signals of 3c spectrum and the broad signals in the spectrum of polymeric material of Ac-His-6-MBHA-1d. This apparent correspondence was even more evident in the comparison of ^13^C NMR spectra shown in Fig. 6.

As expected, the higher resolution of the ^13^C NMR spectrum of Ac-His-6-MBHA-1d allowed a more profitable signal-to-signal comparison to be made. In particular in the spectrum of biadduct 3c, the signals at 44.0 and 45.9 ppm attributed to the methylene bridges directly bound to the imidazole moieties of histidine appeared in Ac-His-6-MBHA-1d spectrum as two slightly broader peaks showing very similar chemical shift values (*i.e.* 44.1 and 46.0 ppm). This correspondence strongly supported the large preponderance of biadduct residues with respect to monoadduct ones. More in general, the close correspondence between the ^13^C NMR spectrum of Ac-His-6-MBHA-1d with the corresponding one of biadduct 3c confirmed this latter compound as the model of the main monomeric unit of Ac-His-6-MBHA-1d.

**Scheme 7:**
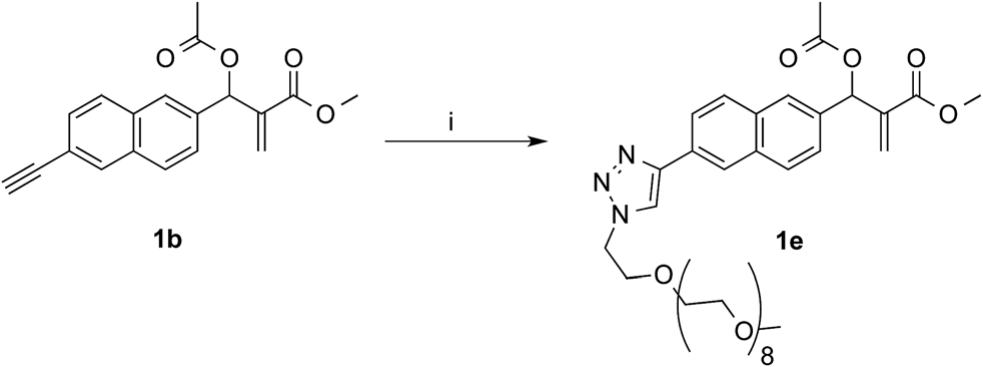
Synthesis of water-soluble MBHA derivative 1e. Reagents: (i) CH_3_(OCH_2_CH_2_)_9_N_3_, CuSO_4_, sodium ascorbate, *tert*-butanol, PBS.

In the aim of investigate on the role of OEG side chain in the reactivity of these MBHA derivatives, the position of the solubilizing moiety was shifted from the leaving group in the close proximity of the reactive center to the naphthalene ring as in compound 1e (Scheme 7). This structural modification was assumed to have a considerable impact in the reactivity of MBHA derivative 1e. Moreover, the solubilizing group remained covalently attached to resulting adduct after the concerted addition–elimination process.

**Scheme 8:**
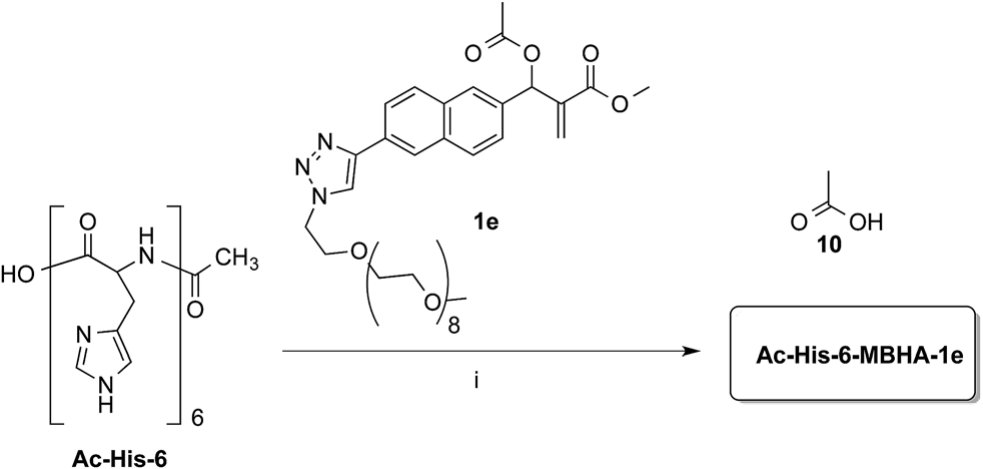
Coupling of water-soluble MBHA derivative 1e with Ac-His-6. Reagents: (i) PBS, D_2_O.

Compound 1b was reacted with *O*-(2-azidoethyl)-*O*′-methyl-octa(ethylene glycol) in the conditions of copper(i)-catalyzed azide–alkyne cycloaddition (CuAAC) in *tert*-butanol–PBS to afford triazole derivative 1e, which was found to be sufficiently soluble and stable in PBS to be used in the coupling experiments with Ac-His-6 (Scheme 8).

Experiments with a ten-fold excess of compound 1e were carried out in order to saturate all the possible reactive sites in Ac-His-6 molecules with PEGylated fluorogens and the reaction progress was monitored by NMR spectroscopy.

**Fig. 7:**
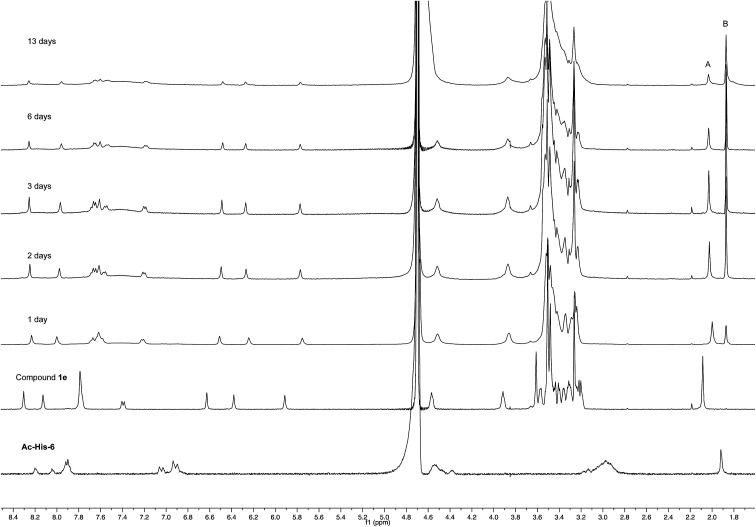
^1^H NMR spectra (400 MHz, PBS-D_2_O) of the mixtures obtained by reaction of Ac-His-6 with a ten-fold excess of compound 1e. The peaks marked with A (attributed to the acetyl moiety of 1e) and B (attributed to acetate 10 produced by the concerted addition–elimination process) were used to follow the reaction progress.

After 24 h at room temperature, the ^1^H NMR experiments (Fig. 7) demonstrated that 1e was partially (about 40%) converted, while Ac-His-6 was completely converted into a soluble polymeric material.

**Fig. 8:**
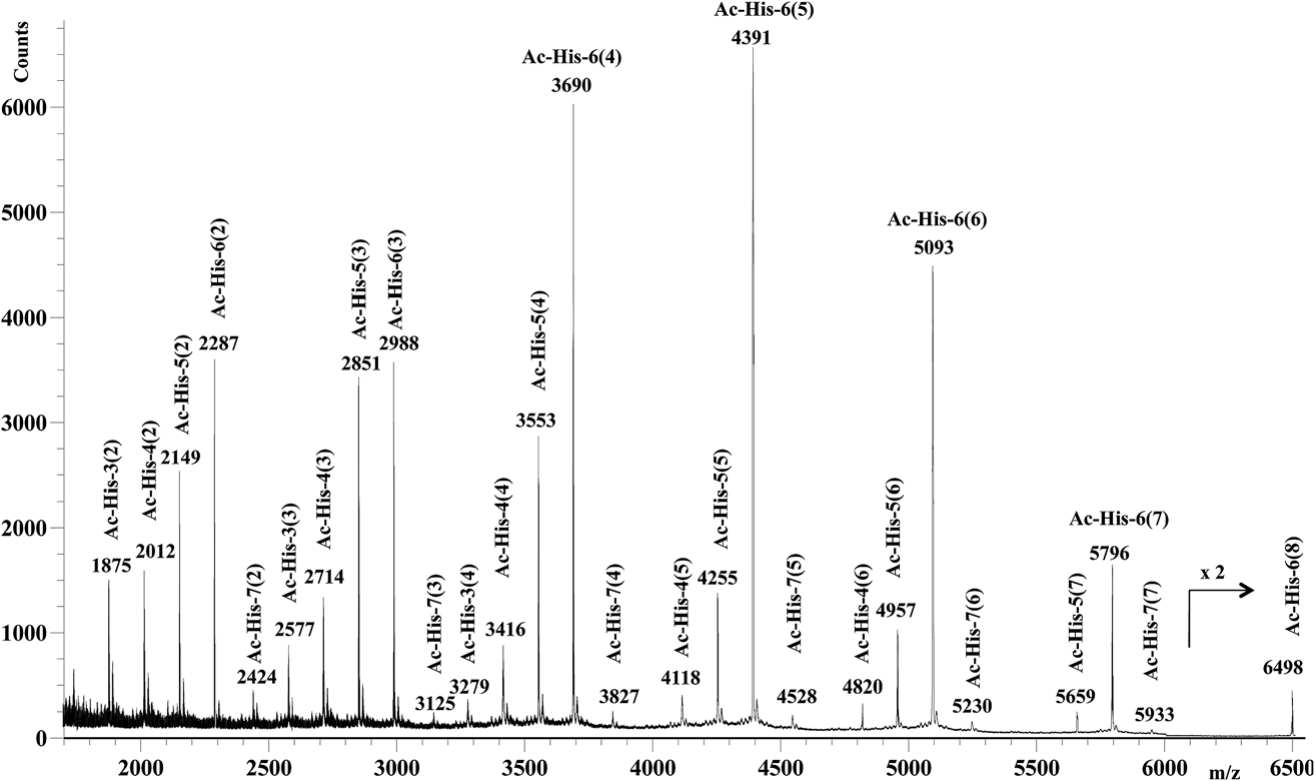
MALDI-TOF mass spectrum (positive-ion mode) of the polymeric material Ac-His-6-MBHA-1e obtained by reaction of Ac-His-6 with a ten-fold excess of compound 1e. The polymerization degree of the corresponding Ac-His molecule is reported for each monoisotopic mass peak along with the grafting degree (*i.e.* the number of naphthalene substituents for each Ac-His molecule) noted in parentheses.

After 48 h (two days) at room temperature, the conversion reached about 60% suggesting that each histidine residue could be attacked by 1e. After one-two weeks, the reaction reached a plateau characterized by around 70% conversion of 1e. These results suggested that when the solubilizing OEG chain was shifted from the leaving group in the close proximity of the reactive center (as in 1d) to the naphthalene tail (as in 1e) the reactivity increased, but the persistence of the OEG side chain attached to Ac-His-6 backbone produced by the reaction of 1e decreased the number of the sites available for functionalization. In fact, Ac-His-6 appeared to tolerate a mean of about 6–7 electrophilic attack events by compound 1e, as confirmed by the mass spectrum of polymeric material Ac-His-6-MBHA-1e (Fig. 8).

Fig. 8 shows the mass spectrum recorded in positive ion mode using 2,5-dihydroxybenzoic acid as a matrix, but very similar mass spectra were also obtained using 2-(4-hydroxyphenylazo)benzoic acid or α-cyano-4-hydroxycinnamic acid as the matrix. Similarly to the results obtained with Ac-His-6-MBHA-1d, the peaks at *m*/*z* 4820 [Ac-His-4 (6)], 5659 [Ac-His-5 (7)], and 6498 [Ac-His-6 (8)] supported the formation of biadducts (imidazolium moieties) since two naphthalene substituents derived from reagent 1e (Scheme 8) were indubitably linked to the same imidazole ring belonging to the relevant Ac-His molecule.

**Fig. 9:**
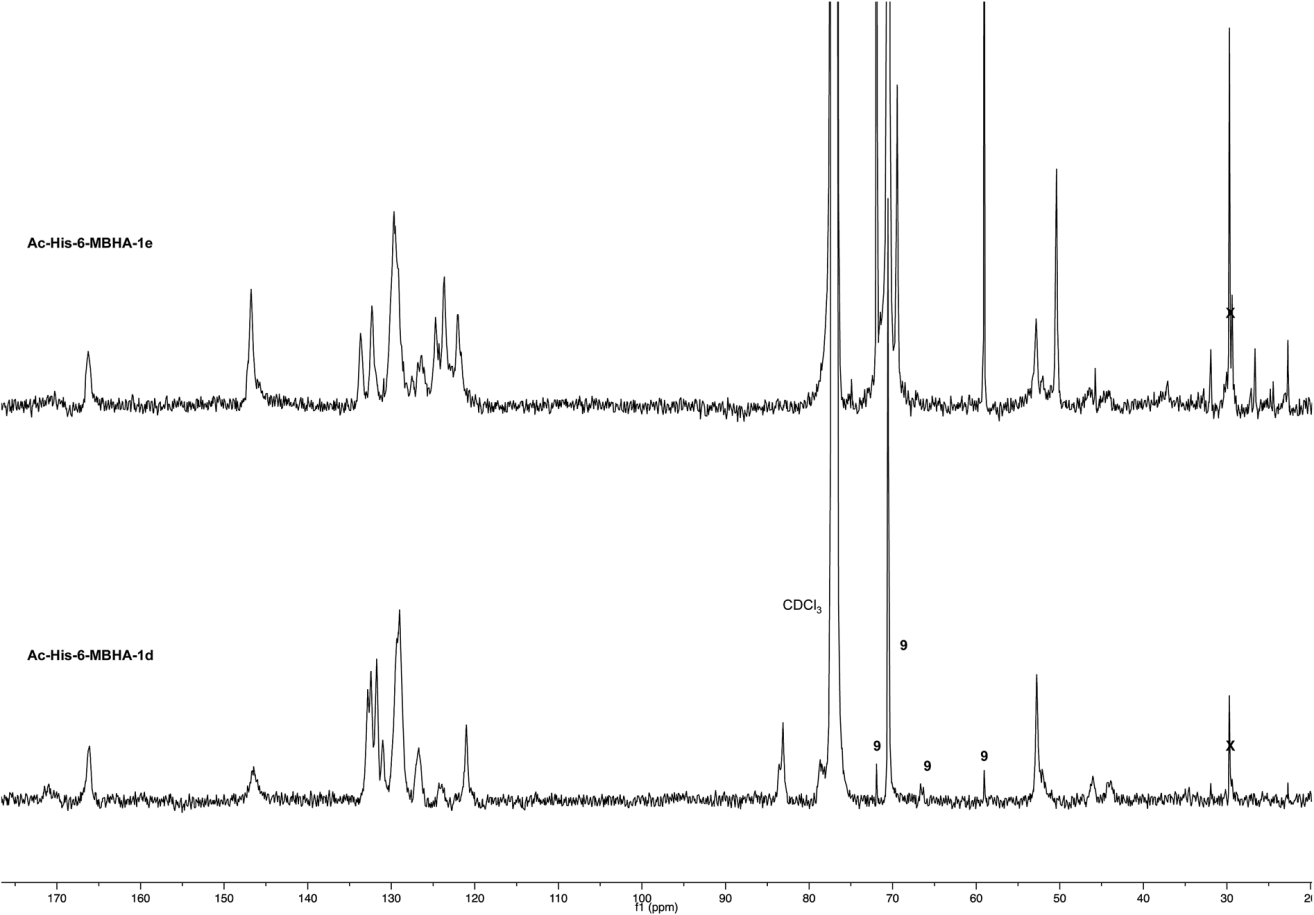
^13^C NMR spectrum (CDCl_3_) of Ac-His-6-MBHA-1e obtained by reaction of Ac-His-6 with a ten-fold excess of 1e compared with that of Ac-His-6-MBHA-1d obtained by reaction of Ac-His-6 with a ten-fold excess of 1d. The peaks marked by 9 were attributed to compound 9, which was produced in the concerted addition–elimination process and remained as a counterion of the imidazolium salt moieties.

The comparison of ^13^C NMR spectrum of Ac-His-6-MBHA-1e with the corresponding one obtained with Ac-His-6-MBHA-1d (Fig. 9) confirmed a similar reactivity in spite of the difference in the position of the solubilizing moiety. In fact, the diagnostic peaks attributed to the methylene bridges directly bound to the imidazole moieties of Ac-His-6 backbone showed roughly the same chemical shift values in Ac-His-6-MBHA-1e spectrum (44.5 and 46.6) as Ac-His-6-MBHA-1d spectrum (44.1 and 46.0 ppm). Thus, the NMR spectroscopic studies supported the preponderance of biadducts in Ac-His-6-MBHA-1e as well as in Ac-His-6-MBHA-1d.

**Fig. 10:**
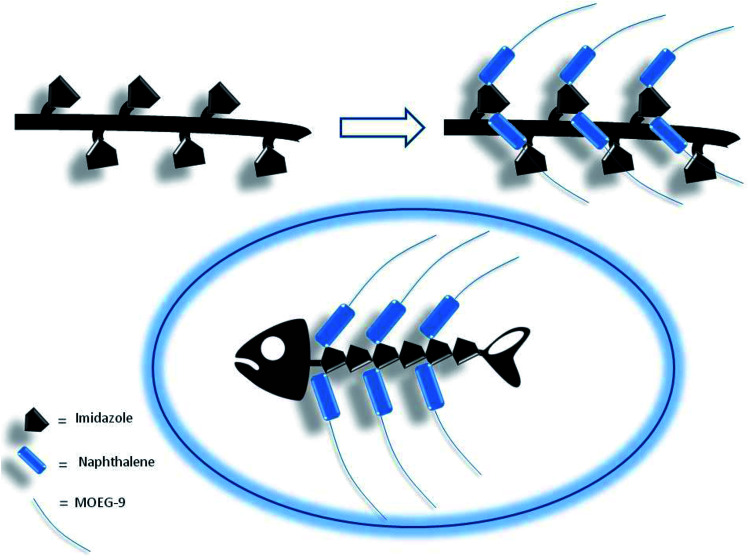
Fishbone-like architecture assumed for some organized sequences of poly-histidine derivatives grafted with naphthalene derivatives.

Thus, if the first electrophilic attack was assumed to occur randomly, the subsequent attacks could be governed by the propensity to the formation of biadducts featuring the characteristics of an imidazolium salt, which could be stabilized by the amphoteric properties of the vicinal imidazole or by the presence of suitable anions. This mechanism led to conceive the existence, within a heterogeneous polymeric population deriving from the randomness of the first attack, of molecular entities showing a more organized structure characterized by the presence of biadduct sequences as in a fishbone-like architecture (Fig. 10). However, the presence of this architecture in longer poly-histidine derivatives could be affected by their conformational preferences and their liability to form coil-like structures, which may hamper the formation and/or decrease the stability of biadduct residues. This could be particularly relevant in compound 1e because the additional steric bulk of the OEG side chain.

These results may have intriguing implications in the application of MBHA in different fields of covalent poly-histidine modification/conjugation such as *in vivo* fluorogenic labeling, fluorescence microscopy, and protein PEGylation up to the production of smart materials and biosensors, and pave the way for the preparation of fishbone-like polymer brushes and the exploration of their properties.

### Photophysical properties

The UV-visible absorption and emission features of imidazole derivatives 2a–d were characterized both in solution (*i.e.* methanol as the solvent) and in the solid state and compared in Table 1 with those of biadducts 3a–c and polymeric materials Ac-His-6-MBHA-1d and Ac-His-6-MBHA-1e.

**Table 1:** Photophysical properties of monoadducts 2a–d, biadducts 3a–c, and polymeric materials Ac-His-6-MBHA-1d and Ac-His-6-MBHA-1e

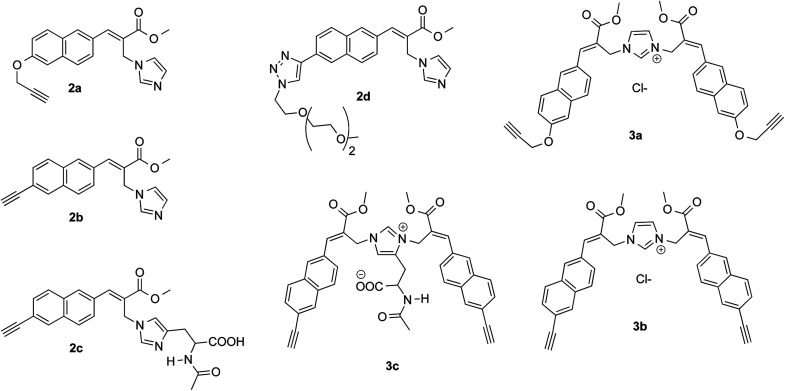					
Compd	Solution^a^			Solid^b^	
	*λ*_ab_ (nm)	*λ*_em_ c (nm)	PLQY (%)	*λ*_em_ (nm)^d^	PLQY (%)
2a	266, 320	423	0.7	428	15
2b	263, 315	404	4.4	454	15
2c	264, 318	406	0.3	438	11
2d^e^	260, 318	430	1.1	465	23
3a	270, 325	437	0.8	465	12
3b	260, 318	420	1.1	490	10
3c	265, 320	420	1.0	520	12
Ac-His-6-MBHA-1d	265, 319	425	1.5	570^f^	3
Ac-His-6-MBHA-1e	260, 329	470	0.2	570^f^	7

a Methanol.

b Powders.

c *λ*_exc_ = 320 nm.

d *λ*_exc_ = 340 nm.

e Compound 2d was synthesized as a model compound from 2b as described in the Experimental section.

f *λ*_exc_ = 460 nm.

**Fig. 11:**
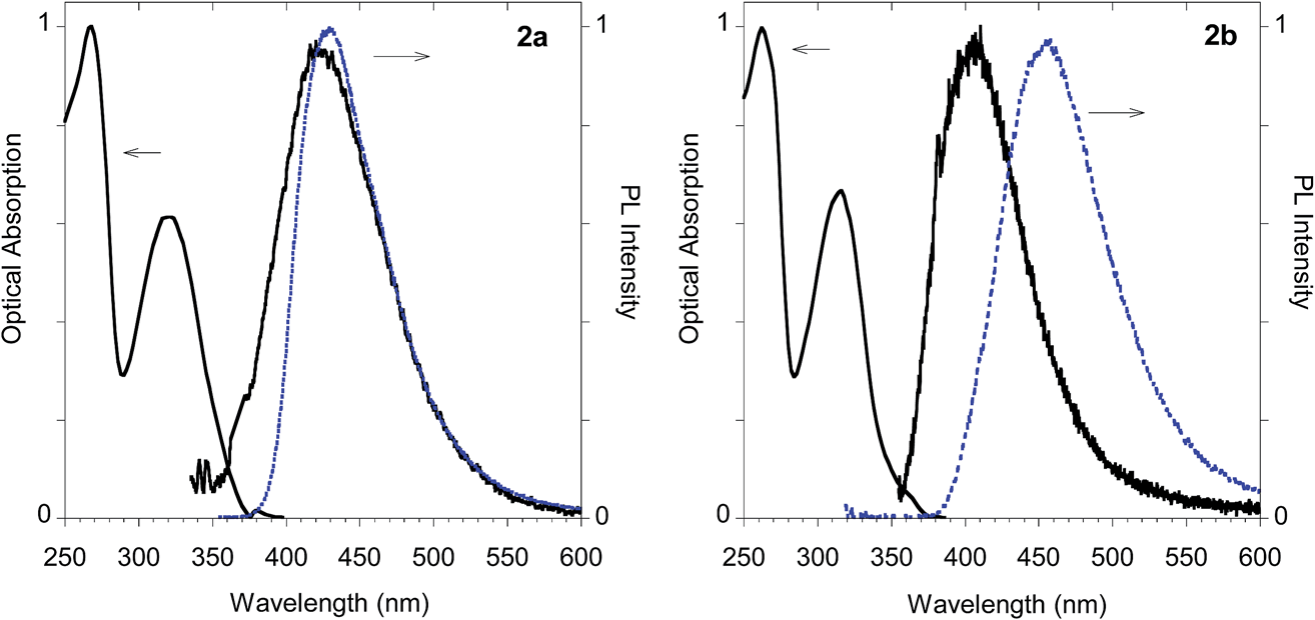
Optical properties of imidazole derivative 2a (left) and 2b (right). Normalized absorption and emission spectra obtained in methanol (black solid line) and emission spectrum of the powder (dashed blue line).

**Fig. 12:**
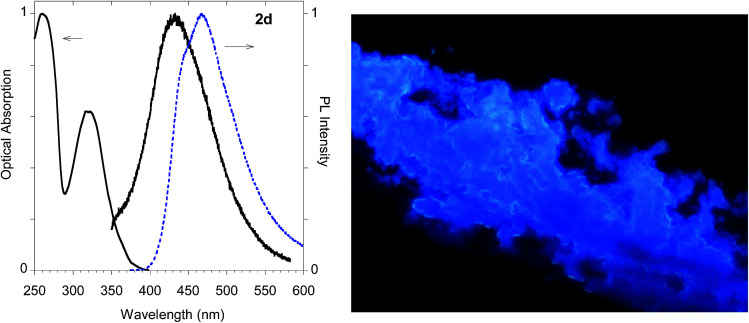
Optical properties of imidazole derivative 2d. Left panel: normalized absorption and emission spectra obtained in methanol (black solid line) and emission spectrum of the powder (dashed blue line). Right panel: fluorescence microscopy image (290 μm width) obtained with solid 2d.

**Fig. 13:**
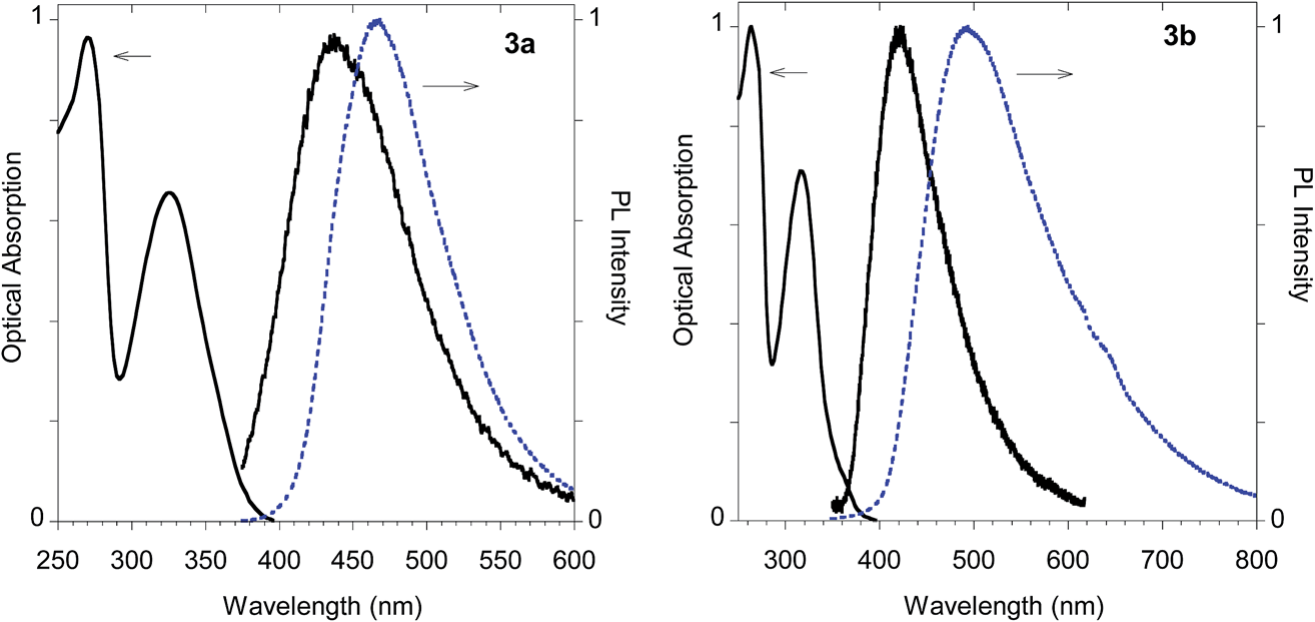
Optical features of imidazolium salts 3a and 3b. Normalized absorption and emission spectra obtained in methanol (black solid line) and emission spectrum of the powder (dashed blue line).

**Fig. 14:**
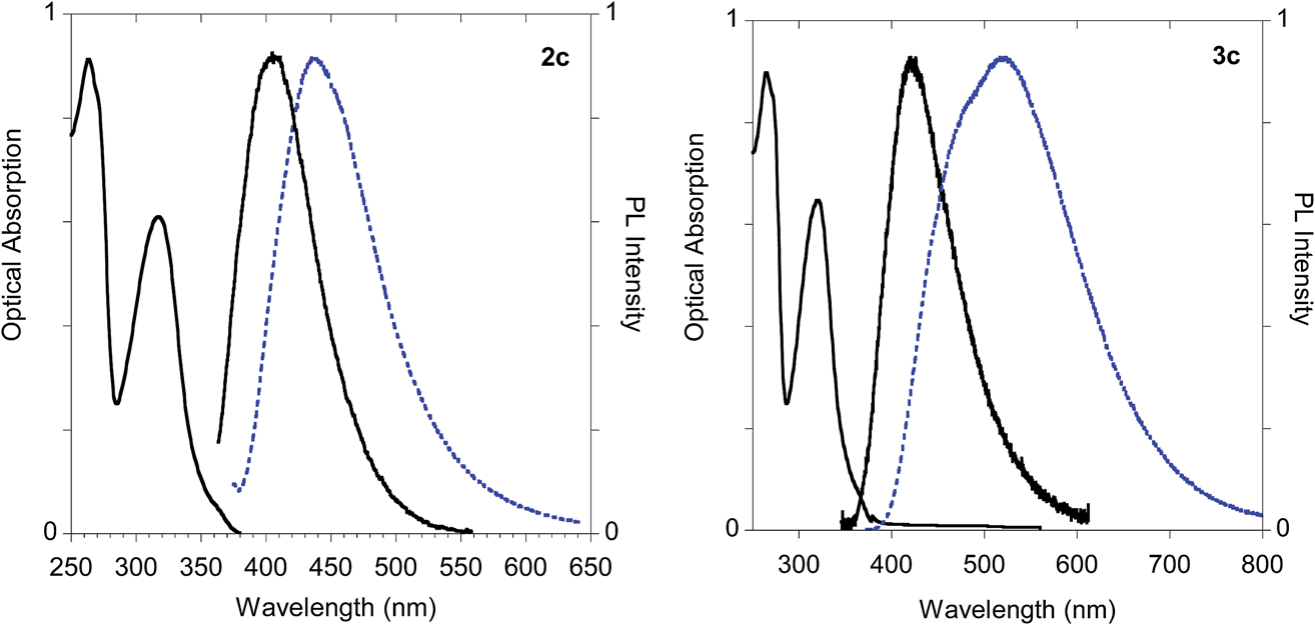
Optical features of histidine derivatives 2c and 3c. Normalized absorption and emission spectra obtained in methanol (black solid line) and emission spectrum of the powder (dashed blue line).

As shown in Table 1 all the naphthalene-based push–pull structures in solution display absorption bands at about 320 nm and 260 nm, with a quite weak photoluminescence (PL). A significant increase in PL quantum yield (QY) of the solution was obtained by transforming the propargyloxy group of 2a into the acetylene one of 2b. Interestingly, for all the compounds the low PLQY displayed by the solutions increased in the solid state with a parallel red shift, as it generally occurs in a typical AIE phenomenon.^23^ (see Fig. 11–14).

The highest PLQY value of 23% in the solid state is obtained by transformation of the acetylene moiety of 2b into the triazole moiety of the model compound 2d. In fact, powders of the triazole derivative 2d showed a sharp emission switch on in the solid state, with bright blue emissive powders, as shown in Fig. 12.

The imidazolium salts 3a and 3b conserved the interesting emission features and the AIE behavior shown by parent compounds 2a and 2b (Fig. 13).

On the other hand, significant discrepancies were observed in the couple of histidine derivatives 2c and 3c (Fig. 14).

**Fig. 15:**
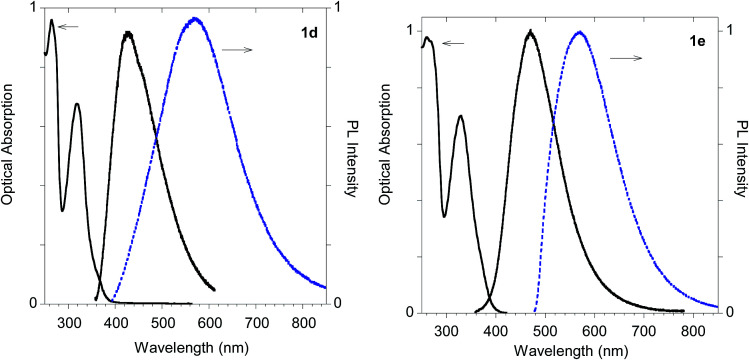
Optical features of polymeric materials Ac-His-6-MBHA-1d and Ac-His-6-MBHA-1e. Normalized absorption and emission spectra obtained in methanol (black solid line) and emission spectrum of the powder (dashed blue line).

In fact, monoadduct 2c showed a particularly low PLQY value (*i.e.* 0.3%) in solution and a moderate (*i.e.* 32 nm) red shift in the solid-state emission, whereas the corresponding biadduct 3c showed emission features in the solution aligned with those obtained with the other compounds and a peculiar (*i.e.* 100 nm) red shift in the solid state emission up to 520 nm. Interestingly, a similar red shift was observed in the polymeric materials Ac-His-6-MBHA-1d and Ac-His-6-MBHA-1e (Fig. 15), the latter showed excitation dependent emissions, suggesting the existence of different emitting species (see ESI†). These results confirm the assumption on the presence of biadduct sequences in the polymeric materials Ac-His-6-MBHA-1d and Ac-His-6-MBHA-1e as in a fishbone-like architecture.

## Conclusions

In order to explore the synthesis, the structure, and the properties of graft copolymers of poly-histidine with fluorescent molecules, a small series of Morita–Baylis–Hillman adduct (MBHA) derivatives was synthesized, and made to react with imidazole in order to evaluate their reactivity and the photophysical properties of the corresponding imidazole derivatives. Intriguingly, the reaction of MBHA derivatives 1a,b with 1.5 equivalents of imidazole in acetonitrile–phosphate buffered saline (PBS) as the reaction medium gave, along the expected mono-adducts 2a,b, the biadducts featuring the characteristics of imidazolium salt derivatives 3a,b as the main reaction products. These results were confirmed by experiments performed with *N*-acetylhistidine and 1b and suggest the possible occurrence of these structures in the products of poly-histidine labeling with MBHA derivatives 1a,b. These compounds were then transformed into the corresponding water-soluble derivatives 1c–e by introducing solubilizing groups in the form of oligo(ethylene glycol) chains in the leaving group in the close proximity of the reactive center (as in compounds 1c,d) or attached to the naphthalene moiety, far from the reactive center as in compound 1e. The solubility of these PEGylated MBHA derivatives in water allowed their reactivity to be evaluated in PBS, and, after some preliminary experiments with imidazole, compounds 1d,e were coupled with Ac-His-6. The coupling experiments were performed with ten-fold overloads of compounds 1d,e in the aim of saturating all the possible reactive sites of Ac-His-6 molecules in the polymeric materials Ac-His-6-MBHA-1d and Ac-His-6-MBHA-1e. The monitorization of the reaction progress by NMR spectroscopy suggested a higher reactivity of 1e with respect to the 1d probably caused by the steric bulk of the solubilizing OEG moiety near the reactive center of 1d. However, the most reactive 1e was observed to produce a lower grafting degree with respect to 1d, probably caused by the persistence of the solubilizing OEG chains attached to Ac-His-6 backbone of Ac-His-6-MBHA-1e. The structure of the polymeric materials Ac-His-6-MBHA-1d and Ac-His-6-MBHA-1e was investigated by mass spectrometry and NMR spectroscopy studies, which suggested the presence of biadduct residues in both the polymeric materials. These results pave the way for the preparation of fishbone-like polymer brushes, the characterization of their properties, and the exploration of their potential applications in different fields of the science such as *in vivo* fluorogenic labeling, fluorescence microscopy, protein PEGylation, up to the production of smart materials and biosensors. For example, the PEGylation of engineered proteins bearing terminal polyhistidine sequences with compound 1e could lead to the formation of PEG umbrella at the desired sequence end potentially capable of solubilizing, protecting, and detecting the engineered protein.

The analysis of the photophysical features showed that a significant emission was observed in all the naphthalene derivatives 2a–d, and imidazolium salts 3a–c, all featuring the aggregation-induced emission (AIE) phenomenon, with photoluminescence quantum yields in the solid state sensitively higher than the corresponding values measured in solution. Interestingly, biadduct 3c showed a peculiar (*i.e.* 100 nm) red shift in the solid state emission up to 520 nm. Since a similar red shift was observed in the polymeric materials Ac-His-6-MBHA-1d and Ac-His-6-MBHA-1e, these results appeared to confirm the assumption about the presence of biadduct sequences in the polymeric materials as in a fishbone-like architecture.

## Experimental section

### Chemistry

All chemicals used were of reagent grade. Yields refer to purified products and are not optimized. Melting points were determined in open capillaries on a Gallenkamp apparatus and are uncorrected. Merck silica gel 60 (230–400 mesh) was used for column chromatography. Merck TLC plates, silica gel 60 F_254_ were used for TLC. NMR spectra were recorded by means of either a DRX 400 AVANCE or a Bruker DRX 500 AVANCE spectrometer in the indicated solvents (TMS as internal standard); the values of the chemical shifts (*δ*) are expressed in ppm and the coupling constants (*J*) in Hz. Mass spectra were recorded on an Agilent 1100 LC/MSD.

#### 6-(Prop-2-ynyloxy)-2-naphthaldehyde (5)

A mixture of 4 (0.14 g, 0.81 mmol), potassium carbonate (0.34 g, 2.4 mmol), and propargyl bromide (80 wt%, 0.27 mL, 2.4 mmol) in acetonitrile (10 mL) was heated under reflux for 3 h and then concentrated under reduced pressure. The resulting residue was partitioned between ethyl acetate and water. The organic layer was dried over sodium sulfate and concentrated under reduced pressure. Purification of the residue by flash chromatography purified by flash chromatography with petroleum ether–ethyl acetate (9 : 1) as the eluent to obtain compound 5 (0.13 g, yield 76%) as a white solid melting at 107–108 °C. ^1^H NMR (500 MHz, CDCl_3_): 2.58 (t, *J* = 2.4, 1H), 4.85 (d, *J* = 2.4, 2H), 7.26–7.30 (m, 2H), 7.83 (d, *J* = 8.5, 1H), 7.91–7.94 (m, 2H), 8.27 (s, 1H), 10.10 (s, 1H). ^13^C NMR (125 MHz, CDCl_3_): 55.9, 76.2, 77.9, 107.7, 120.0, 123.7, 128.0, 128.3, 131.3, 132.7, 134.2, 137.9, 158.0, 192.0. MS (ESI) *m*/*z*: [M + Na]^+^ calcd for C_14_H_10_O_2_Na 233.1; found 233.1.

#### 6-Ethynyl-2-naphthaldehyde (8)

To a degassed solution of 7 (100 mg, 0.425 mmol) in 6.0 mL of THF–TEA (5 : 1) containing Pd(PPh_3_)_2_Cl_2_ (21 mg, 0.0299 mmol) and CuI (6.0 mg, 0.0315 mmol) was added ethynyltrimethylsilane (90 μL, 0.64 mmol). After stirring overnight at room temperature, the reaction mixture was concentrated under reduced pressure. The residue was dissolved in dichloromethane and washed with water. The organic layer was dried over sodium sulfate and concentrated under reduced pressure. The residue was purified by flash chromatography with petroleum ether–ethyl acetate (9 : 1) as the eluent to afford 6-[(trimethylsilyl)ethynyl]-2-naphthaldehyde (88 mg, yield 82%), which was promptly used in the subsequent step. ^1^H NMR (400 MHz, CDCl_3_): 0.28 (s, 9H), 7.59 (dd, *J* = 8.4, 1.6, 1H), 7.83–7.98 (m, 3H), 8.03 (s, 1H), 8.29 (s, 1H), 10.14 (s, 1H).

A mixture of 6-[(trimethylsilyl)ethynyl]-2-naphthaldehyde (80 mg, 0.317 mmol) and potassium carbonate (4.0 mg, 0.0289 mmol) in methanol (5.0 mL) was stirred overnight at room temperature and then concentrated under reduced pressure. The resulting residue was partitioned between dichloromethane and water. The organic layer was dried over sodium sulfate and concentrated under reduced pressure. Purification of the residue by flash chromatography with petroleum ether–ethyl acetate (9 : 1) as the eluent gave compound 8 (56 mg, yield 98%) as a white solid. ^1^H NMR (500 MHz, CDCl_3_): 3.24 (s, 1H), 7.62 (dd, *J* = 8.5, 1.65, 1H), 7.89 (d, *J* = 8.5, 1H), 7.94–7.98 (m, 2H), 8.06 (s, 1H), 8.31 (s, 1H), 10.15 (s, 1H). ^13^C NMR (125 MHz, CDCl_3_): 79.3, 83.3, 122.8, 123.7, 128.9, 129.6, 129.8, 132.2, 134.0, 134.7, 135.8, 192.0. MS (ESI) *m*/*z*: [M + Na]^+^ calcd for C_13_H_8_ONa 203.1; found 203.1.

#### General procedure for the preparation of compounds 6a,b

A mixture of the appropriate aldehyde (5 or 8) in the suitable solvent containing the suitable amounts of methyl acrylate and of 1,4-diazabicyclo[2.2.2]octane (DABCO) was stirred at room temperature for 48 h in darkness, and then concentrated under reduced pressure. The resulting residue was dissolved in ethyl acetate and washed in sequence with water, 1 N HCl, and brine. The organic layer was dried over sodium sulfate and concentrated under reduced pressure. Purification of the residue by flash chromatography with the suitable eluent afforded the expected acrylate derivative (6a,b).

#### Methyl 2-[hydroxyl[6-(prop-2-ynyloxy)naphthalen-2-yl]methyl]acrylate (6a)

The title compound was prepared from 5 (0.50 g, 2.38 mmol), methanol (3.0 mL), THF (2.0 mL), methyl acrylate (3.0 mL), and DABCO (0.27 g, 2.41 mmol) by following the above general procedure and purified by flash chromatography with petroleum ether–ethyl acetate (8 : 2) as the eluent to obtain compound 6a (0.26 g, yield 37%) as a colorless oil, which crystallized on standing (mp 77–78 °C). ^1^H NMR (400 MHz, CDCl_3_): 2.54 (t, *J* = 2.4, 1H), 3.03 (d, *J* = 5.6, 1H), 3.71 (s, 3H), 4.80 (d, *J* = 2.4, 2H), 5.71 (d, *J* = 5.5, 1H), 5.87 (s, 1H), 6.36 (s, 1H), 7.18 (dd, *J* = 8.9, 2.6, 1H), 7.22 (d, *J* = 2.2, 1H), 7.44 (dd, *J* = 8.7, 1.7, 1H), 7.71–7.80 (m, 3H). ^13^C NMR (125 MHz, CDCl_3_): 52.0, 55.9, 73.4, 75.7, 78.4, 107.3, 119.0, 125.2, 125.4, 126.3, 127.3, 129.1, 129.8, 133.9, 136.8, 141.9, 155.7, 166.8. MS (ESI) *m*/*z*: [M + Na]^+^ calcd for C_18_H_16_O_4_Na 319.1; found 319.0.

#### Methyl 2-[(6-ethynylnaphthalen-2-yl) (hydroxy)methyl]acrylate (6b)

The title compound was prepared from 8 (0.54 g, 3.0 mmol), methanol (10 mL), THF (10 mL), methyl acrylate (10 mL), and DABCO (0.34 g, 3.0 mmol) by following the above general procedure and purified by flash chromatography with petroleum ether–ethyl acetate–dichloromethane (7 : 2 : 1) as the eluent to obtain compound 6b (0.49 g, yield 61%) as a pale yellow oil. ^1^H NMR (500 MHz, CDCl_3_): 3.14 (s, 1H), 3.18 (br s, 1H), 3.72 (s, 3H), 5.71 (s, 1H), 5.86 (t, *J* = 1.1, 1H), 6.38 (s, 1H), 7.48 (dd, *J* = 8.5, 1.7, 1H), 7.51 (dd, *J* = 8.4, 1.5, 1H), 7.77 (d, *J* = 8.4, 2H), 7.83 (s, 1H), 7.99 (s, 1H). ^13^C NMR (125 MHz, CDCl_3_): 52.1, 73.3, 77.6, 83.9, 119.6, 125.4, 126.7, 128.1, 128.2, 128.9, 132.0, 132.4, 132.9, 139.8, 141.6, 166.8. MS (ESI) *m*/*z*: [M + Na]^+^ calcd for C_17_H_14_O_3_Na 289.1; found 289.0.

#### General procedure for the preparation of Morita–Baylis–Hillman acetates 1a,b

To a solution of the suitable alcohol (6a,b) in dry dichloromethane containing TEA (2.5 equivalents), acetyl chloride (2.0 equivalents) was added dropwise. After stirring at room temperature for 1 h, the reaction mixture was washed with water. The organic layer was dried over sodium sulfate and concentrated under reduced pressure. The residue was purified by flash chromatography with petroleum ether–ethyl acetate (8 : 2) as the eluent to afford the corresponding Morita–Baylis–Hillman acetate (1a,b).

#### Methyl 2-[acetoxy[6-(prop-2-ynyloxy)naphthalen-2-yl]methyl]acrylate (1a)

The title compound was prepared from 6a (0.14 g, 0.47 mmol), TEA (0.20 mL, 1.4 mmol), dry dichloromethane (10 mL), and acetyl chloride (0.067 mL, 0.94 mmol) by following the above general procedure to obtain compound 1a (0.14 g, yield 89%) as a colorless oil. ^1^H NMR (500 MHz, CDCl_3_): 2.11 (s, 3H), 2.53 (t, *J* = 2.4, 1H), 3.69 (s, 3H), 4.79 (d, *J* = 2.4, 2H), 5.92 (s, 1H), 6.43 (s, 1H), 6.81 (s, 1H), 7.18 (dd, *J* = 8.9, 2.6, 1H), 7.21 (d, *J* = 2.5, 1H), 7.44 (dd, *J* = 8.5, 1.8, 1H), 7.71–7.75 (m, 2H), 7.78 (s, 1H). ^13^C NMR (125 MHz, CDCl_3_): 21.2, 52.1, 55.8, 73.3, 75.7, 78.3, 107.3, 119.2, 125.7, 125.9, 127.0, 127.3, 128.9, 129.9, 133.3, 134.2, 139.6, 155.9, 165.5, 169.5. MS (ESI) *m*/*z*: [M + Na]^+^ calcd for C_20_H_18_O_5_Na 361.1; found 361.0.

#### Methyl 2-[acetoxy(6-ethynylnaphthalen-2-yl)methyl]acrylate (1b)

The title compound was prepared from 6b (0.10 g, 0.376 mmol), TEA (0.13 mL, 0.95 mmol), dry dichloromethane (10 mL), and acetyl chloride (0.054 mL, 0.76 mmol) by following the above general procedure to obtain compound 1b (0.096 g, yield 83%) as a colorless oil, which crystallized on standing (mp 100–101 °C). ^1^H NMR (500 MHz, CDCl_3_): 2.12 (s, 3H), 3.15 (s, 1H), 3.70 (s, 3H), 5.94 (s, 1H), 6.45 (s, 1H), 6.82 (s, 1H), 7.49 (dd, *J* = 8.6, 1.7, 1H), 7.52 (dd, *J* = 8.4, 1.6, 1H), 7.77 (d, *J* = 8.4, 2H), 7.82 (s, 1H), 7.99 (s, 1H). ^13^C NMR (125 MHz, CDCl_3_): 21.1, 52.1, 73.1, 77.7, 83.8, 119.9, 125.9, 126.1, 126.9, 128.2, 128.3, 129.0, 132.0, 132.6, 132.7, 136.3, 139.3, 165.3, 169.4. MS (ESI) *m*/*z*: [M + Na]^+^ calcd for C_19_H_16_O_4_Na 331.1; found 331.0.

#### General procedure for the preparation of imidazole derivatives 2a,b (procedure A)

A mixture of the appropriate Baylis–Hillman acetate (1a,b) in THF–water (5 : 1) containing imidazole (1.5 equivalents) was heated under reflux overnight. After cooling to room temperature, the reaction mixture was diluted with brine and extracted with dichloromethane. The organic layer was dried over sodium sulfate and concentrated under reduced pressure. Purification of the residue by flash chromatography with ethyl acetate as the eluent gave the corresponding imidazole derivative (2a,b).

#### (*E*)-Methyl 2-[(1*H*-imidazol-1-yl)methyl]-3-[6-(prop-2-ynyloxy)naphthalen-2-yl]acrylate (2a)

The title compound was prepared from 1a (50 mg, 0.148 mmol), imidazole (15 mg, 0.220 mmol), THF (5.0 mL), and water (1.0 mL) by means of procedure A to obtain compound 2a (40 mg, yield 78%) as a colorless oil which crystallized on standing (mp 127–128 °C). ^1^H NMR (500 MHz, CDCl_3_): 2.56 (t, *J* = 2.4, 1H), 3.83 (s, 3H), 4.83 (d, *J* = 2.4, 2H), 5.06 (s, 2H), 6.91 (s, 1H), 7.05 (s, 1H), 7.22–7.24 (m, 2H), 7.40 (dd, *J* = 8.5, 1.5, 1H), 7.53 (s, 1H), 7.71 (s, 1H), 7.74 (d, *J* = 8.8, 1H), 7.79 (d, *J* = 8.5, 1H), 8.16 (s, 1H). ^13^C NMR (125 MHz, CDCl_3_): 43.2, 52.6, 55.9, 76.0, 78.1, 107.3, 118.8, 119.9, 126.2, 126.4, 127.8, 128.8, 129.2, 129.3, 129.5, 130.2, 134.7, 137.0, 145.2, 156.8, 167.2. MS (ESI) *m*/*z*: [M + H]^+^ calcd for C_21_H_19_N_2_O_3_ 347.1; found 347.1.

Compound 2a was also prepared by means of procedure B as it follows. A mixture of 1a (50 mg, 0.148 mmol) in acetonitrile (5.0 mL) and water (6.0 mL) containing phosphate buffered saline (PBS, pH 7.4) powder (Aldrich, 60 mg) and imidazole (60 mg, 0.89 mmol) was stirred at room temperature for 24 h, then diluted with brine, and extracted with chloroform. The organic layer was dried over sodium sulfate and concentrated under reduced pressure. The residue was purified by flash chromatography with ethyl acetate as the eluent to afford compound 2a (41 mg, yield 80%).

#### (*E*)-Methyl 2-[(1*H*-imidazol-1-yl)methyl]-3-(6-ethynylnaphthalen-2-yl)acrylate (2b)

The title compound was prepared from 1b (50 mg, 0.162 mmol), imidazole (17 mg, 0.250 mmol), THF (5.0 mL), and water (1.0 mL) by means of procedure A to obtain compound 2b (45 mg, yield 88%) as a colorless oil which crystallized on standing (mp 120–121 °C). ^1^H NMR (500 MHz, CDCl_3_): 3.20 (s, 1H), 3.84 (s, 3H), 5.03 (s, 2H), 6.89 (s, 1H), 7.05 (s, 1H), 7.42 (dd, *J* = 8.5, 1.7, 1H), 7.51 (s, 1H), 7.57 (dd, *J* = 8.5, 1.5, 1H), 7.73 (s, 1H), 7.76 (d, *J* = 8.5, 1H), 7.85 (d, *J* = 8.5, 1H), 8.02 (s, 1H), 8.16 (s, 1H). ^13^C NMR (125 MHz, CDCl_3_): 43.1, 52.7, 78.7, 83.5, 118.8, 121.1, 126.5, 127.5, 128.5, 128.7, 128.8, 129.5, 129.7, 132.0, 132.3, 132.6, 132.8, 137.0, 144.6, 166.9. MS (ESI) *m*/*z*: [M + H]^+^ calcd for C_20_H_17_N_2_O_2_ 317.1; found 317.1.

Compound 2b was also prepared by means of procedure B as it follows. A mixture of 1b (50 mg, 0.162 mmol) in acetonitrile (5.0 mL) and water (6.0 mL) containing phosphate buffered saline (PBS, pH 7.4) powder (Aldrich, 60 mg) and imidazole (66 mg, 0.97 mmol) was stirred at room temperature for 24 h, then diluted with brine, and extracted with chloroform. The organic layer was dried over sodium sulfate and concentrated under reduced pressure. The residue was purified by flash chromatography with ethyl acetate as the eluent to afford compound 2b (47 mg, yield 92%).

#### 1,3-Bis[(*E*)-2-(methoxycarbonyl)-3-[6-(prop-2-ynyloxy)naphthalen-2-yl]allyl]-1*H*-imidazol-3-ium chloride (3a)

A mixture of 1a (50 mg, 0.148 mmol) in acetonitrile (5.0 mL) and water (5.0 mL) containing phosphate buffered saline (PBS, pH 7.4) powder (Aldrich, 50 mg) and imidazole (15 mg, 0.22 mmol) was stirred at room temperature for 36 h, then diluted with brine, and extracted with chloroform. The organic layer was dried over sodium sulfate and concentrated under reduced pressure. The residue was purified by flash chromatography with ethyl acetate as the eluent to afford compound 2a (9.0 mg, yield 18%) and with chloroform–methanol (9 : 1) to obtain compound 3a as a white solid (23 mg, yield 47%). An analytical sample of compound 3a was prepared by recrystallization from chloroform–methanol (5 : 1) by slow evaporation to obtain X-ray quality colorless plates (mp 190 °C dec). ^1^H NMR (400 MHz, CDCl_3_): 2.55 (t, *J* = 2.4, 2H), 3.82 (s, 6H), 4.79 (d, *J* = 2.4, 4H), 5.58 (s, 4H), 7.00 (s, 2H), 7.18–7.23 (m, 4H), 7.46–7.51 (m, 2H), 7.82–7.93 (m, 6H), 8.20 (s, 2H), 10.86 (s, 1H). ^13^C NMR (125 MHz, CDCl_3_): 46.2, 52.9, 55.9, 76.1, 78.0, 107.3, 120.1, 121.0, 122.8, 126.5, 128.2, 128.4, 128.8, 130.2, 130.7, 135.1, 139.3, 147.5, 157.1, 166.7. MS (ESI) *m*/*z*: [M]^+^ calcd for C_39_H_33_N_2_O_6_ 625.2; found 625.2.

#### 1,3-Bis[(*E*)-3-(6-ethynylnaphthalen-2-yl)-2-(methoxycarbonyl)allyl]-1*H*-imidazol-3-ium chloride (3b)

A mixture of 1b (50 mg, 0.162 mmol) in acetonitrile (5.0 mL) and water (5.0 mL) containing phosphate buffered saline (PBS, pH 7.4) powder (Aldrich, 50 mg) and imidazole (17 mg, 0.25 mmol) was stirred at room temperature for 24 h, then diluted with brine, and extracted with chloroform. The organic layer was dried over sodium sulfate and concentrated under reduced pressure. The residue was purified by flash chromatography with ethyl acetate as the eluent to afford compound 2b (12 mg, yield 23%) and with chloroform–methanol (9 : 1) to obtain compound 3b as an off-white solid (30 mg, yield 62%). An analytical sample of compound 3b was prepared by recrystallization from chloroform–methanol (5 : 1) by slow evaporation to a pale yellow microcrystalline powder (mp 135 °C dec). ^1^H NMR (400 MHz, CDCl_3_): 3.20 (s, 2H), 3.83 (s, 6H), 5.55 (s, 4H), 6.96 (s, 2H), 7.48 (d, *J* = 8.2, 2H), 7.53 (d, *J* = 8.3, 2H), 7.86 (d, *J* = 8.4, 2H), 7.95–7.99 (m, 6H), 8.20 (s, 2H), 10.87 (s, 1H). ^13^C NMR (125 MHz, CDCl_3_): 46.1, 53.0, 78.8, 83.5, 121.1, 121.5, 124.3, 126.5, 129.0, 129.1, 129.5, 129.8, 131.0, 131.9, 132.6, 133.1, 139.4, 146.8, 166.3. MS (ESI) *m*/*z*: [M]^+^ calcd for C_37_H_29_N_2_O_4_ 565.2; found 565.2.

#### (*E*)-Methyl 2-[(1*H*-imidazol-1-yl)methyl]-3-[6-[1-[2-[2-(2-methoxyethoxy)ethoxy]ethyl]-1*H*-1,2,3-triazol-4-yl]naphthalen-2-yl]acrylate (2d)

A round bottom 10 mL flask was charged under an inert atmosphere with *tert*-butanol (2.0 mL), water (2.0 mL), phosphate buffered saline (PBS, pH 7.4) powder (Aldrich, 20 mg), and a solution of CuSO_4_·5H_2_O (12.5 mg, 0.037 mmol) in 0.50 mL of water. Then, a 1 M solution of sodium ascorbate in water (0.50 mL) was added and 1.0 mL of the resulting mixture was used as the catalyst. A mixture of 2b (50 mg, 0.158 mmol) in *tert*-butanol (8.0 mL) and water (4.0 mL) containing phosphate buffered saline (PBS, pH 7.4) powder (Aldrich, 40 mg) and 1-azido-2-[2-(2-methoxyethoxy)ethoxy]ethane (60 mg, 0.317 mmol). The reaction mixture was stirred at room temperature for 5 h, then diluted with brine, and extracted with dichloromethane. The organic layer was dried over sodium sulfate and concentrated under reduced pressure. Purification of the residue by flash chromatography with chloroform–methanol (9 : 1) as the eluent gave compound 2d (78 mg, yield 98%) as a pale yellow oil, which crystallized on standing. ^1^H NMR (500 MHz, CDCl_3_): 3.32 (s, 3H), 3.51–3.53 (m, 2H), 3.61–3.68 (m, 6H), 3.84 (s, 3H), 3.93 (t, *J* = 5.0, 2H), 4.63 (t, *J* = 4.9, 2H), 5.06 (s, 2H), 6.93 (s, 1H), 7.06 (s, 1H), 7.42 (dd, *J* = 8.5, 1.5, 1H), 7.54 (s, 1H), 7.76 (s, 1H), 7.87 (d, *J* = 8.6, 1H), 7.93 (d, *J* = 8.5, 1H), 7.99 (dd, *J* = 8.5, 1.6, 1H), 8.16 (s, 1H), 8.18 (s, 1H), 8.38 (s, 1H). ^13^C NMR (125 MHz, CDCl_3_): 43.2, 50.5, 52.6, 59.0, 69.5, 70.5, 71.9, 118.8, 121.7, 124.0, 125.1, 126.3, 126.9, 129.1, 129.4, 129.9, 131.4, 132.6, 133.7, 137.1, 145.0, 147.2, 167.1. MS (ESI) *m*/*z*: [M + H]^+^ calcd for C_27_H_32_N_5_O_5_ 506.2; found 506.2.

#### Reaction of MBHA derivative 1b with *N*-acetylhistidine

A mixture of 1b (100 mg, 0.324 mmol) in acetonitrile (25 mL) and water (5.0 mL) containing phosphate buffered saline (PBS, pH 7.4) powder (Aldrich, 50 mg) and *N*-acetylhistidine^21^ (96 mg, 0.486 mmol) was heated to reflux temperature for 4 days. The reaction mixture was concentrated under reduced pressure and extracted with dichloromethane. The organic layer was washed with brine, dried over sodium sulfate, and concentrated under reduced pressure. The residue was purified by flash chromatography with dichloromethane–methanol (9 : 1) as the eluent to afford compound 3c as an off-white solid (90 mg, yield 80%, mp 140 °C dec). ^1^H NMR (500 MHz, CDCl_3_): 1.74 (s, 3H), 3.01–3.11 (m, 2H), 3.20 (s, 1H), 3.21 (s, 1H), 3.78 (s, 6H), 4.10–4.15 (m, 1H), 5.08 (d, *J* = 14.6, 1H), 5.17 (d, *J* = 14.6, 1H), 5.28 (d, *J* = 15.0, 1H), 5.40 (d, *J* = 15.0, 1H), 7.17 (s, 2H), 7.34 (d, *J* = 8.8, 1H), 7.38 (d, *J* = 8.5, 1H), 7.53 (d, *J* = 8.2, 2H), 7.77–7.88 (m, 6H), 7.93 (s, 1H), 7.95 (s, 1H), 8.15 (s, 2H), 8.27 (s, 1H). ^13^C NMR (125 MHz, CDCl_3_): 23.2, 26.3, 44.0, 45.9, 52.9, 53.6, 79.0, 83.5, 120.4, 121.6, 123.5, 124.4, 126.0, 126.5, 128.8, 129.0, 129.1, 129.2, 129.7, 129.9, 130.0, 130.9, 131.0, 131.9, 132.5, 132.6, 133.0, 133.2, 134.1, 134.9, 147.1, 166.3, 170.3, 172.3. MS (ESI) *m*/*z*: [M + H]^+^ calcd for C_42_H_36_N_3_O_7_ 694.3; found 693.7.

Compound 2c was obtained from the above flash chromatography purification (as a more polar fraction) by using dichloromethane–methanol (8 : 2) as the eluent (8.0 mg, yield 5.5%). An analytical sample of compound 2c was prepared by recrystallization from methanol by slow evaporation to obtain rosettes of white needles (mp 180 °C dec). ^1^H NMR (500 MHz, DMSO-d_6_): 1.71 (s, 3H), 2.64–2.69 (m, 1H), 2.82–2.92 (m, 1H), 3.75 (s, 3H), 4.12–4.23 (m, 1H), 4.37 (s, 1H), 4.98 (s, 2H), 6.74 (s, 1H), 7.47 (s, 1H), 7.53–7.67 (m, 3H), 7.91–8.02 (m, 3H), 8.06 (s, 1H), 8.14 (s, 1H). ^13^C NMR (125 MHz, DMSO-d_6_): 23.2, 31.3, 43.1, 52.9, 54.0, 82.6, 84.0, 116.2, 120.9, 127.6, 127.8, 128.8, 129.5, 129.7, 131.9, 132.6, 132.7, 132.9, 137.0, 139.4, 143.7, 167.1, 169.0, 176.3. MS (ESI) *m*/*z*: [M + H]^+^ calcd for C_25_H_24_N_5_O_5_ 446.2; found 445.8.

#### 2-(Methoxycarbonyl)-1-[6-(prop-2-ynyloxy)naphthalen-2-yl]allyl 2,5,8,11,14,17,20,23,26,29,32,35-dodecaoxaoctatriacontan-38-oate (1c)

A mixture of O-(2-carboxyethyl)-O′-methyl-undecaethylene glycol (250 mg, 0.425 mmol) and SOCl_2_ (1.0 mL) in dry dichloromethane (5.0 mL) was heated under reflux for 1.5 h and then concentrated under reduced pressure. The residue was dissolved in dry dichloromethane (5.0 mL) and the resulting solution was added to a mixture of compound 6a (187 mg, 0.631 mmol) and TEA (0.12 mL, 0.84 mmol) in dry dichloromethane (5.0 mL). After stirring at room temperature for 2 h, the reaction mixture was washed with brine. The organic layer was dried over Na_2_SO_4_ and the concentrated under reduced pressure. Purification of the residue by flash chromatography with CHCl_3_–MeOH (95 : 5) as the eluent afforded compound 1c (245 mg, yield 67%) as a pale yellow oil. ^1^H NMR (400 MHz, CDCl_3_): 2.54 (t, *J* = 2.3 Hz, 1H), 2.58–2.70 (m, 2H), 3.37 (s, 3H), 3.52–3.78 (m, 49H), 4.79 (d, *J* = 2.3 Hz, 2H), 5.96 (s, 1H), 6.41 (s, 1H), 6.82 (s, 1H), 7.17 (dd, *J* = 8.8, 2.4, 1H), 7.20 (d, *J* = 2.0, 1H), 7.44 (d, *J* = 8.4, 1H), 7.69–7.76 (m, 3H). ^13^C NMR (125 MHz, CDCl_3_): 35.3, 52.0, 55.8, 59.1, 66.6, 70.4, 70.6, 71.9, 73.4, 75.8, 78.3, 107.3, 119.1, 125.8, 126.0, 127.1, 127.3, 128.9, 129.9, 133.2, 134.2, 139.4, 155.9, 165.4, 170.1. MS (ESI) *m*/*z*: [M + Na]^+^ calcd for C_44_H_66_NaO_17_ 889.4; found 889.6.

#### Reaction of MBHA derivative 1c with imidazole

A mixture of 1c (10 mg, 0.0115 mmol) in water (2.0 mL) containing phosphate buffered saline (PBS, pH 7.4) powder (Aldrich, 20 mg) and imidazole (1.2 mg, 0.0176 mmol) was stirred at room temperature for 36 h, then diluted with brine, and extracted with chloroform. The organic layer was dried over sodium sulfate and concentrated under reduced pressure. The residue was purified by flash chromatography with chloroform–methanol (9 : 1) to obtain compound 3a as a white solid (3.5 mg, yield 92%).

#### 1-(6-Ethynylnaphthalen-2-yl)-2-(methoxycarbonyl)allyl 2,5,8,11,14,17,20,23,26,29,32,35-dodecaoxaoctatriacontan-38-oate (1d)

A mixture of O-(2-carboxyethyl)-O′-methyl-undecaethylene glycol (250 mg, 0.425 mmol) and SOCl_2_ (1.0 mL) in dry dichloromethane (5.0 mL) was heated under reflux for 1.5 h and then concentrated under reduced pressure. The residue was dissolved in dry dichloromethane (5.0 mL) and the resulting solution was added to a mixture of compound 6b (168 mg, 0.631 mmol) and TEA (0.12 mL, 0.84 mmol) in dry dichloromethane (5.0 mL). After stirring at room temperature for 2 h, the reaction mixture was washed with brine and the organic layer was dried over Na_2_SO_4_ and concentrated under reduced pressure. The residue was purified by flash chromatography with CHCl_3_–MeOH (95 : 5) as the eluent to afford compound 1d (204 mg, yield 57%) as a colorless oil. ^1^H NMR (500 MHz, CDCl_3_): 2.66 (m, 2H), 3.15 (s, 1H), 3.37 (s, 3H), 3.53–3.66 (m, 44H), 3.69 (s, 3H), 3.75 (t, *J* = 6.3, 2H), 5.98 (s, 1H), 6.44 (s, 1H), 6.83 (s, 1H), 7.47–7.52 (m, 2H), 7.76 (d, *J* = 8.4, 2H), 7.82 (s, 1H), 7.98 (s, 1H). ^13^C NMR (125 MHz, CDCl_3_): 35.3, 52.1, 59.1, 66.6, 70.4, 70.6, 71.9, 73.3, 77.8, 83.8, 119.9, 126.0, 126.2, 127.0, 128.2, 128.3, 129.0, 132.0, 132.6, 132.7, 136.3, 139.2, 165.3, 170.1. MS (ESI) *m*/*z*: [M + Na]^+^ calcd for C_43_H_64_NaO_16_ 859.4; found 858.8.

#### Reaction of MBHA derivative 1d with imidazole

A mixture of 1d (50 mg, 0.0597 mmol) in water (10 mL) containing phosphate buffered saline (PBS, pH 7.4) powder (Aldrich, 100 mg) and imidazole (6.1 mg, 0.0898 mmol) was stirred at room temperature for 36 h, then diluted with brine, and extracted with chloroform. The organic layer was dried over sodium sulfate and concentrated under reduced pressure. The residue was purified by flash chromatography with chloroform–methanol (9 : 1) to obtain compound 3b as an off-white solid (14 mg, yield 78%).

#### *N*-Acetylhexahistidine (Ac-His-6)^22^

**Fig. 16:**
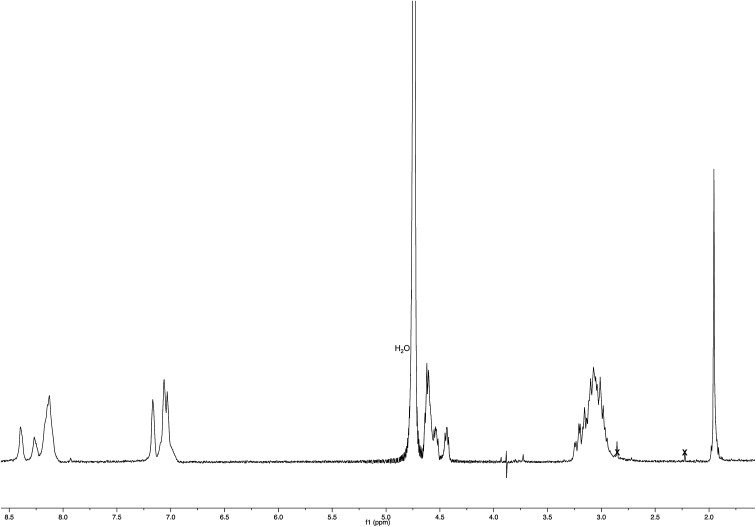
^1^H NMR spectrum (400 MHz, D_2_O – PBS) of Ac-His-6.

The synthesis of linear Ac-His-6 was performed according to the solid phase approach using standard Fmoc methodology in a CEM Liberty automated microwave peptide synthesizer. *N*_α_-Fmoc-*N*_(im)_-trityl-l-histidine, Wang-resin supported *N*_α_-Fmoc-*N*_(im)_-trityl-l-histidine and HBTU were purchased from Chem-Impex International (Illinois, USA). HOBt, DIPEA, piperidine, trifluoroacetic acid, acetic anhydride and solvents (DMF and dichloromethane) for peptide synthesis were obtained from Sigma Aldrich. Activation of the resin: 167 mg of Wang-resin supported amino acid were swollen in 20 mL of DMF for 3 h, isolated by filtration, and charged in the instrument. The synthesizer was then charged with DMF (886 mL), dichloromethane (83 mL) and the solutions of *N*_α_-Fmoc-*N*_(im)_-trityl-l-histidine (1.86 g in 15 mL of DMF), activator (490 mg of HOBt and 1.33 g of HBTU in 7 mL of DMF), activator base (1.74 mL of DIPEA in 3.26 mL of DMF), deprotector (20 mL of piperidine in 80 mL of DMF), and of acylating agent (2.0 mL of acetic anhydride in 8.0 mL of DMF). The *N*_α_-Fmoc-*N*_(im)_-trityl-l-histidine was added stepwise, according to the desired sequence. Each coupling reaction was accomplished with HBTU and HOBt in the presence of DIPEA. The *N*_α_-Fmoc protecting groups were removed by treating the protected peptide resin with a 20% solution of piperidine in DMF (v/v). The *N*-terminal Fmoc group was removed as described above, and the acetylation of the free amine functionality was obtained by treating with a 20% solution of acetic anhydride in DMF (v/v). The peptide was released from the resin with TFA/i-Pr_3_SiH/H_2_O (95 : 2.5 : 2.5) for 3 h. Also the N_im_-trityl group was removed during the release from the resins. The resin was removed by filtration, and the crude peptide was purified by precipitation in cold anhydrous diethyl ether from a solution containing the minimum amount of methanol to give Ac-His-6 as a white powder. ^1^H NMR (400 MHz, D_2_O – PBS): Fig. 16. MS: Fig. 3.

#### Reaction of MBHA derivative 1d with Ac-His-6

A mixture of 1d (40 mg, 0.0478 mmol) in deuterium oxide (8.0 mL) containing phosphate buffered saline (PBS, pH 7.4) powder (Aldrich, 80 mg) and Ac-His-6 (4.2 mg, 0.00476 mmol) was stirred at room temperature and the reaction progress was monitored by recording ^1^H NMR experiments at regular time intervals. After stirring the reaction mixture at room temperature for three weeks, it was extracted with chloroform. The organic layer was treated with brine, dried over sodium sulfate, and concentrated under reduced pressure. The residue was purified by flash chromatography with chloroform–methanol (8 : 2) to obtain polymeric material Ac-His-6-MBHA-1d as a brown glassy solid (10.5 mg). ^1^H NMR (500 MHz, CDCl_3_): Fig. 5. ^13^C NMR (125 MHz, CDCl_3_): Fig. 6. MS: Fig. 4 and ESI-1.†

#### Methyl 2-[[6-[1-(2,5,8,11,14,17,20,23,26-nonaoxaoctacosan-28-yl)-1*H*-1,2,3-triazol-4-yl]naphthalen-2-yl](acetoxy)methyl]acrylate (1e)

To a mixture of compound 1b (136 mg, 0.44 mmol) and 28-azido-2,5,8,11,14,17,20,23,26-nonaoxaoctacosane (100 mg, 0.22 mmol) in dry THF (10 mL), DIPEA (15 μL, 0.088 mmol) and CuBr (12 mg, 0.088 mmol) were added. The reaction was stirred at room temperature for 6 h and then concentrated under reduced pressure. The residue was dissolved in dichloromethane and washed with brine. The organic layer was dried over sodium sulfate and concentrated under reduced pressure and the resulting residue was purified by flash chromatography with ethyl acetate–methanol (9 : 1) as the eluent to afford compound 1e (120 mg, yield 72%) as a pale yellow oil.

^1^H NMR (500 MHz, CDCl_3_): 2.13 (s, 3H), 3.36 (s, 3H), 3.50–3.54 (m, 2H), 3.58–3.64 (m, 30H), 3.70 (s, 3H), 3.92–3.94 (m, 2H), 4.60–4.63 (m, 2H), 5.94 (s, 1H), 6.45 (s, 1H), 6.84 (s, 1H), 7.47 (dd, *J* = 8.5, 1.6, 1H), 7.83–7.88 (m, 3H), 7.94 (dd, *J* = 8.5, 1.4, 1H), 8.12 (s, 1H), 8.34 (s, 1H). ^13^C NMR (125 MHz, CDCl_3_): 21.2, 50.5, 52.1, 59.1, 69.5, 70.5, 71.9, 73.2, 121.5, 124.0, 124.4, 125.8, 126.0, 127.0, 128.6, 128.7, 128.8, 132.7, 133.4, 135.3, 139.5, 147.5, 165.4, 169.5. MS (ESI) *m*/*z*: [M + Na]^+^ calcd for C_38_H_55_N_3_NaO_13_ 784.4; found 784.3.

#### Reaction of MBHA derivative 1e with Ac-His-6

**Fig. 17:**
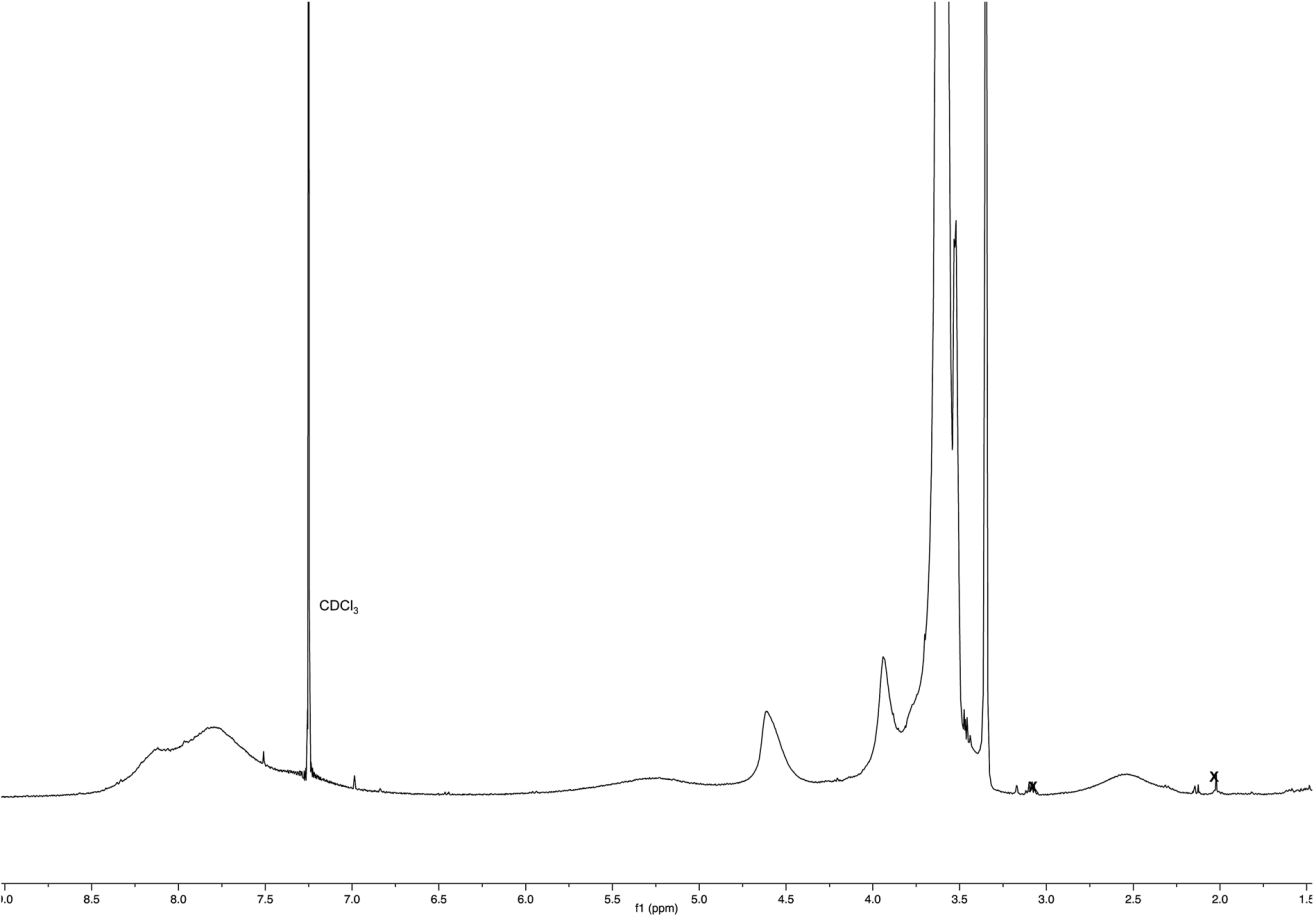
^1^H NMR spectrum (CDCl_3_) of Ac-His-6-MBHA-1e obtained by reaction of Ac-His-6 with a ten-fold excess of 1e.

A mixture of 1e (36 mg, 0.0473 mmol) in deuterium oxide (8.0 mL) containing phosphate buffered saline (PBS, pH 7.4) powder (Aldrich, 80 mg) and Ac-His-6 (4.2 mg, 0.00476 mmol) was stirred at room temperature and the reaction progress was monitored by performing ^1^H NMR experiments at regular time intervals. After stirring at room temperature for two weeks, the reaction mixture was extracted with chloroform. The organic layer was treated with brine, dried over sodium sulfate, and concentrated under reduced pressure. The residue was purified by flash chromatography with chloroform–methanol (8 : 2) to obtain polymeric material Ac-His-6-MBHA-1e as a brown gummy solid (25 mg). ^1^H NMR (400 MHz, CDCl_3_): Fig. 17. ^13^C NMR (125 MHz, CDCl_3_): Fig. 9. MS: Fig. 8.

### X-ray crystallography

A single crystal of compound 3a was submitted to X-ray data collection on an Oxford-Diffraction Xcalibur Sapphire 3 diffractometer with a graphite monochromated Mo-Kα radiation (*λ* = 0.71073 Å) at 293 K. The structure was solved by direct methods implemented in SHELXS-97 program.^24^ The refinement was carried out by full-matrix anisotropic least-squares on *F*^2^ for all reflections for non-H atoms by means of the SHELXL-97 program.^25^ Crystallographic data (excluding structure factors) have been deposited with the Cambridge Crystallographic Data Centre as supplementary publication no. CCDC 1504772.†

### Mass spectrometry measurements

MALDI-TOF mass spectra were recorded both in reflectron and linear mode by means of a 4800 Proteomic Analyzer (Applied Biosystems) MALDI-TOF/TOF instrument equipped with a Nd:YAG laser at a wavelength of 355 nm with <500 ps pulse and 200 Hz firing rate. The accelerating voltage was 20 kV. External calibration was performed using an Applied Biosystems calibration mixture consisting of polypeptides with different molar mass values. The irradiance was maintained slightly above the threshold, to obtain in reflectron mode a mass resolution of about 4000–6000 FWHM (isotopic resolution was observed in the mass range from *m*/*z* 300 up to *m*/*z* 5000). Mass accuracy was about 50–100 ppm. All measurements were performed in positive ion mode. Approximately 1500 laser shots were accumulated for each mass spectrum in reflectron mode, and 250 laser shots in linear one too. For the analysis of all the samples, several matrices such as 2-(4-hydroxyphenylazo)benzoic acid, 2,5-dihydroxybenzoic acid, and α-cyano-4-hydroxycinnamic acid were used. The best spectra were obtained using α-cyano-4-hydroxycinnamic and 2,5-dihydroxybenzoic acid, matrices, and operating in reflectron mode.

ESI mass spectra were acquired by a Thermo Scientific Exactive Plus Orbitrap MS (Thermo Fisher Scientific, San Jose, CA), using a heated electrospray ionization (HESI II) interface. Mass spectra were recorded operating both in positive and negative ion mode in the *m*/*z* range 200–4000 at a resolving power of 25 000 (full-width-at-half-maximum, at *m*/*z* 300, RFWHM), resulting in a scan rate of >1.5 scans per sec when using automatic gain control target of 1.0 × 10^6^ and a C-trap injection time of 100 ms. under the following conditions in positive mode: capillary temperature 300 °C, heater temperature 300 °C, nebulizer gas (nitrogen) with a flow rate of 20 arbitrary units; auxiliary gas flow rate of 3 arbitrary units; source voltage 3.5 kV; capillary voltage 50 V; tube lens voltage 150 V, skimmer voltage 23 V. The same negative values of source voltage, capillary voltage, tube lens voltage and skimmer voltage, were used in negative ion mode. 1 μL of each sample was injected with autosampler into the mass spectrometer, using methanol/acetonitrile (50 : 50, v/v) as solvent at a flow rate of 100 μL min^−1^. The Orbitrap MS system was tuned and calibrated in: (a) positive modes, by infusion of solutions of a standard mixture of caffeine (MM 194.1 Da), MRFA peptide (MM 423.6 Da) and ultramark-mixture 1621; (b) negative ion mode by infusion of a solution of a standard mixture of sodium dodecyl sulfate (MM 265.3), sodium taurocholate (MM 514.5) and ultramark-mixture 1621. Data acquisition and analysis were performed using of the Xcalibur software.

### Optical properties and photoluminescence

UV-vis absorption spectra are obtained with a Perkin Elmer Lambda 900 spectrometer. PL spectra are obtained with a SPEX 270 M monochromator equipped with a N_2_ cooled charge-coupled device exciting with a monochromated 450 W Xe lamp. The spectra are corrected for the instrument response. PL QY values of solutions were obtained by using quinine sulfate as the reference, with an experimental error of about 5% for values below 0.1%. PL QY of solid powders were measured with a home-made integrating sphere, with an experimental error of 10% and a sensitivity of about 0.1%, according to the procedure reported elsewhere.^26^

## Conflicts of interest

The authors declare no competing financial interest.

## Supplementary Material

RA-008-C8RA00315G-s001

RA-008-C8RA00315G-s002
