# Supplementary material for: Poly-histidine grafting leading to fishbone-like architectures
Source: RSC Adv. 2018 Feb 26;8(16):8638–56. doi: 10.1039/c8ra00315g (PMC9078612; doi:10.1039/c8ra00315g)
Supplement: RA-008-C8RA00315G-s001 [file RA-008-C8RA00315G-s001.pdf]

Electronic Supplementary Material (ESI) for RSC Advances.
This journal is © The Royal Society of Chemistry 2018

Electronic supplementary information

Poly-Histidine Grafting Leading to Fishbone-Like Architectures

Vincenzo Razzano,[a] Marco Paolino,[a] Annalisa Reale,[a] Germano Giuliani,[a] Roberto Artusi,[b] Gianfranco Caselli,[b] Michela Visintin,[b] Francesco Makovec,[b] Alessandro Donati,[a] Gianluca Giorgi,[a] Francesca Villafiorita-Monteleone,[c] Chiara Botta,[c] Andrea Cappelli.[*,a]

[a]Dipartimento di Biotecnologie, Chimica e Farmacia and European Research Centre for Drug Discovery and Development, Università di Siena, Via A. Moro 2, 53100 Siena, Italy,

[b]Rottapharm Biotech S.p.A., Via Valosa di Sopra 9, 20900 Monza, Italy,

[c]Istituto per lo Studio delle Macromolecole (CNR), Via E. Bassini 15, 20133 Milano, Italy.

E-mail: andrea.cappelli@unisi.it.

Table of contents:

ESI mass spectrum of **Ac-His-6-MBHA-1d** material page S2
NMR spectra of compounds **1a-e**, **2a-d**, **3a-c**, **5**, **6a**,**b**, **8** pages S3-S18
Photoluninescence of the polymeric materials obtained by exciting at different wavelengths page S19

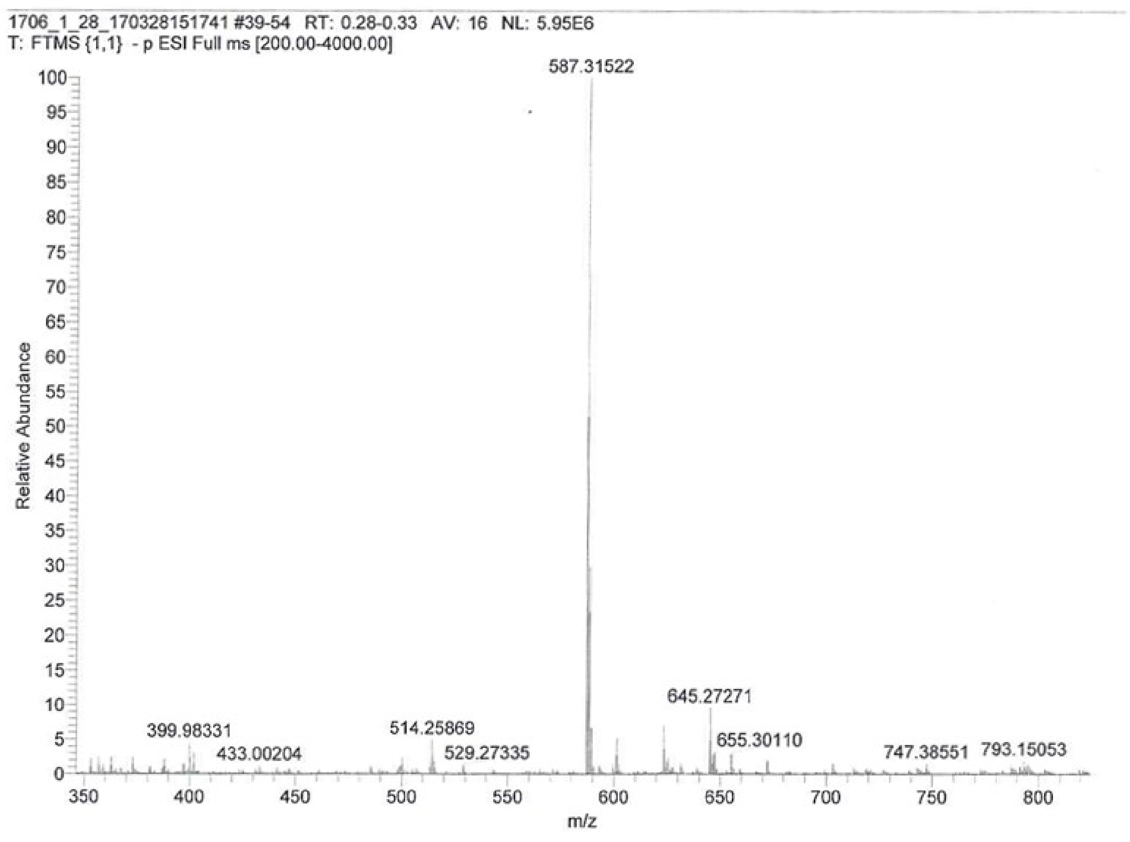

Figure ESI-1. ESI mass spectrum (negative-ion mode) of **Ac-His-6-MBHA-1d** material.

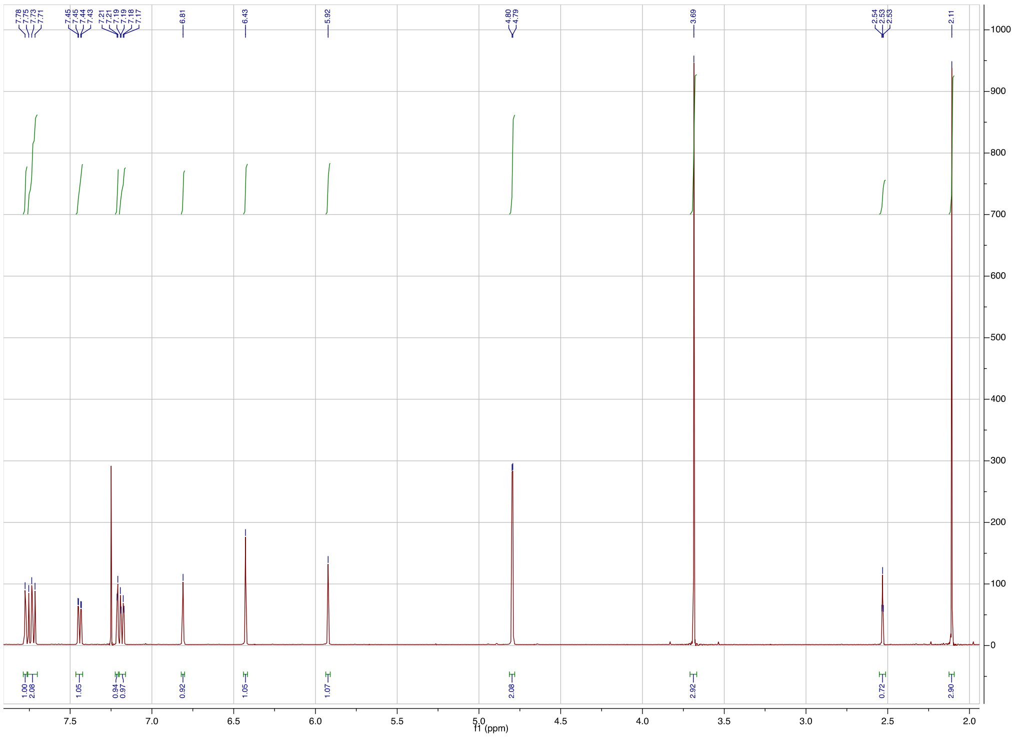

Figure ESI-2. ^{1}H NMR (500 MHz, $CDCl_3$) of methyl 2-[acetoxy[6-(prop-2-ynyloxy)naphthalen-2-yl]methyl]acrylate (**1a**).

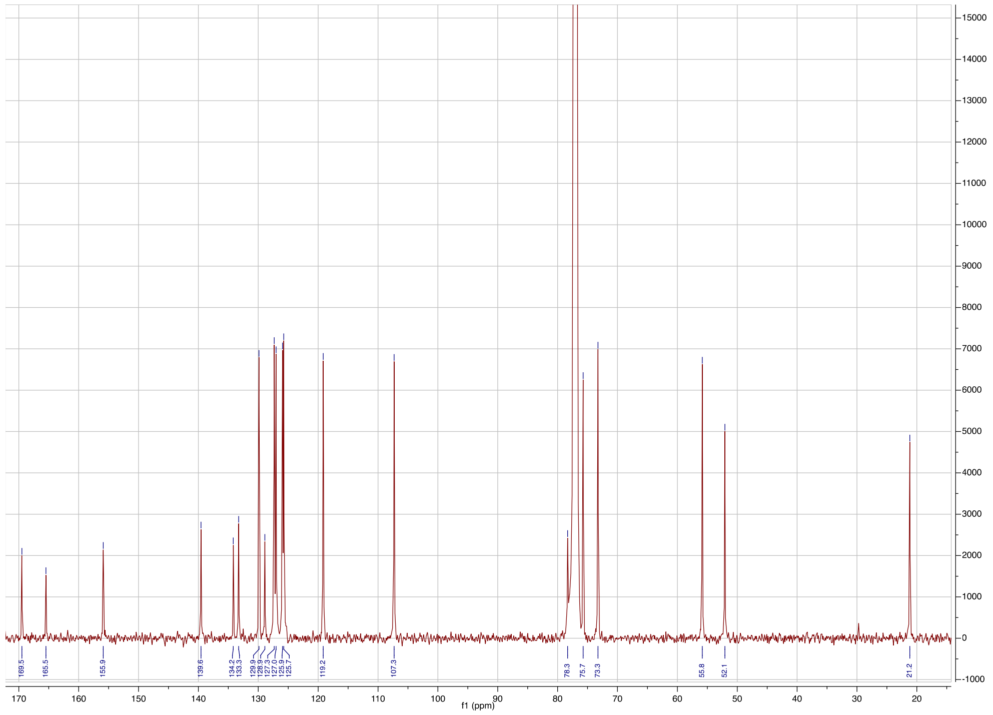

Figure ESI-3. ^{13}C NMR (125 MHz, $CDCl_3$) of methyl 2-[acetoxy[6-(prop-2-ynyloxy)naphthalen-2-yl]methyl]acrylate (**1a**).

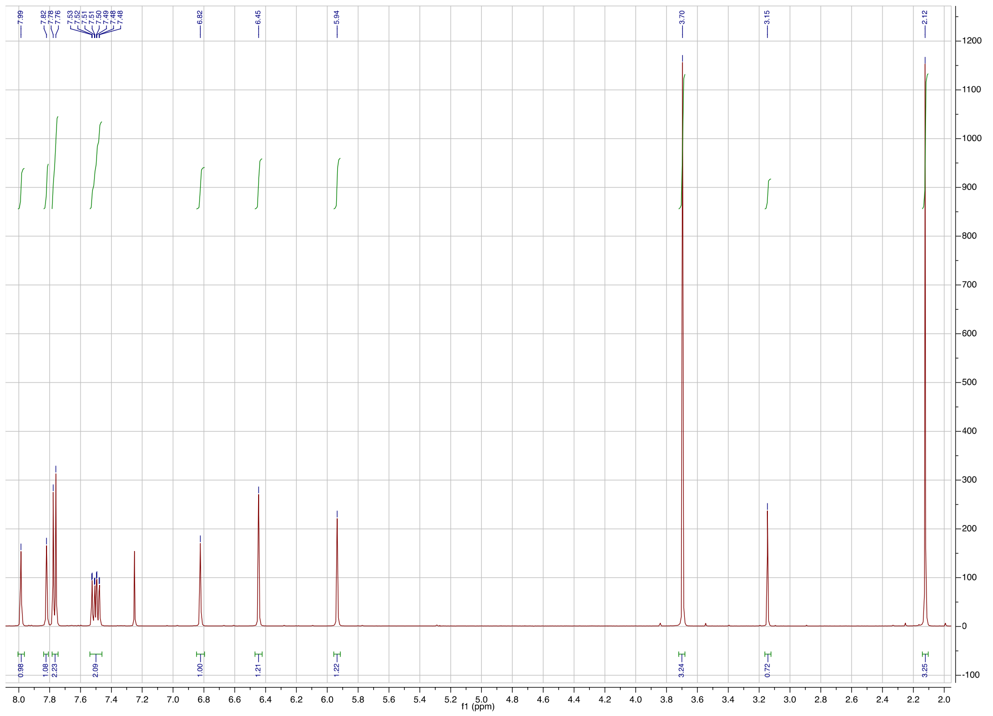

Figure ESI-4. ^{1}H NMR (500 MHz, $CDCl_3$) of methyl 2-[acetoxy(6-ethynylnaphthalen-2-yl)methyl]acrylate (**1b**).

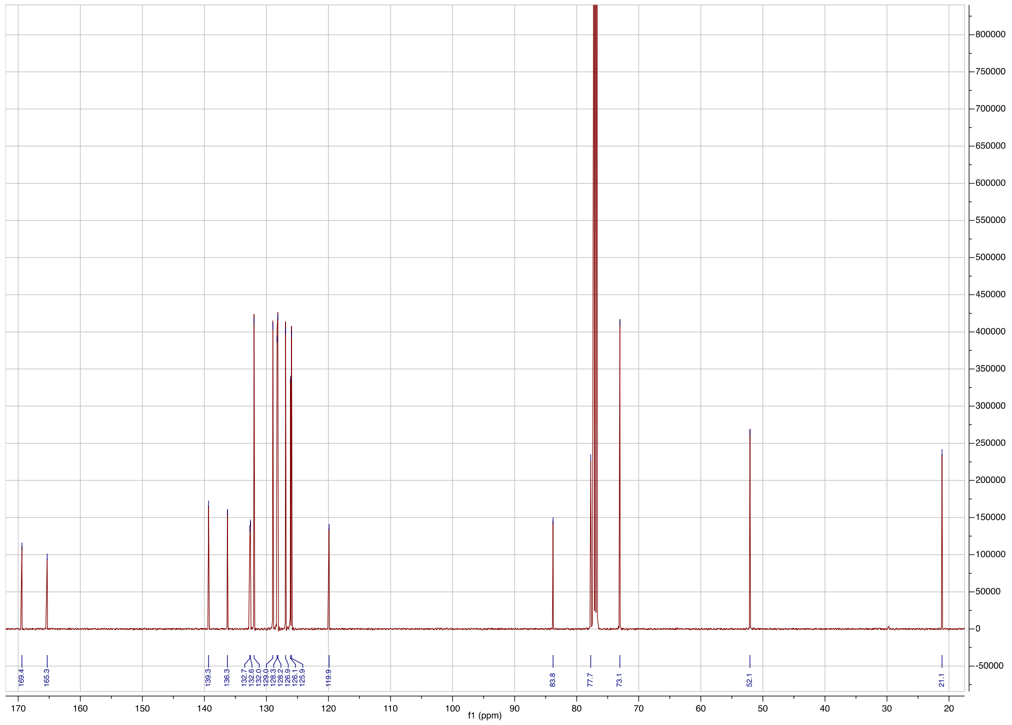

Figure ESI-5. ^{13}C NMR (125 MHz, $CDCl_3$) of methyl 2-[acetoxy(6-ethynylnaphthalen-2-yl)methyl]acrylate (**1b**).

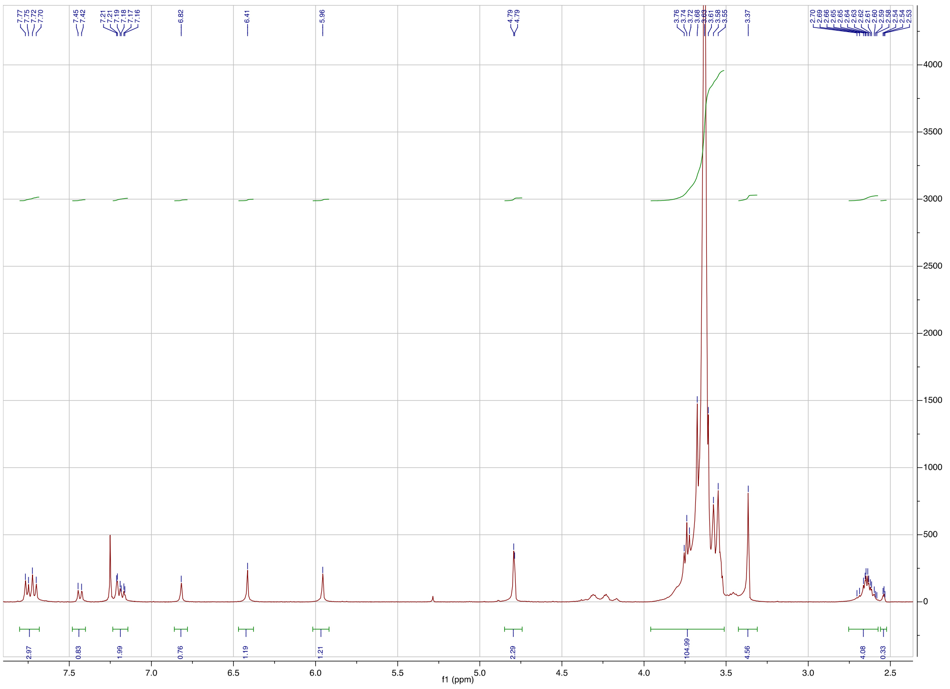

Figure ESI-6. ^{1}H NMR (400 MHz, $CDCl_3$) of 2-(methoxycarbonyl)-1-[6-(prop-2-ynyloxy)naphthalen-2-yl]allyl 2,5,8,11,14,17,20,23,26,29,32,35-dodecaoxaoctatriacontan-38-oate (**1c**).

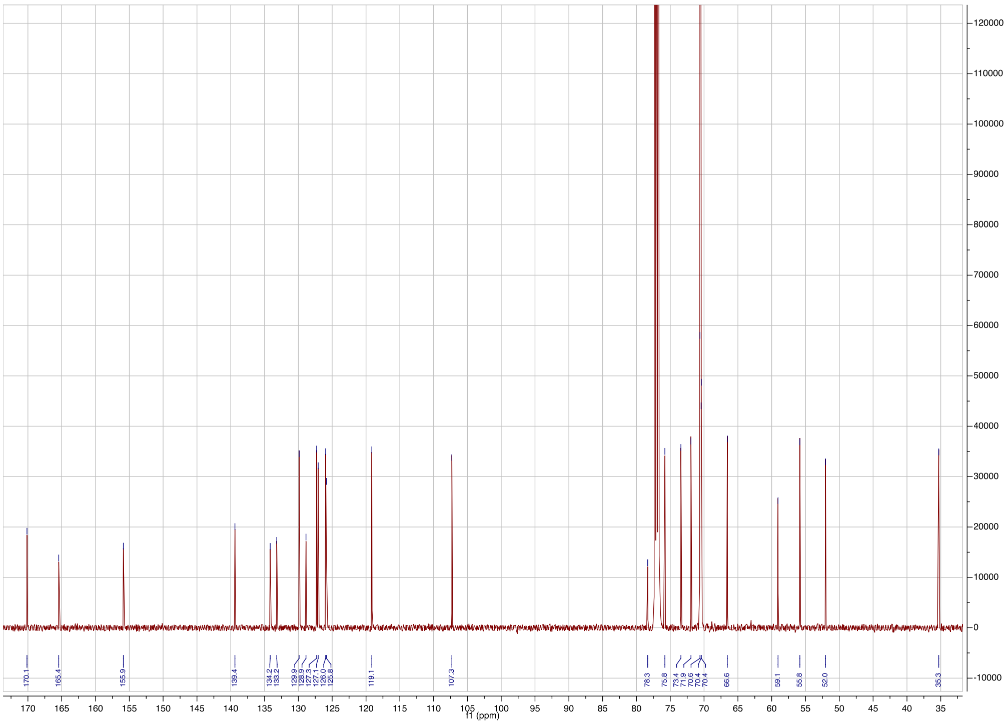

Figure ESI-7. ^{13}C NMR (125 MHz, $CDCl_3$) of 2-(methoxycarbonyl)-1-[6-(prop-2-ynyloxy)naphthalen-2-yl]allyl 2,5,8,11,14,17,20,23,26,29,32,35-dodecaoxaoctatriacontan-38-oate (**1c**).

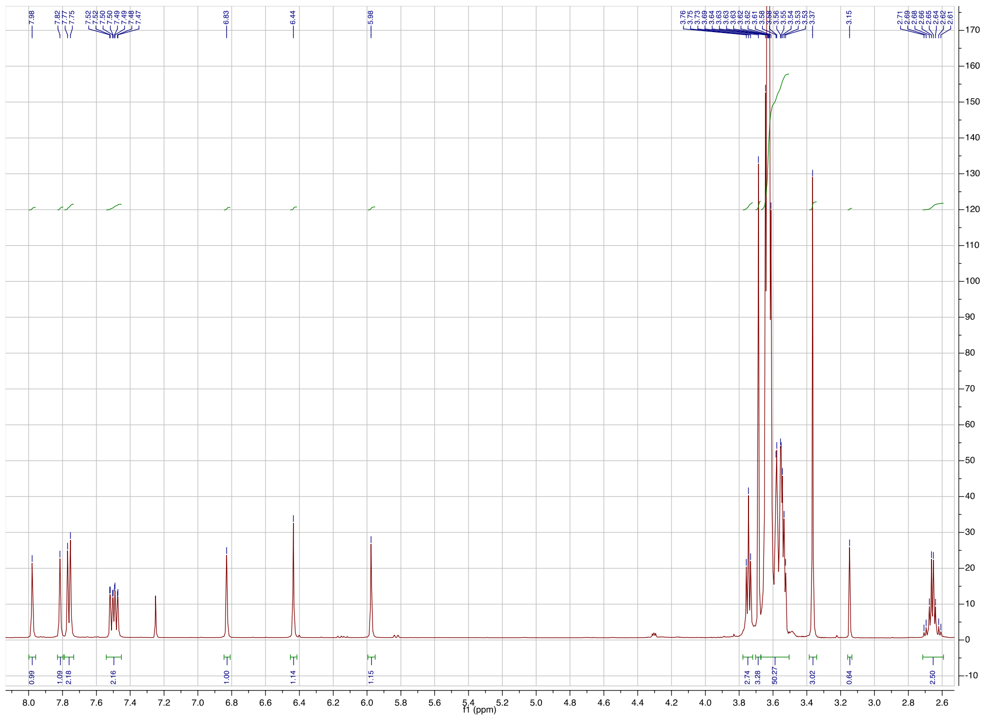

Figure ESI-8. ^{1}H NMR (500 MHz, $CDCl_3$) of 1-(6-ethynylnaphthalen-2-yl)-2-(methoxycarbonyl)allyl 2,5,8,11,14,17,20,23,26,29,32,35-dodecaoxaoctatriacontan-38-oate (**1d**).

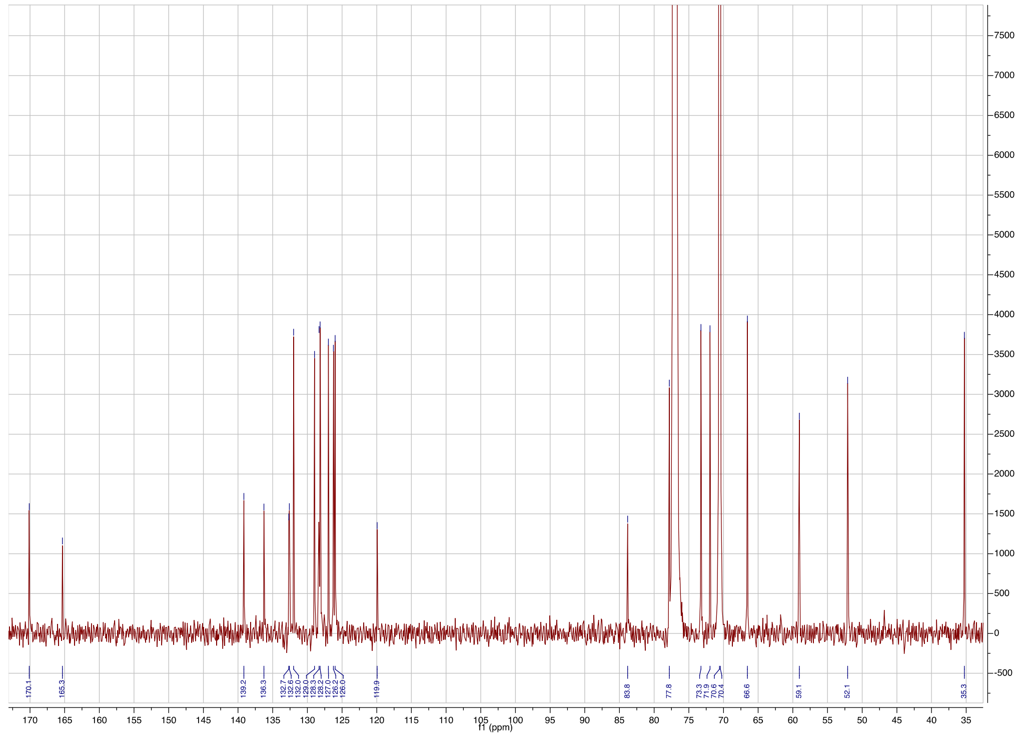

Figure ESI-9. ^{13}C NMR (125 MHz, $CDCl_3$) of 1-(6-ethynylnaphthalen-2-yl)-2-(methoxycarbonyl)allyl 2,5,8,11,14,17,20,23,26,29,32,35-dodecaoxaoctatriacontan-38-oate (**1d**).

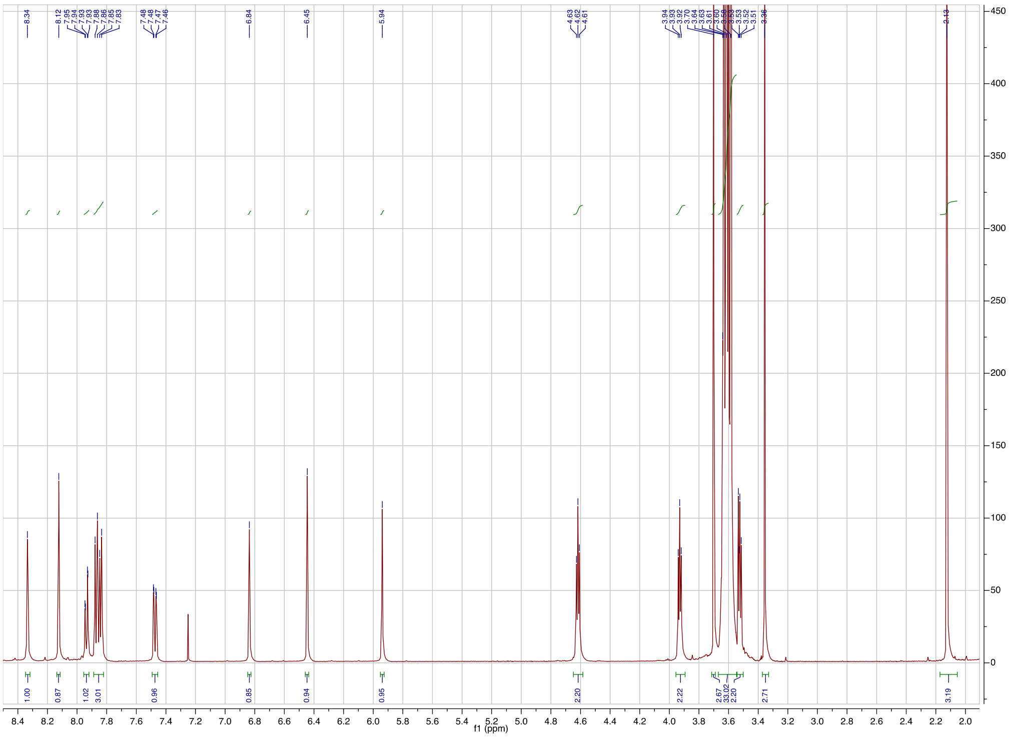

Figure ESI-10. ^{1}H NMR (500 MHz, $CDCl_3$) of methyl 2-[[6-[1-(2,5,8,11,14,17,20,23,26-nonaoxaoctacosan-28-yl)-1*H*-1,2,3-triazol-4-yl]naphthalen-2-yl](acetoxy)methyl]acrylate (**1e**).

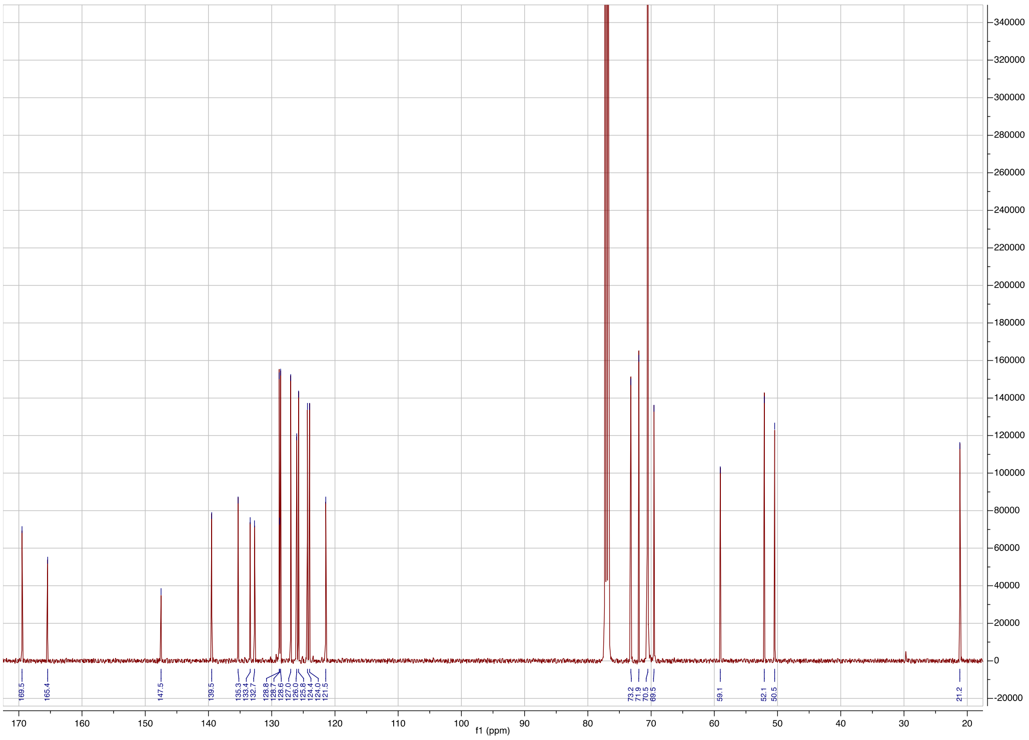

Figure ESI-11. ^{13}C NMR (125 MHz, $CDCl_3$) of methyl 2-[[6-[1-(2,5,8,11,14,17,20,23,26-nonaoxaoctacosan-28-yl)-1*H*-1,2,3-triazol-4-yl]naphthalen-2-yl](acetoxy)methyl]acrylate (**1e**).

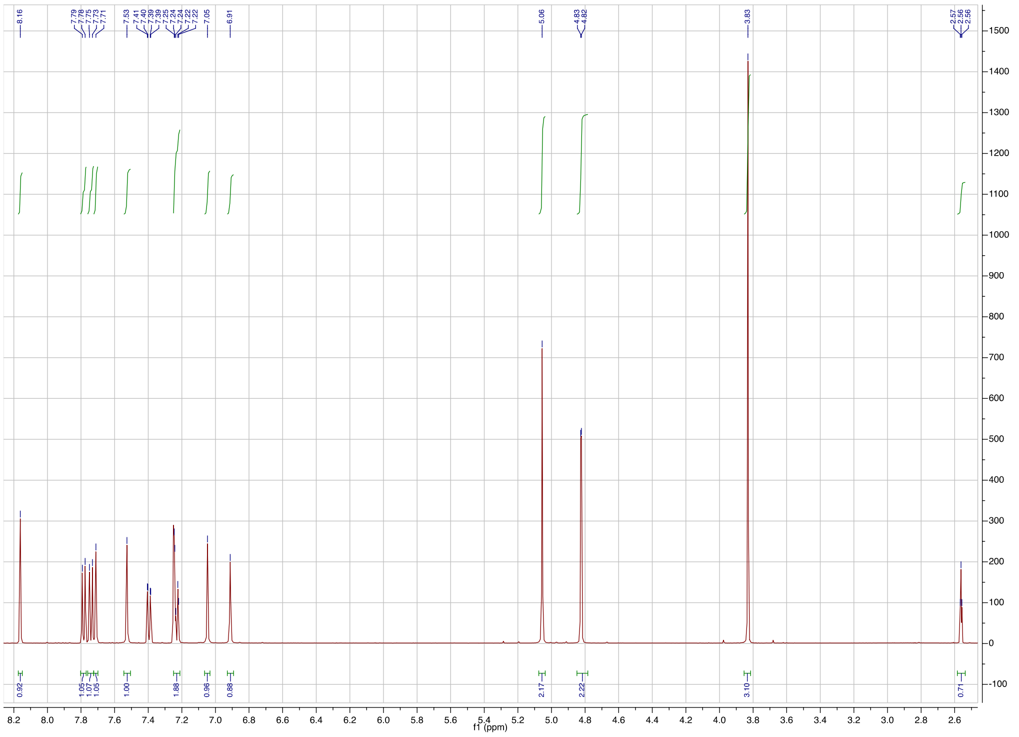

Figure ESI-12. ^{1}H NMR (500 MHz, $CDCl_3$) of (*E*)-methyl 2-[(1*H*-imidazol-1-yl)methyl]-3-[6-(prop-2-ynyloxy)naphthalen-2-yl]acrylate (**2a**).

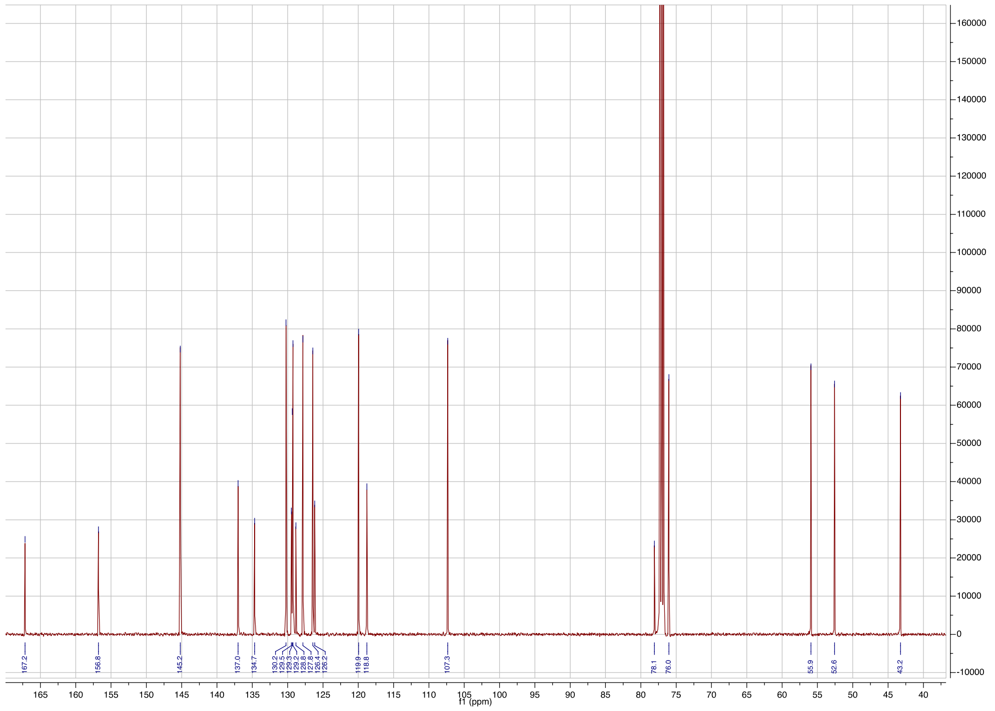

Figure ESI-13. ^{13}C NMR (125 MHz, $CDCl_3$) of (*E*)-methyl 2-[(1*H*-imidazol-1-yl)methyl]-3-[6-(prop-2-ynyloxy)naphthalen-2-yl]acrylate (**2a**).

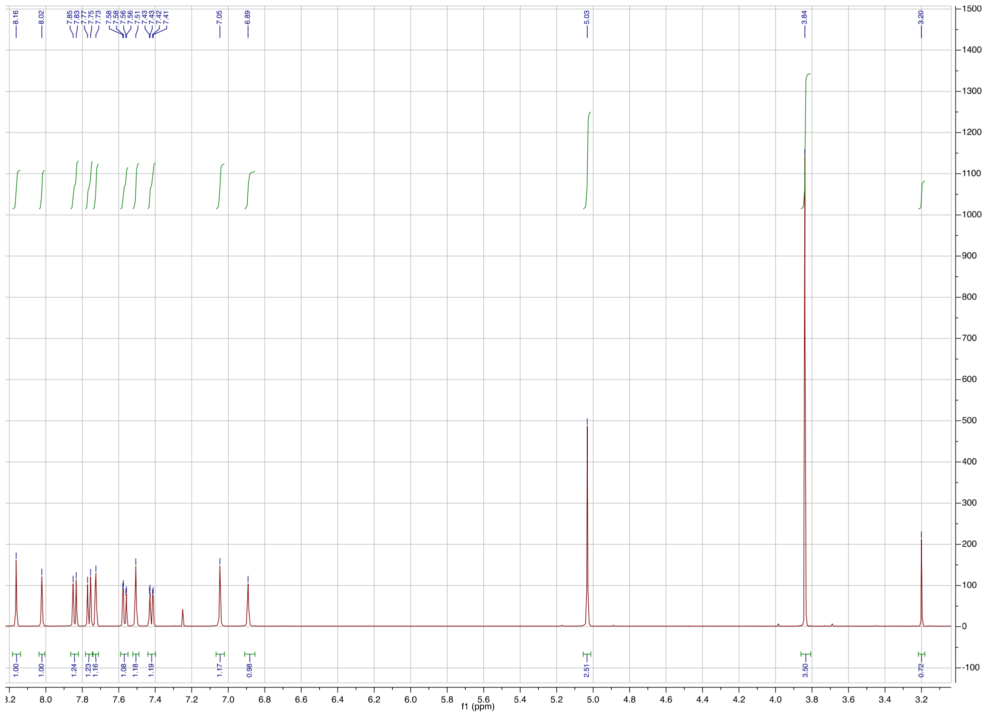

Figure ESI-14. ^{1}H NMR (500 MHz, $CDCl_3$) of (*E*)-methyl 2-[(1*H*-imidazol-1-yl)methyl]-3-(6-ethynylnaphthalen-2-yl)acrylate (**2b**).

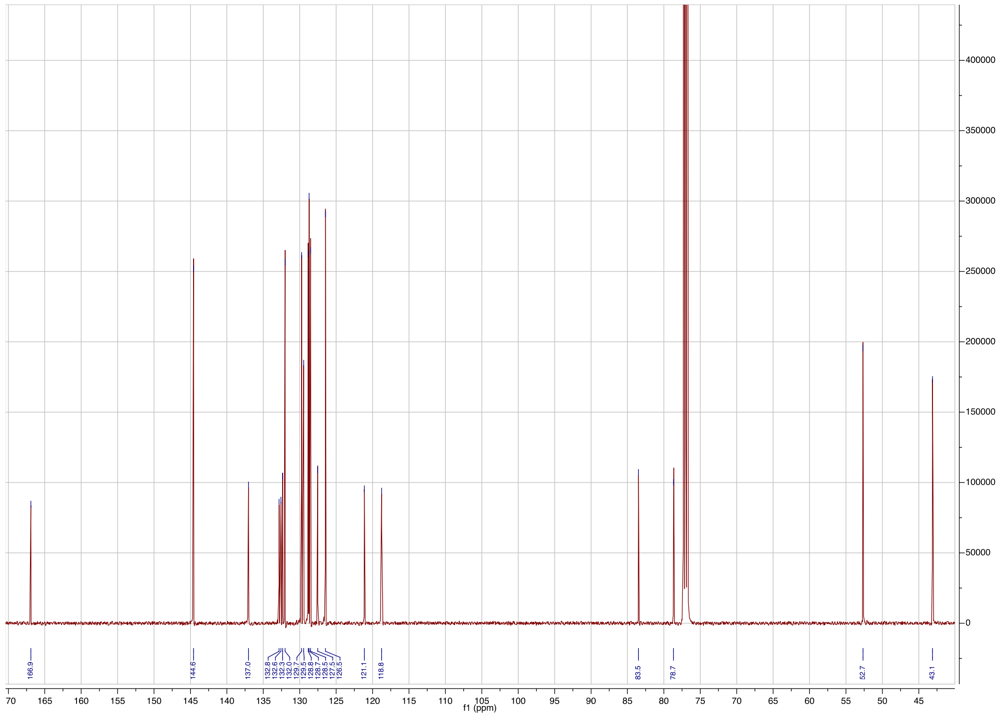

Figure ESI-15. ^{13}C NMR (125 MHz, $CDCl_3$) of (*E*)-methyl 2-[(1*H*-imidazol-1-yl)methyl]-3-(6-ethynylnaphthalen-2-yl)acrylate (**2b**).

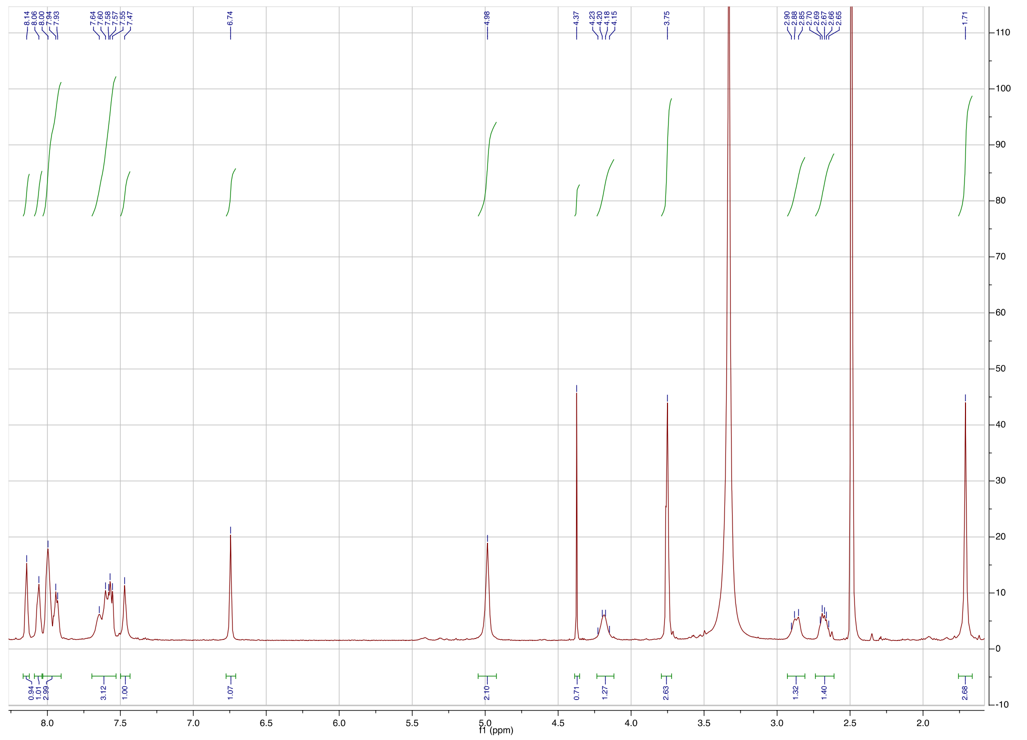

Figure ESI-16. ^{1}H NMR (500 MHz, $CDCl_3$) of (*E*)-2-acetamido-3-[1-[3-(6-ethynylnaphthalen-2-yl)-2-(methoxycarbonyl)allyl]-1*H*-imidazol-4-yl]propanoic acid (**2c**).

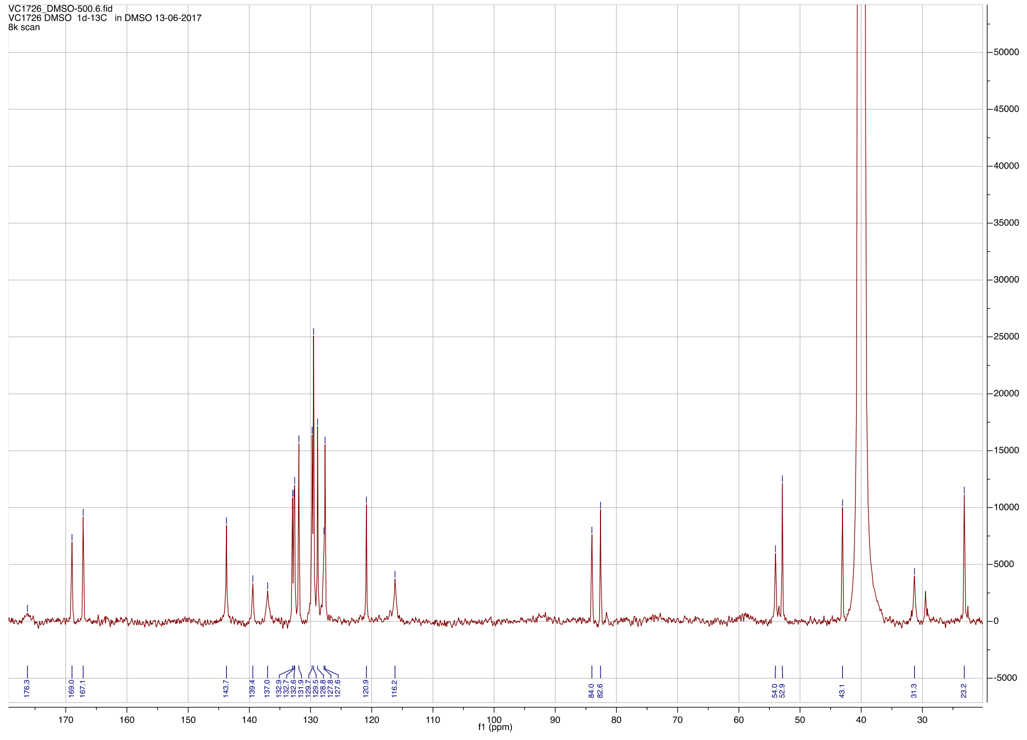

Figure ESI-17. ^{13}C NMR (125 MHz, $CDCl_3$) of (*E*)-2-acetamido-3-[1-[3-(6-ethynylnaphthalen-2-yl)-2-(methoxycarbonyl)allyl]-1*H*-imidazol-4-yl]propanoic acid (**2c**).

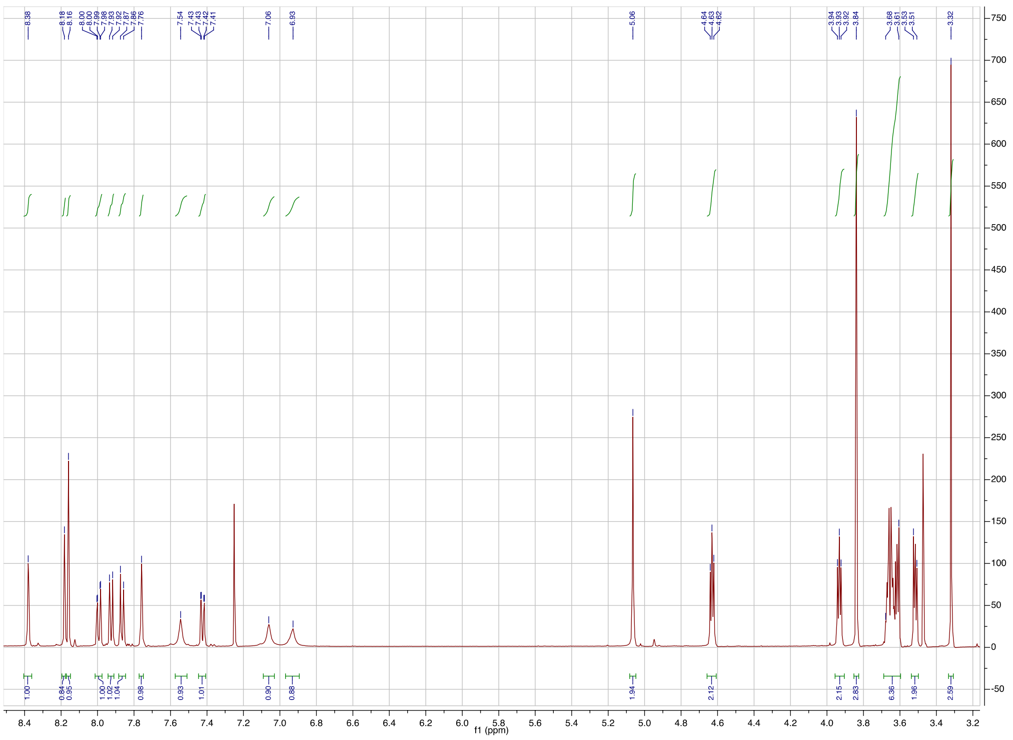

Figure ESI-18. ^{1}H NMR (500 MHz, $CDCl_3$) of (*E*)-methyl 2-[(1*H*-imidazol-1-yl)methyl]-3-[6-[1-[2-[2-(2-methoxyethoxy)ethoxy]ethyl]-1*H*-1,2,3-triazol-4-yl]naphthalen-2-yl]acrylate (**2d**).

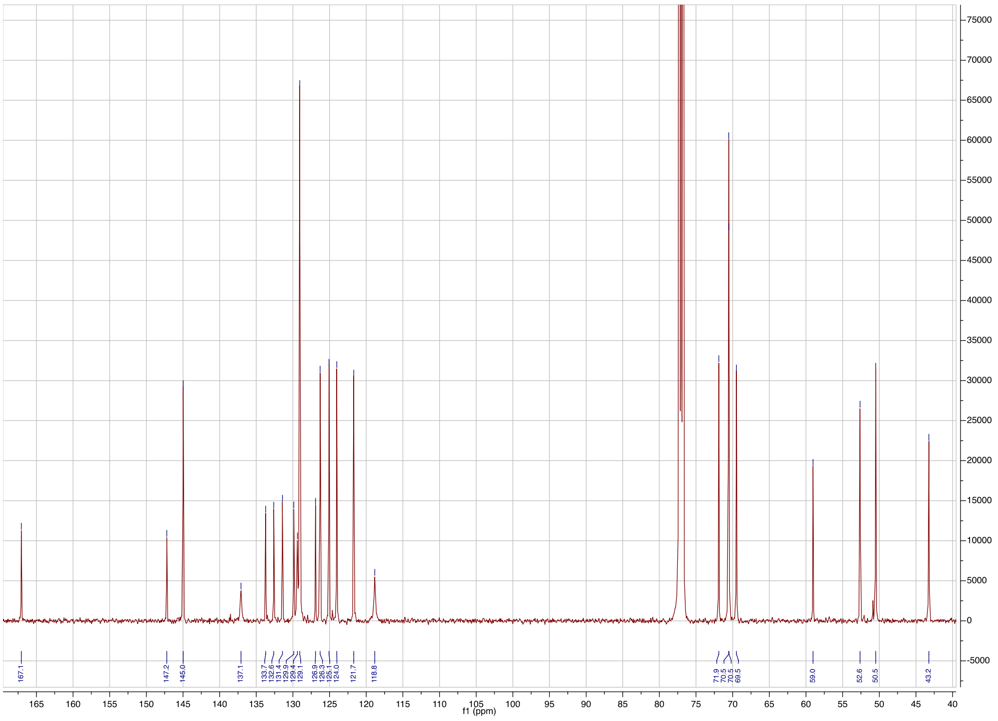

Figure ESI-19. ^{13}C NMR (125 MHz, $CDCl_3$) of (*E*)-methyl 2-[(1*H*-imidazol-1-yl)methyl]-3-[6-[1-[2-[2-(2-methoxyethoxy)ethoxy]ethyl]-1*H*-1,2,3-triazol-4-yl]naphthalen-2-yl]acrylate (**2d**).

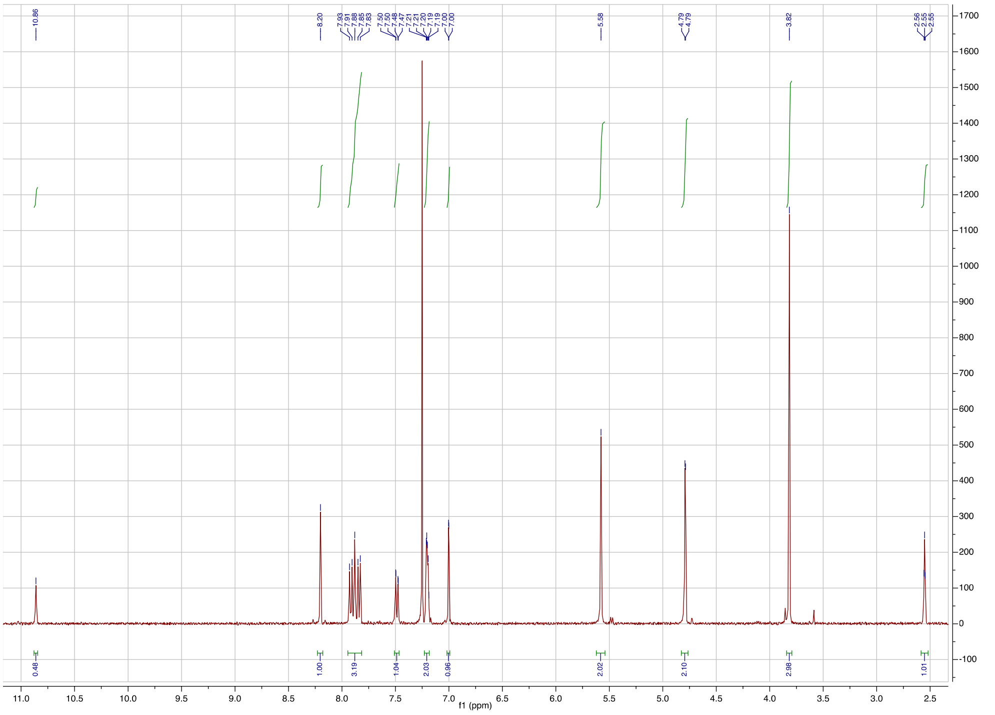

Figure ESI-20. ^{1}H NMR (400 MHz, $CDCl_3$) of 1,3-bis[[(*E*)-2-(methoxycarbonyl)-3-[6-(prop-2-ynyloxy)naphthalen-2-yl]allyl]-1*H*-imidazol-3-ium chloride (**3a**).

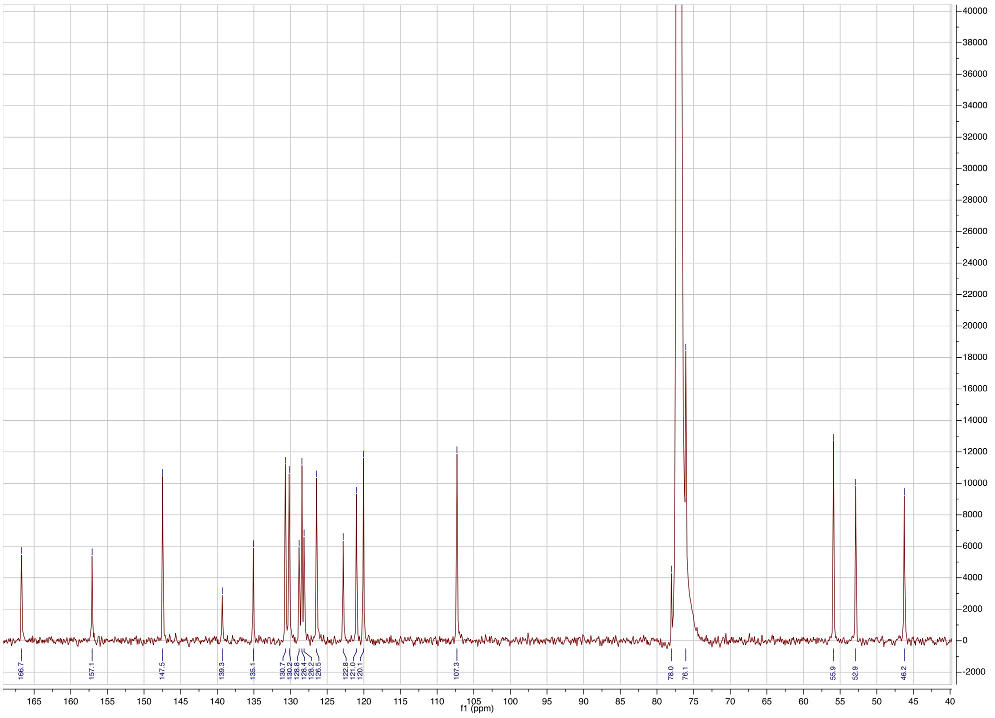

Figure ESI-21. ^{13}C NMR (125 MHz, $CDCl_3$) of 1,3-bis[[(*E*)-2-(methoxycarbonyl)-3-[6-(prop-2-ynyloxy)naphthalen-2-yl]allyl]-1*H*-imidazol-3-ium chloride (**3a**).

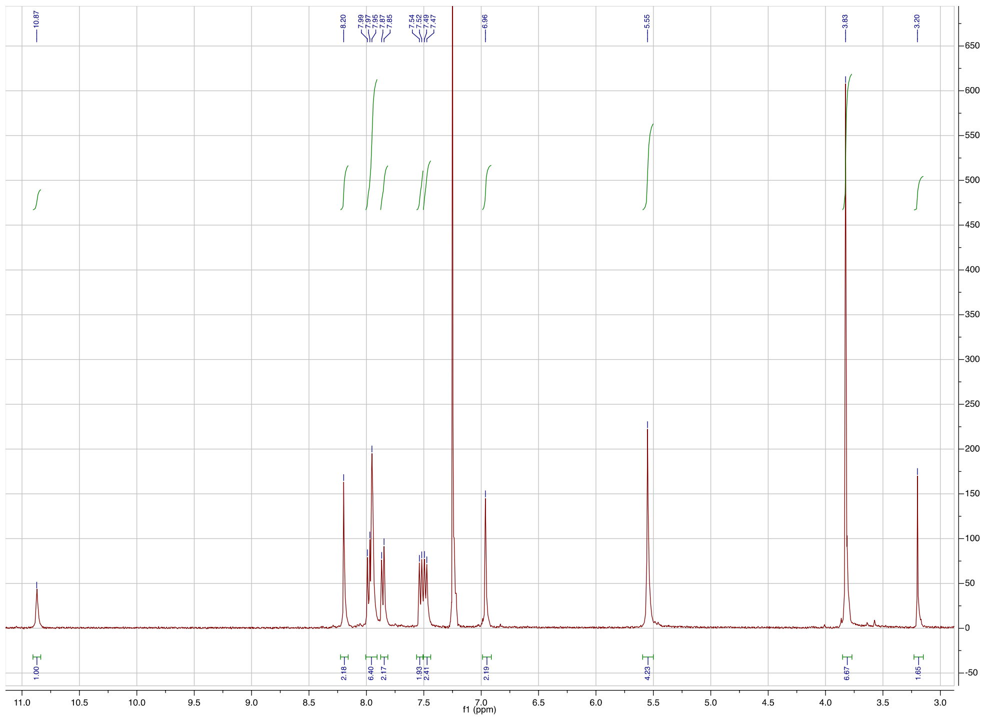

Figure ESI-22. ^{1}H NMR (400 MHz, $CDCl_3$) of 1,3-bis[[(*E*)-3-(6-ethynylnaphthalen-2-yl)-2-(methoxycarbonyl)allyl]-1*H*-imidazol-3-ium chloride (**3b**).

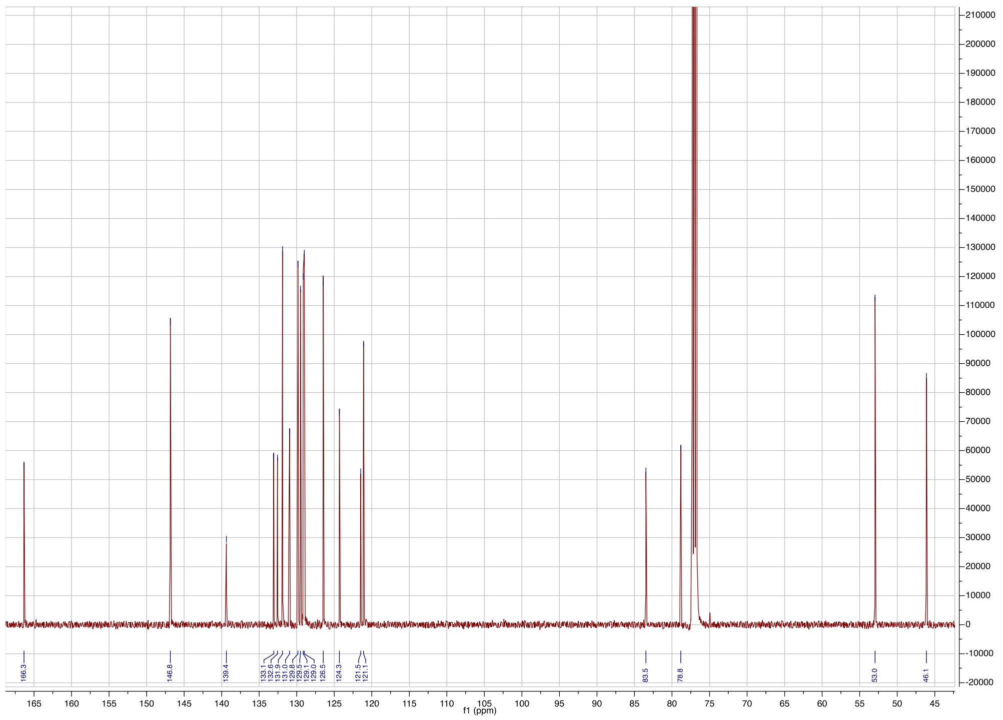

Figure ESI-23. ^{13}C NMR (125 MHz, $CDCl_3$) of 1,3-bis[[(*E*)-3-(6-ethynylnaphthalen-2-yl)-2-(methoxycarbonyl)allyl]-1*H*-imidazol-3-ium chloride (**3b**).

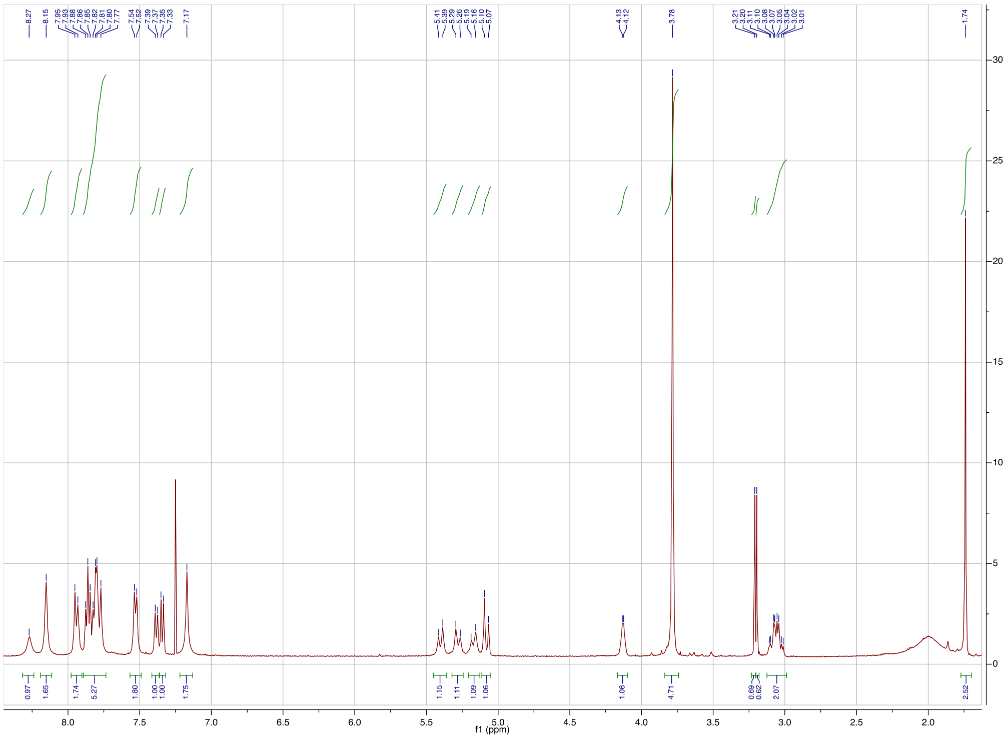

Figure ESI-24. ^{1}H NMR (500 MHz, $CDCl_3$) of 2-acetamido-3-[1,3-bis[(*E*)-3-(6-ethynylnaphthalen-2-yl)-2-(methoxycarbonyl)allyl]-1*H*-imidazol-3-ium-4-yl]propanoate (**3c**).

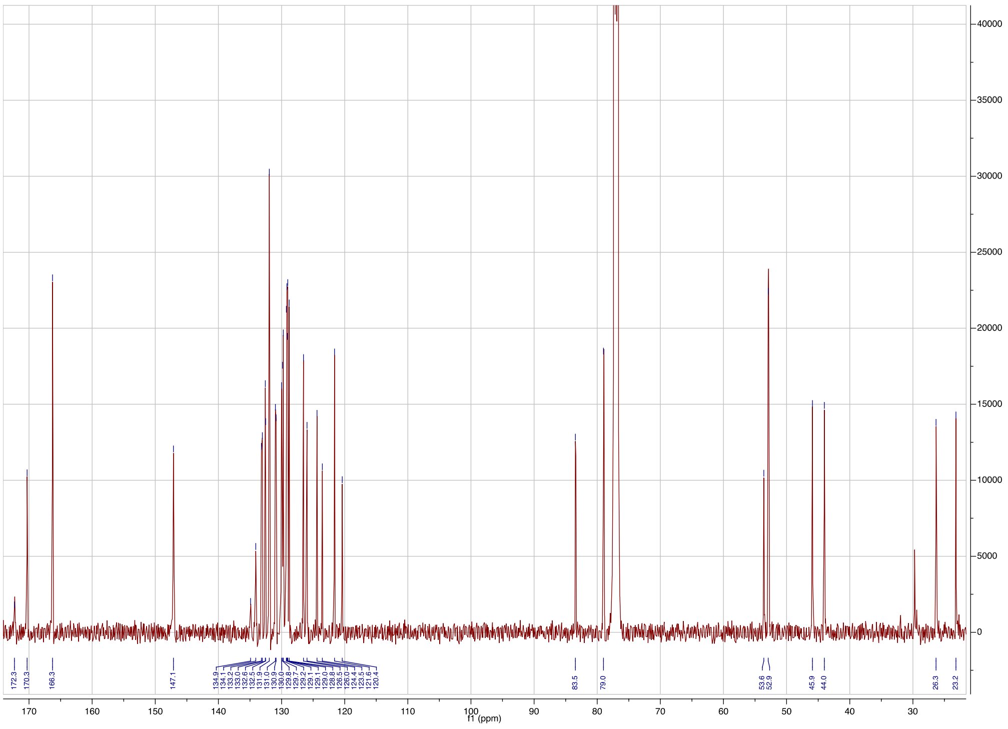

Figure ESI-25. ^{13}C NMR (125 MHz, $CDCl_3$) of 2-acetamido-3-[1,3-bis[(*E*)-3-(6-ethynylnaphthalen-2-yl)-2-(methoxycarbonyl)allyl]-1*H*-imidazol-3-ium-4-yl]propanoate (**3c**).

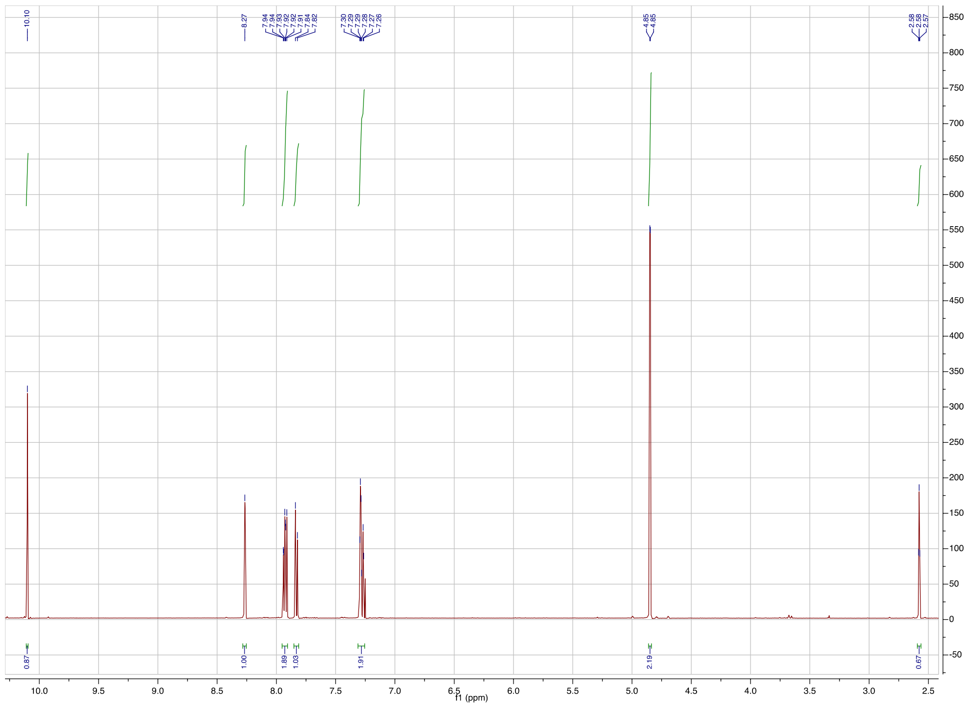

Figure ESI-26. ^{1}H NMR (500 MHz, $CDCl_3$) of 6-(prop-2-ynyloxy)-2-naphthaldehyde (**5**).

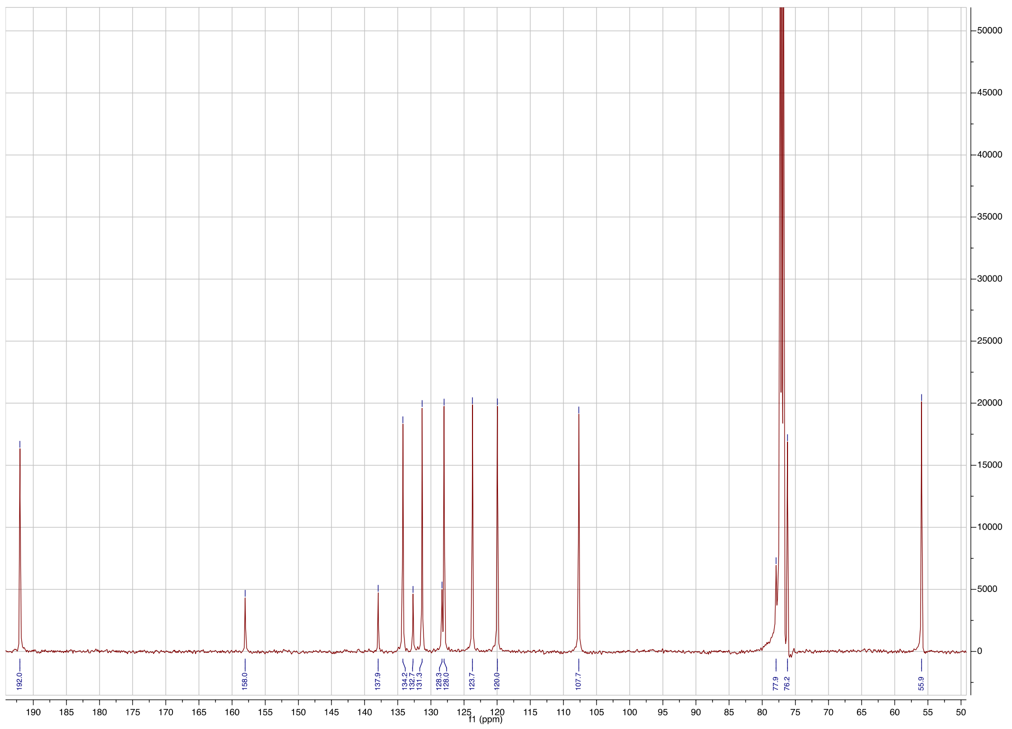

Figure ESI-27. ^{13}C NMR (125 MHz, $CDCl_3$) of 6-(prop-2-ynyloxy)-2-naphthaldehyde (**5**).

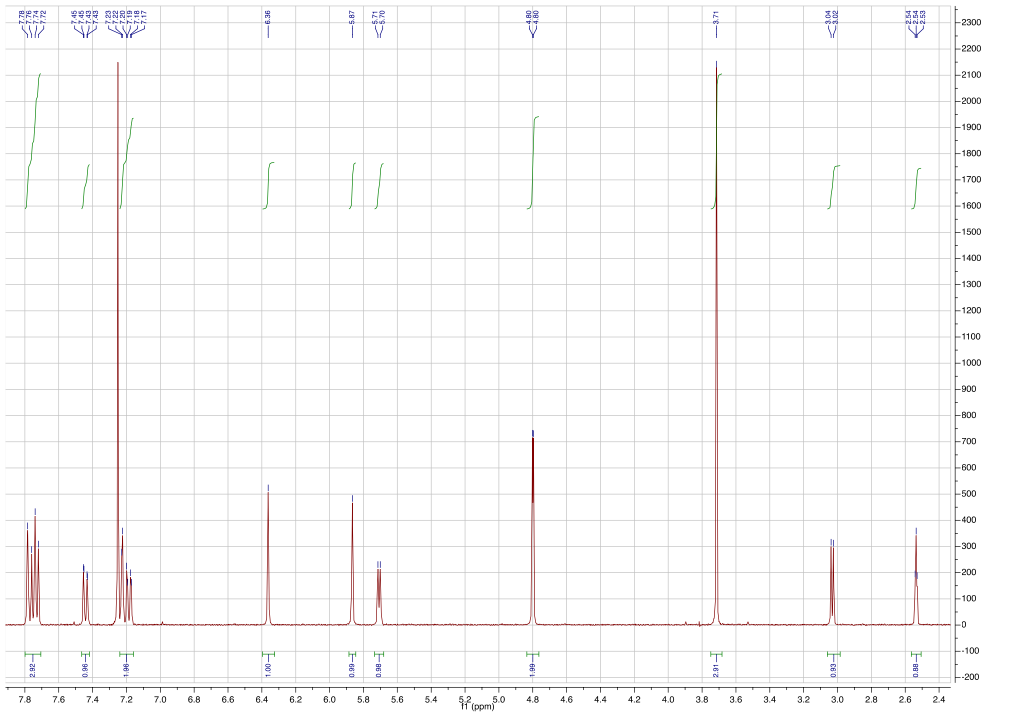

Figure ESI-28. ^{1}H NMR (400 MHz, $CDCl_3$) of methyl 2-[hydroxyl[6-(prop-2-ynyloxy)naphthalen-2-yl]methyl]acrylate (**6a**).

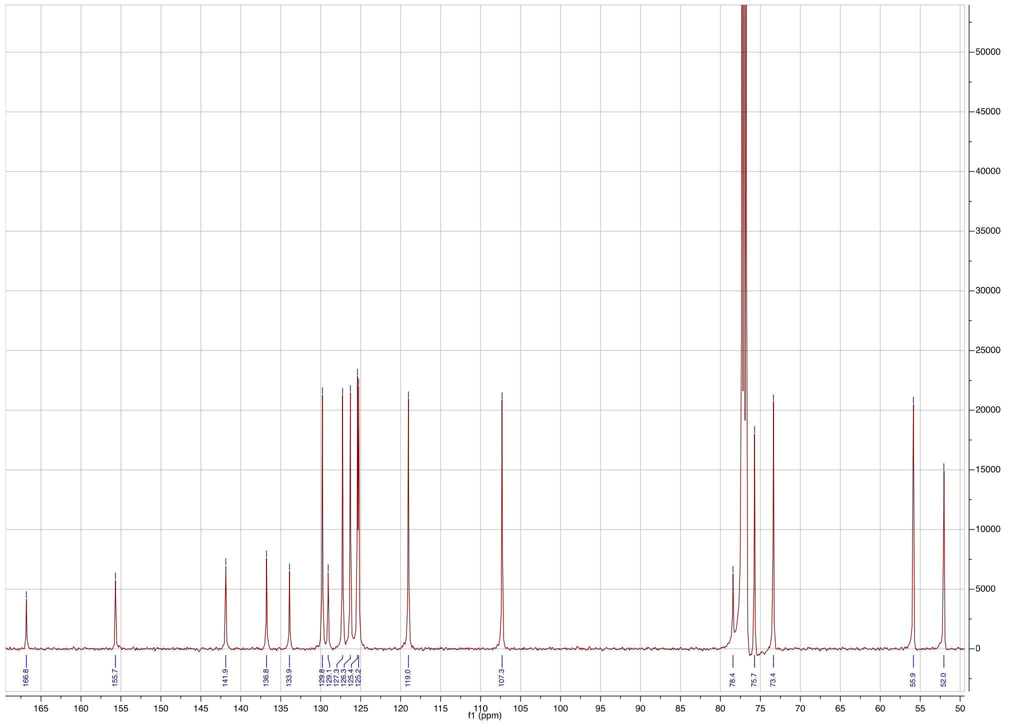

Figure ESI-29. ^{13}C NMR (125 MHz, $CDCl_3$) of methyl 2-[hydroxyl[6-(prop-2-ynyloxy)naphthalen-2-yl]methyl]acrylate (**6a**).

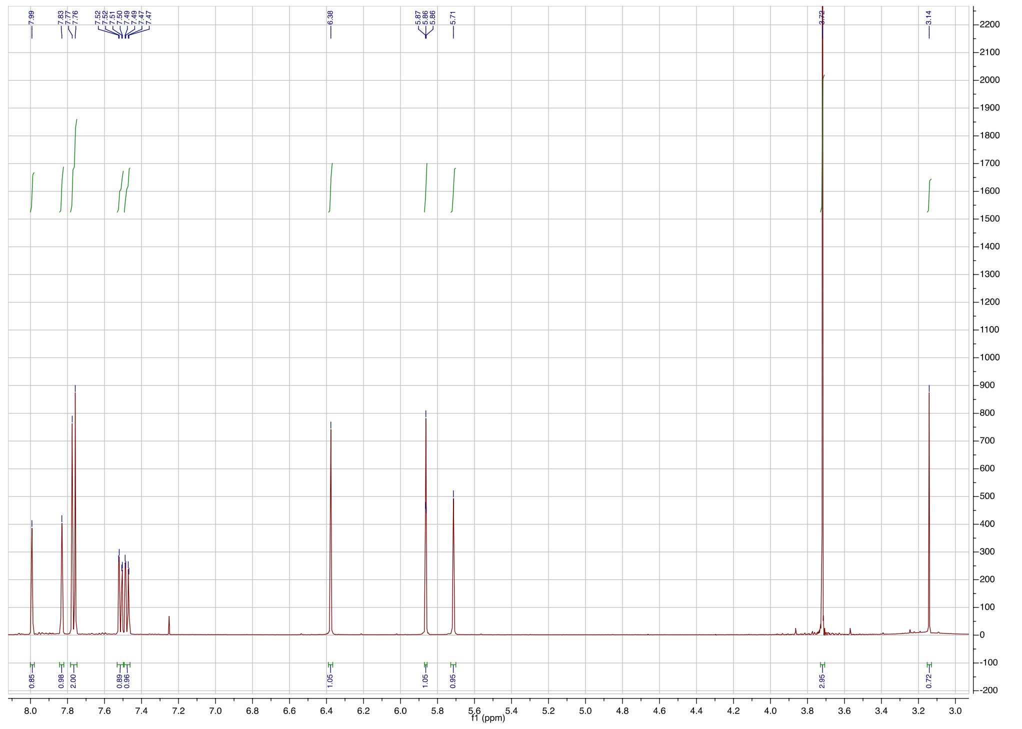

Figure ESI-30. ^{1}H NMR (500 MHz, $CDCl_3$) of methyl 2-[(6-ethynylnaphthalen-2-yl)(hydroxy)methyl]acrylate (**6b**).

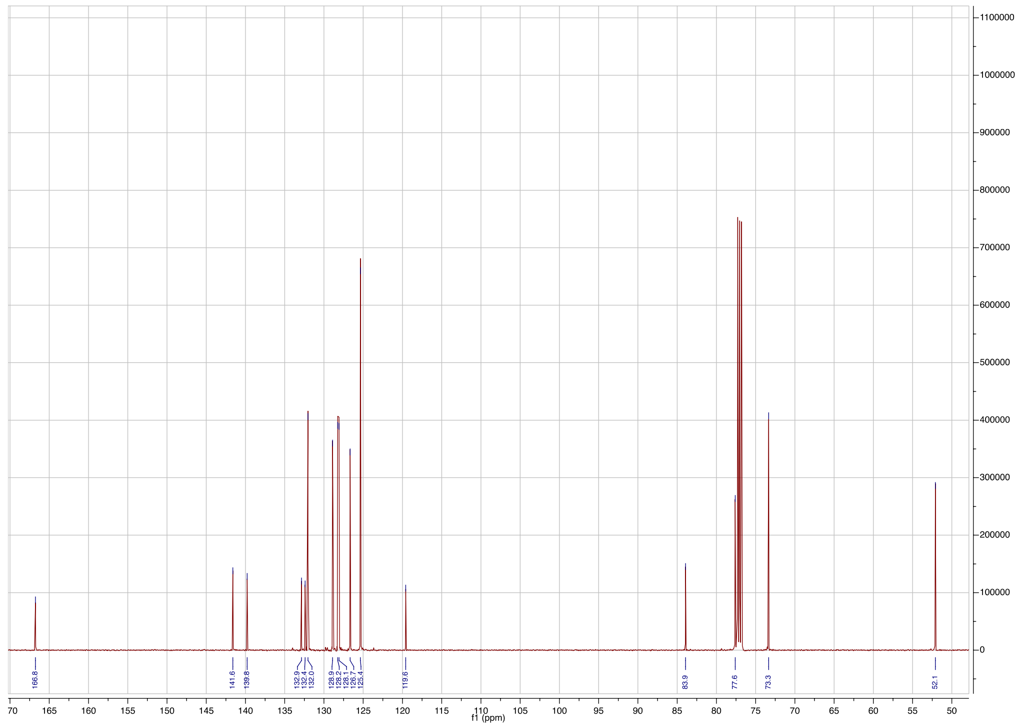

Figure ESI-31. ^{13}C NMR (125 MHz, $CDCl_3$) of methyl 2-[(6-ethynylnaphthalen-2-yl)(hydroxy)methyl]acrylate (**6b**).

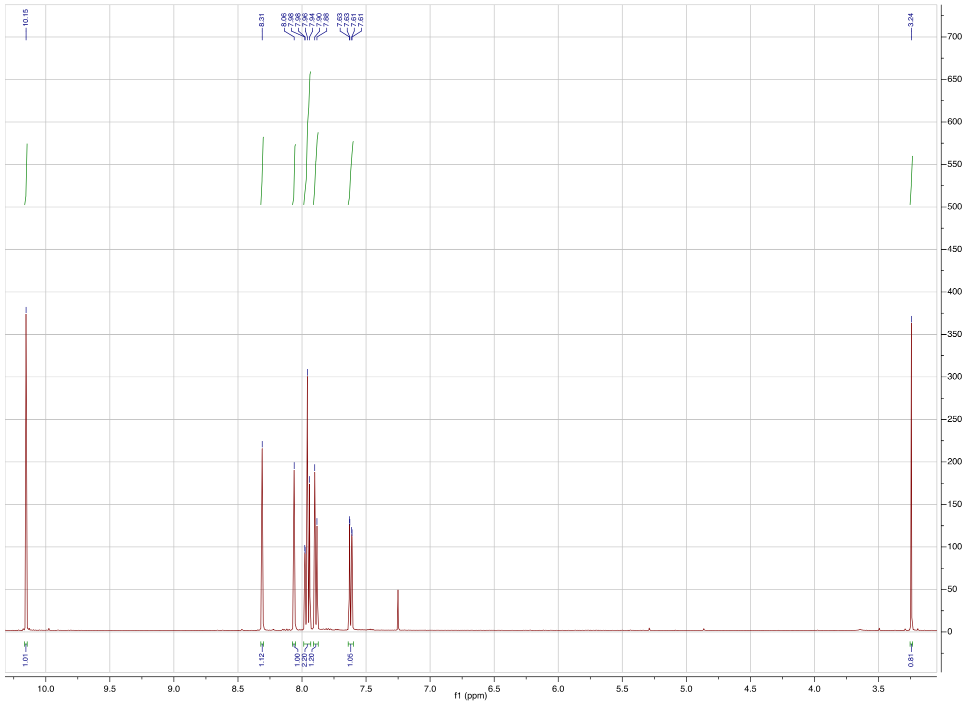

Figure ESI-32. ^{1}H NMR (500 MHz, $CDCl_3$) of 6-ethynyl-2-naphthaldehyde (**8**).

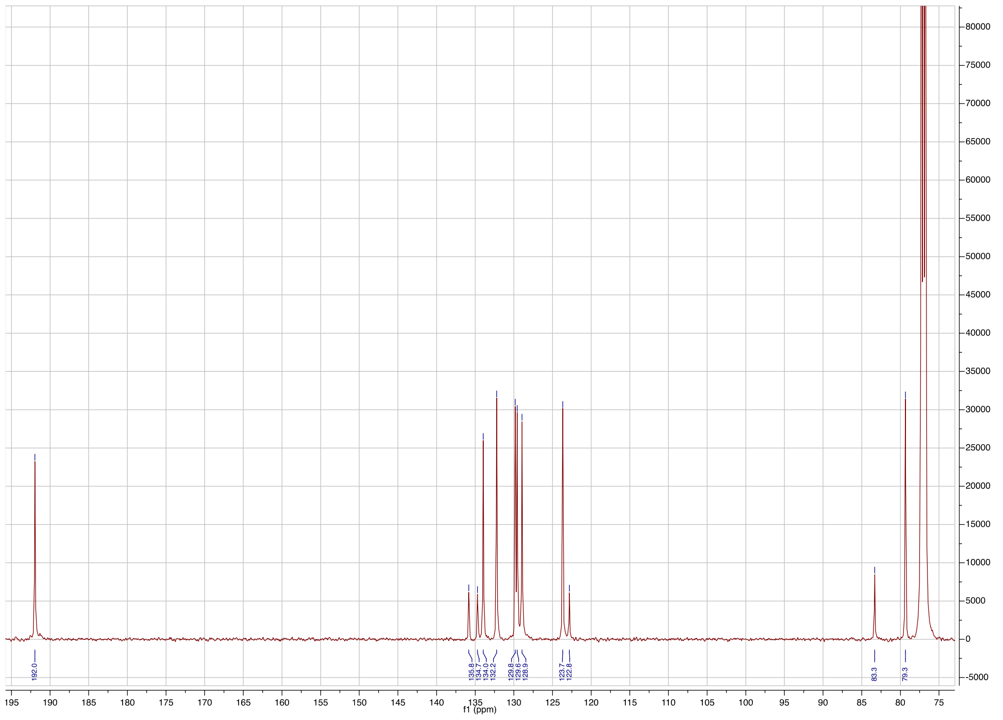

Figure ESI-33. ^{13}C NMR (125 MHz, $CDCl_3$) of 6-ethynyl-2-naphthaldehyde (**8**).

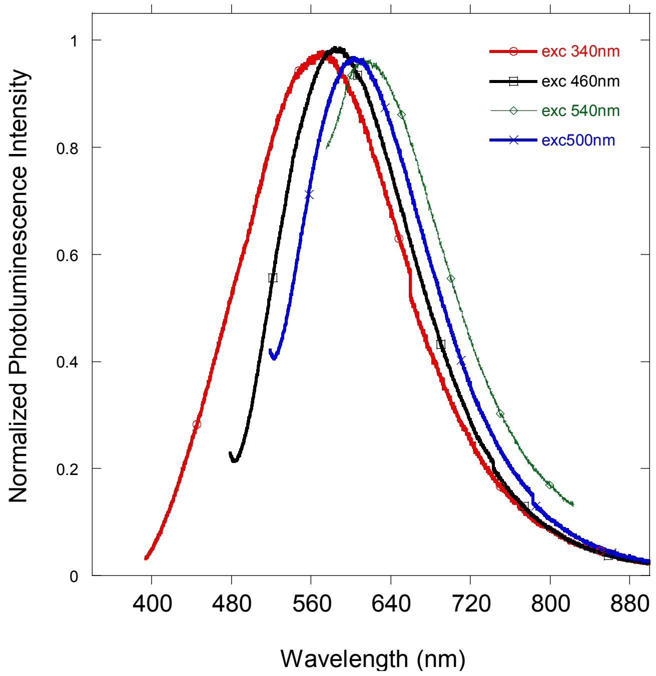

Figure ESI-34. Photoluninescence of polymeric material **Ac-His-6-MBHA-1d** obtained by exciting at different wavelengths.

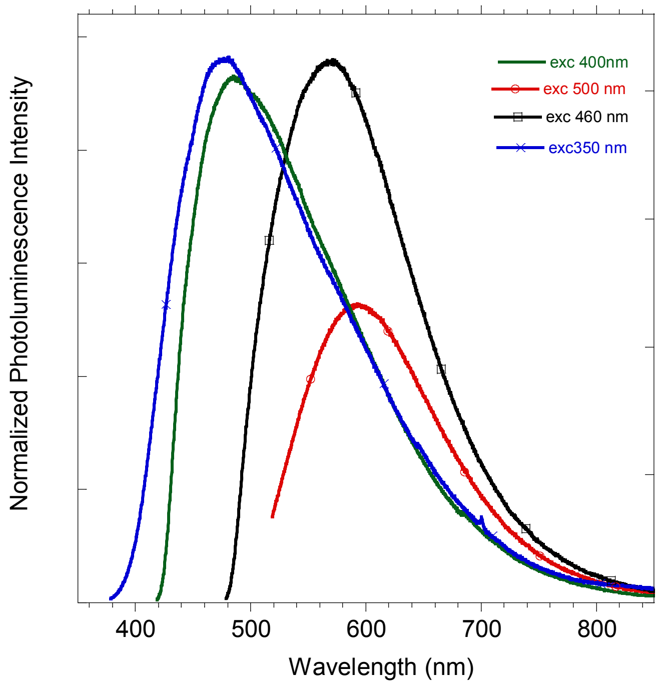

Figure ESI-35. Photoluninescence of polymeric material **Ac-His-6-MBHA-1e** obtained by exciting at different wavelengths.
